# SPE-44 Implements Sperm Cell Fate

**DOI:** 10.1371/journal.pgen.1002678

**Published:** 2012-04-26

**Authors:** Madhura Kulkarni, Diane C. Shakes, Katie Guevel, Harold E. Smith

**Affiliations:** 1 Department of Cell Biology and Molecular Genetics, University of Maryland, College Park, Maryland, United States of America; 2 Department of Biology, College of William and Mary, Williamsburg, Virginia, United States of America; 3 National Institute of Diabetes and Digestive and Kidney Diseases, National Institutes of Health, Bethesda, Maryland, United States of America; University of Wisconsin, Howard Hughes Medical Institute, United States of America

## Abstract

The sperm/oocyte decision in the hermaphrodite germline of *Caenorhabditis elegans* provides a powerful model for the characterization of stem cell fate specification and differentiation. The germline sex determination program that governs gamete fate has been well studied, but direct mediators of cell-type-specific transcription are largely unknown. We report the identification of *spe-44* as a critical regulator of sperm gene expression. Deletion of *spe-44* causes sperm-specific defects in cytokinesis, cell cycle progression, and organelle assembly resulting in sterility. Expression of *spe-44* correlates precisely with spermatogenesis and is regulated by the germline sex determination pathway. *spe-44* is required for the appropriate expression of several hundred sperm-enriched genes. The SPE-44 protein is restricted to the sperm-producing germline, where it localizes to the autosomes (which contain sperm genes) but is excluded from the transcriptionally silent X chromosome (which does not). The orthologous gene in other *Caenorhabditis* species is similarly expressed in a sex-biased manner, and the protein likewise exhibits autosome-specific localization in developing sperm, strongly suggestive of an evolutionarily conserved role in sperm gene expression. Our analysis represents the first identification of a transcriptional regulator whose primary function is the control of gamete-type-specific transcription in this system.

## Author Summary

Stem cells give rise to the variety of specialized cell types within an organism. The decision to adopt a particular cell fate, a process known as specification or determination, requires the coordinated expression of all of the genes needed for that specialized cell to develop and function properly. Understanding the mechanisms that govern these patterns of gene expression is critical to our understanding of stem cell fate specification. We study this process in a nematode species that makes both sperm and eggs from the same stem cell population. We have identified a gene, named *spe-44*, that is required for the proper expression of sperm genes (but not egg genes). Mutation in *spe-44* produces sterile sperm with developmental defects. *spe-44* is controlled by factors that govern the sperm/egg decision, and its function in controlling sperm gene expression appears to be conserved in other nematode species.

## Introduction

Stem cells have provoked tremendous interest because of their unique ability to differentiate into multiple cell types. The specification of a particular cell fate ultimately results in a program of cell-type-specific gene expression, and the identification and characterization of the regulators that mediate these transcriptional programs are a focus of intense research. Of particular note is the class of transcription factors that act as master switches; their activities are sufficient to dictate a particular cell fate by promoting, both directly and indirectly (via the regulation of additional transcription factors), the expression of the suite of cell-type-specific target genes. The canonical example is MyoD; heterologous expression is sufficient to convert a variety of cell types into myoblasts [1]. Master switch genes therefore specify as well as implement cell fate decisions.

The hermaphrodite germline of *Caenorhabditis elegans* offers an attractive model for investigating the regulation of stem cell fate specification and differentiation. Cell fate is restricted to a binary choice, sperm or oocyte, which greatly simplifies the analysis. The identical cellular milieu fosters the development of both types of gametes. The switch from spermatogenesis to oogenesis is genetically determined, but can be experimentally controlled using various temperature-sensitive mutations (reviewed in [2]) and chemical reagents [3]. Alternatively, germline stem cells can be manipulated to further expand their repertoire of potential fates, as recently demonstrated by their directed transdifferentiation into neurons [4].

The sexual fate of individual germ cells is specified by an elaboration of the same sex determination program that dictates male or hermaphrodite somatic development (reviewed in [5]). In the soma, that program culminates in the terminal regulator TRA-1, a homolog of cubitus interruptus and GLI transcription factors [6], [7]. TRA-1 promotes the hermaphrodite fate and inhibits male fate, and does so by direct repression of a number of transcription factors that, in turn, regulate sex-specific gene expression in a variety of somatic tissues including the intestine [8], the nervous system [9]–[11], the vulva [12], and the tail [13]. TRA-1 thereby acts as a classic master switch in specifying somatic sexual fate.

Within the germline of *C. elegans*, additional regulators modify this somatic sex determination program, in part to permit the production of male gametes in an otherwise female animal. In uncommitted germ cells, the FEM and FOG proteins function as the ultimate regulators of sexual fate. Sequence homology alone reveals little about the mode of FEM activity: *fem-1* encodes a protein with ankyrin repeats, *fem-2* a putative serine/threonine phosphatase, and *fem-3* a novel protein [14]–. Proteomic analysis has been more enlightening and shown that the FEM proteins are components of a CUL-2-dependent E3 ubiquitin ligase complex that targets TRA-1 for degradation [17]. FOG-1 is homologous to cytoplasmic polyadenylation element binding proteins, and presumably regulates translation of transcripts that govern gamete cell fate [18]. FOG-3 shares homology with the Tob/BTG family of antiproliferation proteins, and functions in both the initiation and maintenance of spermatogenesis [19]–[21].

The output of this germline sex determination program is gamete-type-specific gene expression. Microarray screening has identified thousands of genes that are differentially expressed in the germline during sperm or oocyte development [22], [23]. The functional significance of the observed transcription regulation is validated by the detection of genes known to be required for gamete development. For example, a large number of Spe (spermatogenesis-defective) genes have been isolated in mutational screens for sperm-specific sterility (reviewed in [24]), and essentially all of those genes were classified as sperm-enriched by microarray data. Transgenic studies indicate that transcriptional control is the primary mode of regulation for sperm genes [25].

Although germline sex determination ultimately governs sperm and oocyte-specific transcription, the precise mechanism of that regulation remains enigmatic. The TRA-1 transcription factor is an attractive candidate for the job, but, despite its demonstrated role in the soma, it does not appear to directly mediate sex-specific gene expression in the germline. First, TRA-1 loss-of-function mutants produce sperm and oocytes that are competent for fertilization and embryonic development [26], [27]; the only logical interpretation is that all of the genes essential for sperm and oocyte function continue to be expressed in the absence of TRA-1. Second, epistasis analysis demonstrates that the FOG proteins regulate gamete cell fate subsequent to TRA-1 [28], [29]. That observation is supported by the identification of the *fog-3* gene (and, most likely, *fog-1*) as a direct target of TRA-1 transcriptional regulation [30]. Finally, epistasis experiments likewise indicate that the FEM proteins govern germline sex determination in the absence of TRA-1 [31], [32]; as this function is obviously independent of their role in TRA-1 degradation, it is probable that additional targets exist. Therein lies the dilemma: the known transcription factor TRA-1 cannot directly regulate gamete-specific transcription, but the known downstream regulators do not encode transcription factors.

To date, the only identified regulator of *C. elegans* germline transcription is the GATA factor *elt-1*, which targets sperm genes [33]. However, that role does not appear to be its primary function. *elt-1* expression is not limited to sperm, and the gene is required for embryonic and larval development in a variety of tissues [34], [35]. The ELT-1 binding element is present in less than 5% of sperm genes, the majority of which comprise the *MSP* multigene family, and *elt-1* expression does not appear to be directly regulated by the sex determination program. Thus, the bulk of gamete-specific transcription is governed by as-yet-unidentified regulators.

This study provides a critical piece of the puzzle by identifying *spe-44* as a key regulator of sperm-specific transcription. Deletion of *spe-44* results in sperm-specific sterility and is associated with multiple defects in both the cell cycle and developmental programs of sperm differentiation. The *spe-44* gene is expressed in the germline at the onset of spermatogenesis, and is regulated by the terminal germline sex determination factors FEM-1, FEM-3, and FOG-1. SPE-44 promotes expression of a large fraction of sperm-enriched genes, and that role is likely conserved among nematodes. Our work highlights the mechanistic differences between sex determination in the soma, where TRA-1 specifies and directly regulates sex-specific transcription, and the germline, in which SPE-44 implements the transcriptional program specified by the FEM and FOG proteins.

## Results

### Identification of *spe-44*

A candidate gene approach was used to identify potential regulators of sperm gene expression. Previous microarray data characterized sex-specific transcriptional profiles for both the germline and soma [22], [23]. Eleven homologs of transcriptional regulators were among the 1,343 genes enriched in the sperm-producing germline (Table S1). We further restricted our list of candidates to those for which pre-existing mutations were available for functional characterization, since sperm genes as a group are generally refractory to inactivation by RNA interference [for example, see 33]. We selected *C25G4.4* for further study; the gene product is most similar to the mammalian glucocorticoid modulatory element-binding proteins GMEB-1 and -2 and encodes a SAND domain (for Sp100, Aire, NucP41/75, DEAF-1) [36]–[38]. SAND-containing proteins were first identified as transcriptional activators that bind to functional regulatory elements within the promoters of target genes [39], [40]. Solution and crystal structures of SAND domains reveal a novel DNA-binding fold centered on the highly conserved KDWK motif [41], [42]. The *C25G4.4* gene product contains all of the conserved residues of the SAND domain, including KDWK, but otherwise possesses no identifiable domains.

**Figure 1:**
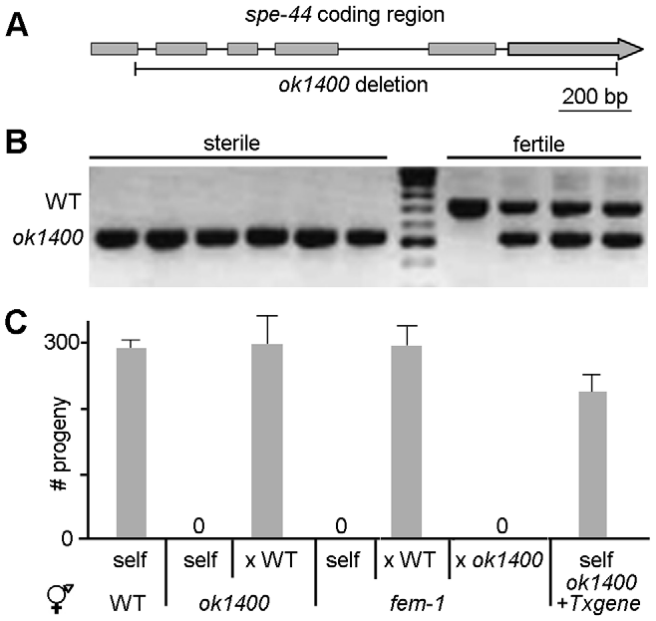
*spe-44(ok1400)* mutation causes sperm-specific sterility. A. Schematic diagram of *spe-44*. Genomic interval of C25G4.4; gray boxes indicate exons. The extent of the *ok1400* deletion is shown below. B. Linkage to sterile phenotype. Examples of individual sterile (N = 50) and fertile (N = 25) hermaphrodites screened by single-worm PCR for the wild-type (upper) and *ok1400* (lower) alleles. C. Fertility assays. Mean values and standard deviations of total progeny counts from individual hermaphrodites (N = 20). Hermaphrodite genotype is indicated at bottom. All male strains contained *dpy-20(e1282)* in addition to the indicated genotype. Self, unmated; x WT, crossed with wild-type males; x *ok1400*, crossed with *spe-44(ok1400)* males; + *Txgene*, unmated *spe-44(ok1400)* hermaphrodites containing the *spe-44* transgene.

The deletion allele *ok1400* of *C25G4.4* is almost certainly a null allele as it lacks 1577 of the 1797 base-pair coding region, including the conserved SAND domain (Figure 1A). The original strain isolated by the *C. elegans* Gene Knockout Consortium [43] produced both fertile and sterile progeny in roughly 3∶1 ratio, suggestive of heterozygosity for a locus conferring recessive sterility. When PCR was used to amplify the *C25G4.4* interval from individual sterile and fertile hermaphrodites, the sterile phenotype proved to be tightly linked to the deletion allele; all sterile animals were homozygous for *ok1400*, whereas all fertile animals contained at least one copy of the wild-type gene (Figure 1B). No additional defects in development or morphology were observed in the *ok1400* mutant, suggesting that *C25G4.4* might function exclusively in the context of reproduction.

In self-fertilizing hermaphrodites, sterility can arise from defects in sperm and/or oocyte function. To discriminate among those possibilities, we set up reciprocal matings between *ok1400* mutants and wild-type animals of the opposite sex (Figure 1C). Crosses between self-sterile *ok1400* homozygous hermaphrodites and wild-type males yielded viable progeny, indicating that *ok1400* hermaphrodites produce functional oocytes. By contrast, crosses between *ok1400* homozygous males [marked with the tightly linked *dpy-20(e1282)* allele] and *fem-1(hc17)* hermaphrodites (which lack sperm) produced no progeny, whereas *fem-1* hermaphrodites mated with homozygous *dpy-20(e1282)* males produced abundant outcross progeny. Microinjection of the *C25G4.4* transgene rescued the sterility of unmated *ok1400* homozygous hermaphrodites (Figure 1C, *+Txgene*), thereby confirming that the deletion allele is responsible for the observed sterility. Together these results indicate that the sterility of *ok1400* results from a defect in sperm function. Based on the sperm-specificity of its sterile phenotype, the *C25G4.4* gene was designated *spe-44* (for spermatogenesis-defective).

### Defects in spermatogenesis

Sperm cell development in *C. elegans* has been well characterized in both sexes [44]–[46]. Throughout the larval and adult stages, Notch signaling in the distal end of the gonad maintains a population of mitotically dividing germline stem cells with the potential to generate either oocytes or sperm [2]. During the third larval (L3) stage, the most proximal of these mitotic germ cells enter the meiotic cell cycle, an event that roughly coincides with the commitment of these cells to spermatogenesis [28]. By the fourth larval (L4) stage in both hermaphrodites and males, the various stages of spermatogenesis can be observed in a distal to proximal array within the gonad. Transcription of sperm genes typically initiates during the pachytene phase of the cell cycle [e.g., see 33]. Following the disassembly of the synaptonemal complex, the spermatocytes enter a karyosome stage during which global transcription ceases [46]. The primary spermatocytes then detach from the gonadal syncytium and undergo the two meiotic divisions. Cytokinesis during meiosis I can be complete or incomplete, producing secondary spermatocytes that contain one or two nuclei, respectively. Immediately following anaphase of meiosis II, cellular components that are not required for subsequent sperm function are partitioned away from the spermatids in a budding division and deposited in a residual body. In males, sperm production continues through adulthood, and immotile, spherical spermatids accumulate in the seminal vesicle. Upon insemination of hermaphrodites, the process of activation converts spermatids into mature, crawling spermatozoa that migrate from the uterus to the spermatheca. In hermaphrodites, gametogenesis switches abruptly from sperm to oocyte production at the L4/adult transition. Spermatids undergo activation into motile spermatozoa as they are propelled into the spermatheca ahead of the newly formed oocytes.

**Figure 2:**
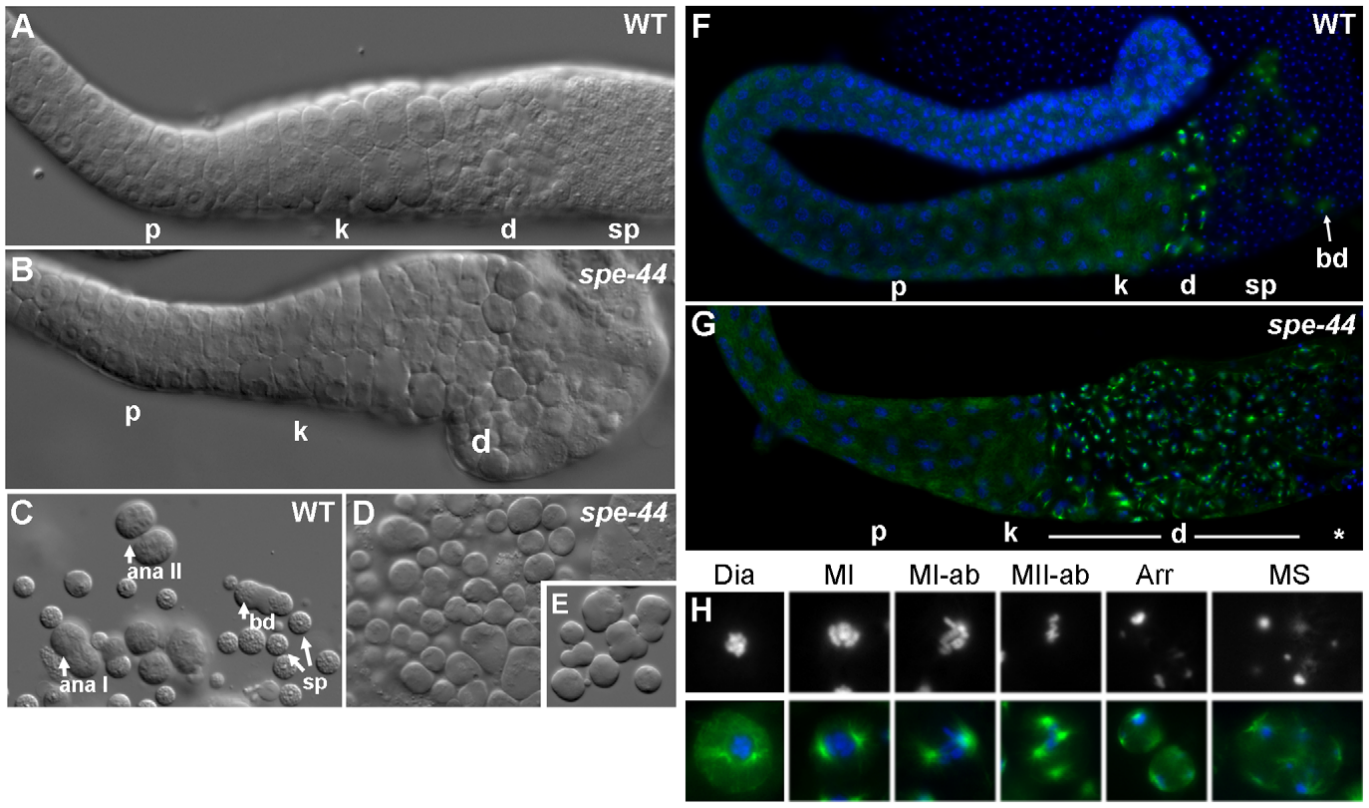
Spermatogenesis defects in *spe-44* males. A–B. Isolated gonads from wild-type (A) and *spe-44* (B) males visualized by DIC. Indicated are pachytene (p), karyosome (k), and meiotically dividing (d) spermatocytes, and spermatids (sp). C–E. Sperm spreads from wild-type (C) and *spe-44* (D–E) males. C. Wild-type spermatogenesis with spermatocytes in anaphase I (ana I), anaphase II (ana II), post-meiotic budding division (bd), and mature spermatids (sp). D. *spe-44* spermatogenesis with aberrantly dividing spermatocytes. E. Enlarged image of *spe-44*. F–G. Isolated gonads from wild-type (F) and *spe-44* (G) males co-stained with anti-tubulin (green) and DAPI (blue). Labeling as in panels A–B. Additionally indicated are residual bodies from the budding division (bd), and a mix of terminally arrested and dying spermatocytes (*). H. Higher magnification of individual sperm from *spe-44* males. Top row, DAPI staining to highlight nuclear morphologies; bottom row, merge of DAPI (blue) and tubulin (green). See text for labeling.

To characterize potential spermatogenesis defects, we examined adult male gonads, in which all stages of sperm development are readily visible, under differential interference contrast (DIC) optics. In wild-type gonads, spermatogenesis progressed in a distal-to-proximal array through the pachytene and karyosome stages of meiosis, followed by a meiotic division zone and then a region of packed spermatids (Figure 2A). The *spe-44* gonads (Figure 2B) were similar in overall size, suggesting that germline proliferation occured normally. Developing *spe-44* spermatocytes progressed appropriately through the pachytene and karyosome stages, although the mutant gametes were less refractile and thus smoother in appearance. However, the division zone was expanded, the cells misshapen, and normal spermatids failed to accumulate.

These differences were even more apparent in sperm spreads, which distinguish individual spermatocytes. In wild-type samples, the meiotic division zone could be further discriminated into spermatocytes undergoing anaphase I, anaphase II, and the post-meiotic budding division of spermatids from a residual body; numerous spermatids are also visible (Figure 2C). In the *spe-44* samples, dividing spermatocytes often exhibited a variety of cleavage defects, including unequal divisions and the formation of multiple partially budded structures, and no budding divisions of residual bodies were observed (Figure 2D–2E). Aberrant spermatogenesis was also observed in hermaphrodites (Figure S1). These morphological defects prompted a more detailed characterization of the *spe-44* phenotype.

To that end, we examined the chromatin and microtubule dynamics in male gonads (Figure 2F–2G). Similarly to DIC, no differences were observed between the germlines of wild-type and *spe-44* males through the karyosome stage. The meiotic division zone in wild-type gonads contained a small number of spermatocytes with visible asters and a few cells undergoing the budding division (in which tubulin segregates to residual bodies), followed by a large number of spermatids (Figure 2F). By contrast, *spe-44* gonads exhibit a greatly expanded meiotic division zone, suggesting a cell cycle arrest (Figure 2G).

Sperm spreads revealed a myriad of defects in the *spe-44* spermatocytes within the division zone (Figure 2H). Although the nucleation of asters initiated normally during diakinesis (Dia), the asters were often larger and broader than their wild-type counterparts. The asters migrated correctly to set up the metaphase I spindle (MI), but chromosome segregation was aberrant with most or all chromosomes segregating to one spindle pole (MI-ab), particularly during the second meiotic division (MII-ab). Examples of unequal chromosome segregation and extensive aneuploidy were also evident in arrested spermatocytes (Arr), and some of the smallest cells had microtubules but no chromatin (data not shown). As in wild-type spermatogenesis, both complete and incomplete cytokinesis occurred during the meiotic divisions; however, we frequently observed multiple spindles (MS), as many as eight per spermatocyte, instead of the typical two or four. The presence of these supernumerary asters is suggestive of spindle overduplication and/or fusion of terminally arrested spermatocytes. The asters in these terminal spermatocytes were almost always found adjacent to the plasma membrane, and the microtubules in these spermatocytes were often unusually long. Ultimately, the mutant spermatocytes appeared to lyse; the number of arrested spermatocytes did not increase dramatically with age, and cellular debris accumulated within the gonad (data not shown).

#### Cell cycle defects

**Figure 3:**
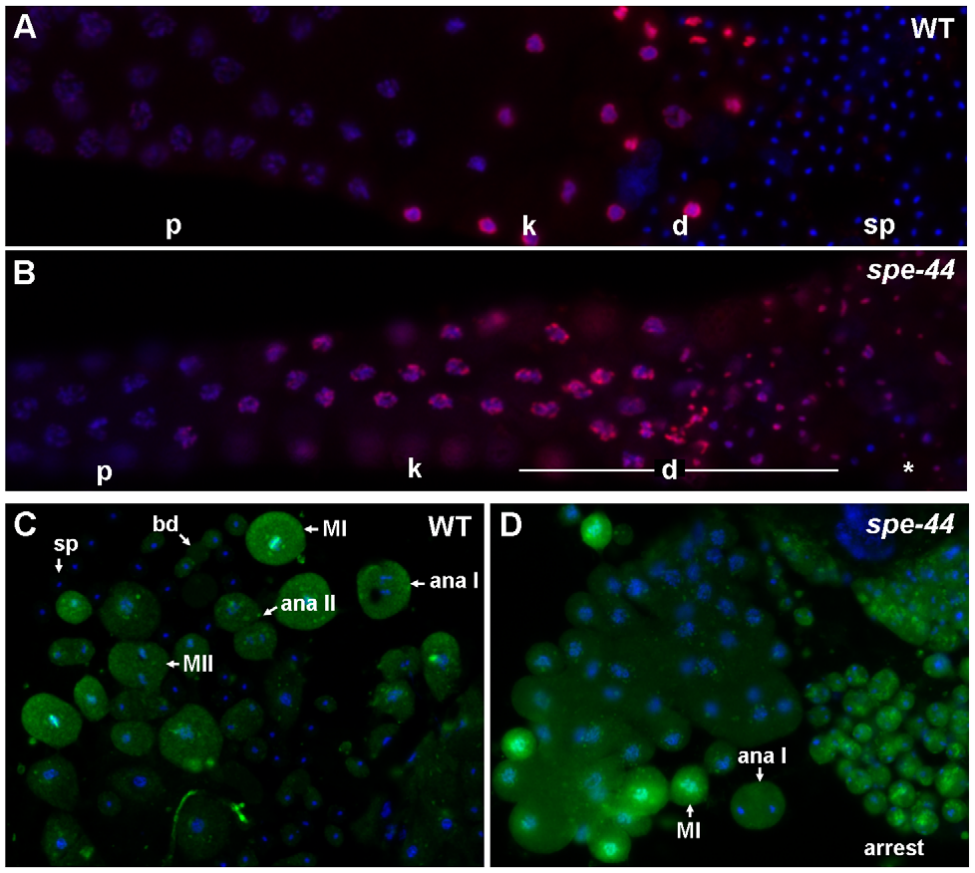
Cell cycle defects in *spe-44* sperm. A–B. Wild-type (A) and *spe-44* (B) male gonads co-stained with DAPI (blue) and anti-phosphorylated histone H3 Ser10 (red). C–D. Sperm spreads from wild-type (C) and *spe-44* (D) males stained with antibody MPM-2 (green) and DAPI (blue). Labeling as in Figure 2C. Additionally indicated are metaphase I (MI), metaphase II (MII), and arrested spermatocytes (arrest).

The striking persistence of microtubule asters in *spe-44* spermatocytes suggests an arrest of cell cycle progression. To test this hypothesis, we analyzed the pattern of histone H3 (serine 10) phosphorylation (anti-pHisH3), an indicator of Aurora kinase activity (Figure 3A–3B) [47]. In both *spe-44* and wild-type gonads, anti-pHisH3 labeling was first detectable in developing spermatocytes as the synaptonemal complex was beginning to break down, became most intense during the karyosome stage, and continued to label the chromosomes through the meiotic divisions. As wild-type spermatocytes completed anaphase II and initiated the budding division, anti-pHisH3 labeling of chromatin was no longer detectable (Figure 3A). By contrast, the chromatin of terminally arrested *spe-44* spermatocytes exhibited persistent anti-pHisH3 labeling (Figure 3B). Similar results were obtained with a marker of CDK-cyclin B phosphorylation. Staining of wild-type sperm spreads with monoclonal antibody MPM-2, which binds to a mitotic phospho-epitope of Cdk-CyclinB substrates [48], revealed labeling of the cytoplasm and metaphase I chromosomes during meiosis that was largely absent in spermatids (Figure 3C). In *spe-44*, labeling persisted in the terminally arrested spermatocytes (Figure 3D). These observations are indicative of an M-phase arrest of the cell cycle.

#### Organelle assembly defects

**Figure 4:**
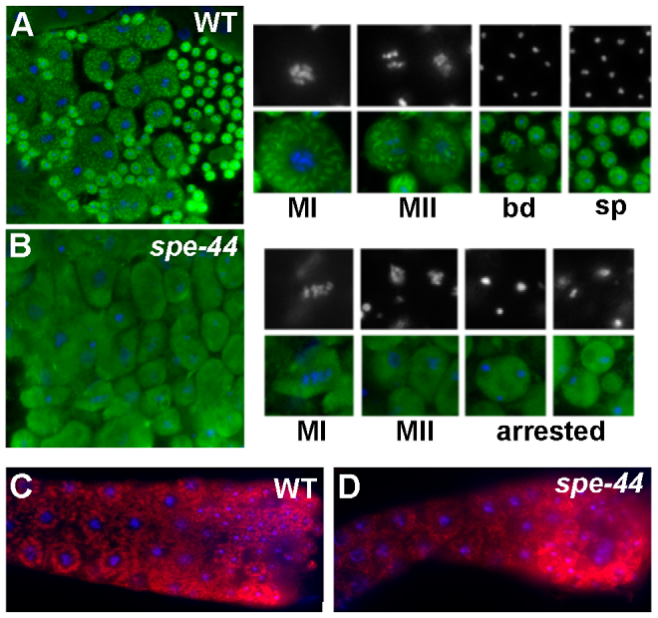
Organelle assembly defects in *spe-44* sperm. A–B. Wild-type (A) and *spe-44* (B) spermatocytes co-stained with anti-MSP (green) and DAPI (blue, white). Shown at right are individual sperm at higher magnification in metaphase I (MI), metaphase II (MII), the budding division (bd), mature spermatids (sp), and arrested spermatocytes (arrested). C–D. Wild-type (C) and *spe-44* (D) gonads co-stained with MO-specific 1CB4 antibody (red) and DAPI (blue).

If SPE-44 serves as a key regulator of the spermatogenesis program, *spe-44* spermatocytes might be expected to exhibit additional defects in the assembly of sperm-specific structures. Therefore, we examined the distribution and morphology of two sperm-specific components: assembly of the major sperm protein (MSP) into fibrous bodies, and the localization of Golgi-derived membranous organelles (MOs). Anti-MSP staining in wild-type sperm spreads (Figure 4A) revealed the formation of fibrous bodies, which appear as distinct puncta, early in meiosis. The fibrous bodies segregate into the spermatids during the budding division before becoming cytoplasmic in mature spermatids. In *spe-44* mutants, MSP failed to assemble into fibrous bodies but instead remained diffusely distributed in the cytoplasm throughout spermatogenesis (Figure 4B).

To investigate the formation and dynamics of the membranous organelles, we analyzed the immunostaining pattern of the monoclonal antibody 1CB4 [49]. In wild-type gonads (Figure 4C), MOs can first be detected as discrete structures in the late pachytene portion of the syncytial gonad; in spermatids, the MOs localized to the periphery adjacent to the cell membrane. In *spe-44* gonads (Figure 4D), the structures recognized by 1CB4 labeling appeared more disorganized, and distinct localization to the cell periphery was not observed. Thus, although the key components of the fibrous bodies and membranous organelles are present in the *spe-44* mutant, the assembly of these sperm-specific structures is aberrant.

### Sperm-specific expression of *spe-44*

**Figure 5:**
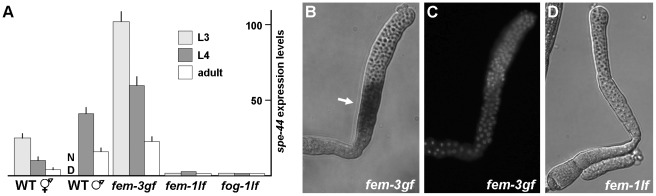
Expression and localization of *spe-44* mRNA. A. Quantification of *spe-44* expression by qRT-PCR. Transcript levels in each sample were normalized against actin as an internal control; the scale was arbitrarily set at one for the *fem-1* L3 value. Mean values and standard deviations are from triplicate samples of the indicated stage and genotype. ND, not determined. Genotypes from left to right are wild-type hermaphrodites, wild-type males, *fem-3(gf)* hermaphrodites, *fem-1(lf)* hermaphrodites, and *fog-1(lf)* hermaphrodites. B. *In situ* hybridization for *spe-44* transcript (arrow) in dissected *fem-3(gf)* L3 gonad. C. Same gonad stained with DAPI. D. *In situ* hybridization for *spe-44* in dissected *fem-1(lf)* L4 gonad.

Previous microarray data indicated that transcription of *spe-44* is elevated during spermatogenesis [22], [23], so we utilized quantitative RT-PCR to assess the dynamics of *spe-44* transcription during development. Expression of *spe-44* was first detected in age-synchronized populations of wild-type hermaphrodites at the L3 larval stage, when gametes become committed to the sperm cell fate (Figure 5A). Transcript levels of *spe-44* declined through the L4 and adult stages, when gametogenesis switches from sperm to oocyte production. In wild-type L4 and adult males, which produce sperm continuously, *spe-44* expression levels were higher than in hermaphrodites at the same stages (hermaphrodites and males are morphologically similar prior to L4, so males were not assessed at earlier larval stages).

To determine the role of the germline sex determination program in *spe-44* expression, we employed hermaphrodites that contained temperature-sensitive mutations in the terminal FEM and FOG regulators. When reared at the restrictive temperature, *fem-3(gf)* hermaphrodites produce only sperm. Expression of *spe-44* was observed beginning at the L3 stage and, although the levels declined somewhat, remained elevated into adulthood (Figure 5A). Conversely, the *fem-1(lf)* and *fog-1(lf)* mutations, which cause hermaphrodites to make only oocytes, resulted in low *spe-44* expression levels at all stages of development. Thus, *spe-44* transcript accumulation correlates strongly with the production of sperm. Furthermore, peak expression is observed during L3 at the onset of sperm fate specification. This pattern stands in marked contrast to that observed for other sperm genes. For example, *MSP*, which is abundantly expressed, is not detectable until L4 [50]. Likewise, a developmental time course of global transcription demonstrates that essentially all sperm genes exhibit a sharp increase at the onset of L4 and corresponding decline at the transition to adulthood [22], [23]. Thus, the peak expression of *spe-44* during L3 precedes that of other sperm genes, and is consistent with a role in the regulation of sperm gene expression. Finally, *spe-44* expression is governed by terminal regulators of the germline sex determination pathway (*fem-1*, *fem-3*, and *fog-1*) that specify sperm or oocyte fate.

*In situ* hybridizations of dissected gonads were performed to ascertain the precise spatial pattern of *spe-44* expression. The *fem-3(gf)* and *fem-1(lf)* mutations were used to restrict hermaphrodite gametogenesis to sperm or oocyte production, respectively. Abundant expression was first detected in *fem-3(gf)* animals during the L3 stage in the early meiotic germline (Figure 5B). DAPI staining of the chromatin indicated that expression coincides with early pachytene of meiosis I (Figure 5C). By contrast, *spe-44* expression was undetectable in *fem-1(lf)* germlines at any stage of development (Figure 5D). Thus, *spe-44* expression is restricted to the sperm-producing germline at a time and place consistent with the regulation of sperm gene expression.

### Regulation of sperm gene transcription by SPE-44

Since SPE-44 shares homology with known transcriptional regulators, we performed DNA microarray screening to assess its role in governing gene expression. Comparison between wild-type and *spe-44* L4 males at the onset of sperm production revealed statistically significant differences (p<0.05) in gene expression between the two samples. A total of 813 genes exhibited greater than two-fold changes in expression. The levels of 535 genes were reduced in the *spe-44* males and 278 were elevated (Table S2).

**Figure 6:**
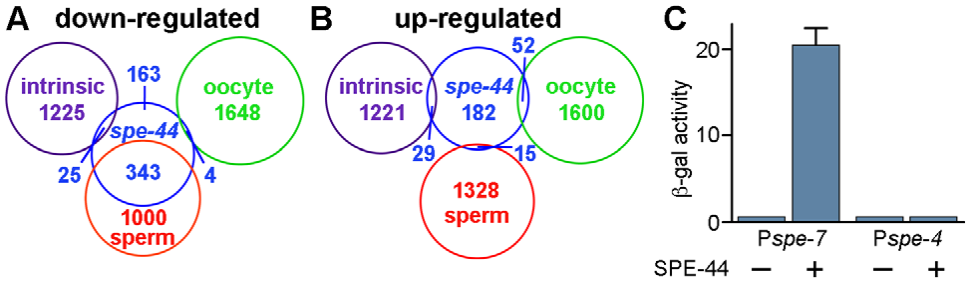
Summary of microarray results and transcriptional activation by SPE-44. A–B. Summary of microarray results. Venn diagrams of the genes that were down-regulated (A) or up-regulated (B) in *spe-44* mutant males compared to the categorization (sperm-enriched, oocyte-enriched, or germline intrinsic) defined in [23]. C. Transcriptional activation by SPE-44. Expression of *lacZ* reporters containing the indicating promoter plus *spe-44* under repression (−) or induction (+). ß-galactosidase activity quantified by ONPG assay for triplicate samples under each condition.

We compared our *spe-44* microarray data to previous transcriptional profiles that classified genes as sperm-enriched, oocyte-enriched, or germline-intrinsic (i.e., expressed in both sperm and oocyte) [22], [23]. Of the 535 genes that were down-regulated in the *spe-44* strain, nearly two-thirds (343) were also classified as sperm-enriched (Figure 6). Note that the degree of overlap between the data sets is likely under-represented, in part due to technical differences between the microarray platforms that were employed. By contrast, very little concordance was observed with either the oocyte-enriched (four) or germline-intrinsic (25) classes. Therefore, loss of *spe-44* results in a substantial defect in sperm-enriched gene expression. Those genes are hereafter referred to as *spe-44* sperm targets.

We performed the same comparison with the 278 genes that exhibited increased expression in the *spe-44* strain. No significant overlap was observed with any of the three germline categories (Figure 6). These results indicated that germ cell fate in *spe-44* mutants is not shifted from sperm to oocyte; such a fate switch would be accompanied by an increase in oocyte-enriched transcription. Therefore, *spe-44* is not a component of the sex determination pathway that specifies gamete cell fate; however, it is required to implement that fate by promoting the transcription of sperm genes.

We also examined the frequency of GO terms associated with the sets of genes that were down or up-regulated in the *spe-44* mutant strain. Among the genes with reduced expression levels, three categories were over-represented: protein kinases (3.2-fold higher than expected) and associated terms (e.g., protein amino acid phosphorylation); protein phosphatases (4.3-fold) and associated terms; and structural molecules (8.6-fold) (Table S3). All of these categories are consistent with a defect in sperm gene expression. The overabundance of protein kinases and phosphatases among the sperm transcriptome has been reported previously [22]; the structural molecules largely consist of the *MSP* and *SSP* gene families, which are structural components of the sperm motility apparatus (see below). Similar characterization of the genes that were up-regulated in the *spe-44* strain was not as informative. The only two over-represented terms were ATP-binding (2.3-fold higher than expected) and intracellular (2.2-fold) (Table S4).

The list of *spe-44* down-regulated sperm targets contains a significant number of genes with demonstrable roles in sperm (shown in Table 1). The genes fall into four categories: 1) MSP (major sperm protein) is the structural polymer responsible for nematode sperm motility, and also serves as a signaling molecule to promote ovulation and oocyte maturation [51], [52]. A subset of the multigene *MSP* family (17 of 28 members) exhibits greater than two-fold reduction in transcript levels in the *spe-44* mutant. 2) Members of the small sperm-specific protein family (formerly SSP; designated *ssp/ssq/ssr/sss*) are structurally similar to MSP and play a role in MSP polymerization [53], [54]; five are found among the *spe-44* targets. 3) Spe (spermatogenesis-defective) and Fer (fertilization-defective) genes have been identified in genetic screens for sperm-specific sterility [55]–[57]; four Spe genes are targets of *spe-44*. 4) The *elt-1* gene, which encodes a GATA transcription factor that regulates the transcription of a subset of sperm genes [33], is down-regulated in the *spe-44* mutant strain. Because all of these genes have known functions within sperm, the reduced transcript levels observed in the *spe-44* mutant may contribute to the variety of defects that occur during spermatogenesis (see Discussion).

**Table 1:** Known sperm genes among SPE-44 targets.

Gene Name	Fold reduction in expression
*msp-3*	−3.09339
*msp-19*	−2.25681
*msp-31*	−2.32866
*msp-33*	−4.09991
*msp-40*	−2.33318
*msp-45*	−6.42015
*msp-49*	−5.90441
*msp-50*	−5.92461
*msp-51*	−2.12545
*msp-53*	−2.02062
*msp-59*	−2.3348
*msp-63*	−3.41096
*msp-64*	−3.69385
*msp-74*	−2.74553
*msp-113*	−2.22367
*msp-142*	−2.16431
*msp-152*	−3.37188
*ssp-10*	−2.40201
*ssp-19*	−15.699
*ssp-31*	−7.84404
*sss-1*	−103.649
*sss-2*	−17.8787
*spe-7*	−27.3584
*spe-10*	−3.30564
*spe-17*	−2.19598
*spe-27*	−9.99076
*elt-1*	−6.64459

The target genes identified by microarray might be directly or indirectly regulated by SPE-44. The multigene *MSP* family is likely to fall within the latter category. Prior work identified the GATA transcription factor ELT-1 as a direct regulator of *MSP* expression [33]. Our microarray data indicate that both *elt-1* and the *MSP* genes are expressed at lower levels in the *spe-44* mutant. The simplest model is a transcriptional cascade in which SPE-44 promotes *elt-1* expression, whose product in turn promotes *MSP* transcription. Alternatively, SPE-44 might work in conjunction with ELT-1 to promote maximal levels of *MSP* transcription and/or to restrict expression to sperm.

We tested the ability of SPE-44 to directly promote transcription in a heterologous system, using a variant of the yeast one-hybrid assay [58]. We constructed a yeast *lacZ* reporter gene that contained the putative promoter region of *spe-7* (P*spe-7::lacZ*), a sperm target gene that is strongly down-regulated in the *spe-44* mutant strain. Expression of *spe-44* was controlled by the galactose-inducible *GAL1* promoter. Induction of *spe-44* resulted in P*spe-7*::*lacZ* expression as measured by ß-galactosidase activity (Figure 6B). Expression of P*spe-7*::*lacZ* was dependent upon *spe-44*, as ß-galactosidase activity was not detectable under non-inducing conditions. We also examined the specificity of SPE-44 transcriptional activation for its target promoter. No expression was observed from a *lacZ* reporter that contained the promoter region of *spe-4*, a sperm gene that is not a target of *spe-44*. Therefore, SPE-44 can function as a transcription factor to directly activate gene expression from its cognate promoter.

### Association of SPE-44 with chromatin

**Figure 7:**
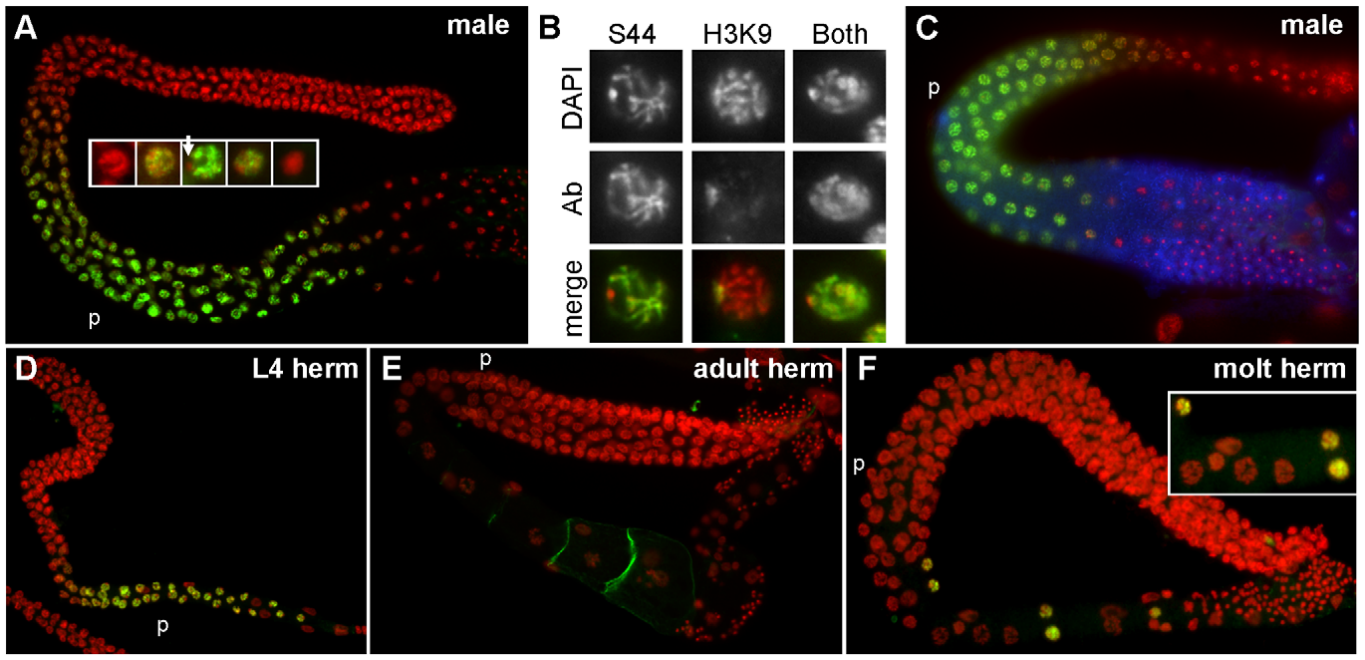
Pattern of SPE-44 protein expression and localization. A. Wild-type adult male gonad co-stained with anti-SPE-44 (green) and DAPI (red). Insert at higher magnification shows details of nuclear morphologies during the appearance/disappearance of SPE-44 labeling. Arrow indicates unlabeled DNA. B. Individual nuclei from DAPI-stained wild-type male gonads that were co-stained with anti-SPE-44 only (S44), anti-dimethylated histone H3 (Lys9) antibody only (H3K9), or both antibodies. Top row, DAPI; middle row, antibody (Ab); bottom row, merged image. C. Wild-type male gonad stained with anti-SPE-44 (green), anti-MSP (blue), and DAPI (red). D–F. Wild-type hermaphrodite gonads stained with anti-SPE-44 (green) and DAPI (red). D. L4 larval stage. E. Adult stage. F. Spermatogenesis to oogenesis transition at the L4/adult molting stage. Insert shows details of the first oocyte nuclei interspersed with a few last SPE-44-staining nuclei. The pachytene region (p) is indicated for orientation.

To determine if the SPE-44 protein localized to chromatin *in vivo*, we generated a SPE-44 antibody and stained dissected gonads. The timing and distribution agreed with our previous transcriptional analysis, and correlated perfectly with sperm production. Temporally, SPE-44 labeling was first detectable in L3 male germlines and persisted through adulthood (Figure S2 and Figure 7A). Spatially, SPE-44 labeling was absent in the distal stem cell niche and mitotic zone, initiated in the early meiotic zone, co-localized with chromosomes in the pachytene region, and became non-chromosomal before disappearing altogether in karyosome stage nuclei (Figure 7A; see inset for details). The observed staining pattern is dependent upon the SPE-44 protein, as it is absent in the *spe-44* mutant strain (Figure S3).

Closer examination revealed that SPE-44 localized along the length of most but not all of the pachytene chromosomes (Figure 7A, inset, and 7B). The unpaired X chromosome was a likely candidate for SPE-44 exclusion: X is singularly devoid of sperm genes, and contains chromatin modifications consistent with transcriptional silencing [22], [59]. To test this hypothesis, we compared the distribution of SPE-44 and histone H3(lysine 9) dimethylation, a chromatin modification that specifically labels the X chromosome in the male gonad (Figure 7B). Co-incubation with both antibodies labeled all chromosomes, indicating that the chromosome that is unlabeled by SPE-44 is indeed the X (Figure 7B). The localization of SPE-44 to autosomes (which contain sperm genes) but not X (which does not) is consistent with a role as an early acting and positive regulator of sperm gene transcription.

For sperm targets of SPE-44, we predict that their distribution would be coincident with or subsequent to the appearance of SPE-44. Therefore, we examined the localization of SPE-44 in comparison to MSP, an abundant sperm-specific marker that is a target of SPE-44 (Table 1). Co-staining clearly demonstrated that SPE-44 is detectable prior to the accumulation of MSP (Figure 7C), which is first observed near the bend of the gonad at mid to late pachytene. Thus, SPE-44 exhibits the properties predicted for a regulator of sperm gene expression: the protein appears at the onset of sperm fate specification prior to the production of sperm proteins, is bound to the chromatin of committed and developing spermatocytes, and disappears in the karyosome stage coincident with the global cessation of transcription.

Labeling of SPE-44 in hermaphrodite gonads likewise correlates with sperm production. The protein is first detectable in L3 at the time of sperm fate specification (Figure S4) and in young L4 hermaphrodites undergoing spermatogenesis (Figure 7D), but is absent in adult hermaphrodites that are undergoing oogenesis (Figure 7E). We were particularly interested in the distribution of SPE-44 during the switch in gamete fate. An intriguing property of the *C. elegans* hermaphrodite gonad is its ability to cleanly transition from the production of spermatocytes to the production of oocytes without making sexually ambiguous gametes. To test whether SPE-44 might be functioning in this switch, we examined the pattern of SPE-44 localization in hermaphrodite gonads during this developmental transition. Remarkably, in the proximal gonads of hermaphrodites that contained spermatocytes directly adjacent to the first enlarging oocytes, we always observed a few SPE-44 staining nuclei interspersed among these initial oocytes (Figure 7F). Although we have not ruled out the possibility that these are residual SPE-44-positive nuclei displaced from the syncytial germline by enlarging oocytes, we favor the interpretation that these are developing spermatocytes, and that SPE-44 is a marker of gamete sexual fate that is regulated at the level of individual cells. As an aside, we note that anti-SPE-44 labeling of the hermaphrodite nuclei co-localizes with most but not all of the chromatin, and (as demonstrated in males) the unlabelled region presumably represents the two X chromosomes.

### Conservation of *spe-44*

**Figure 8:**
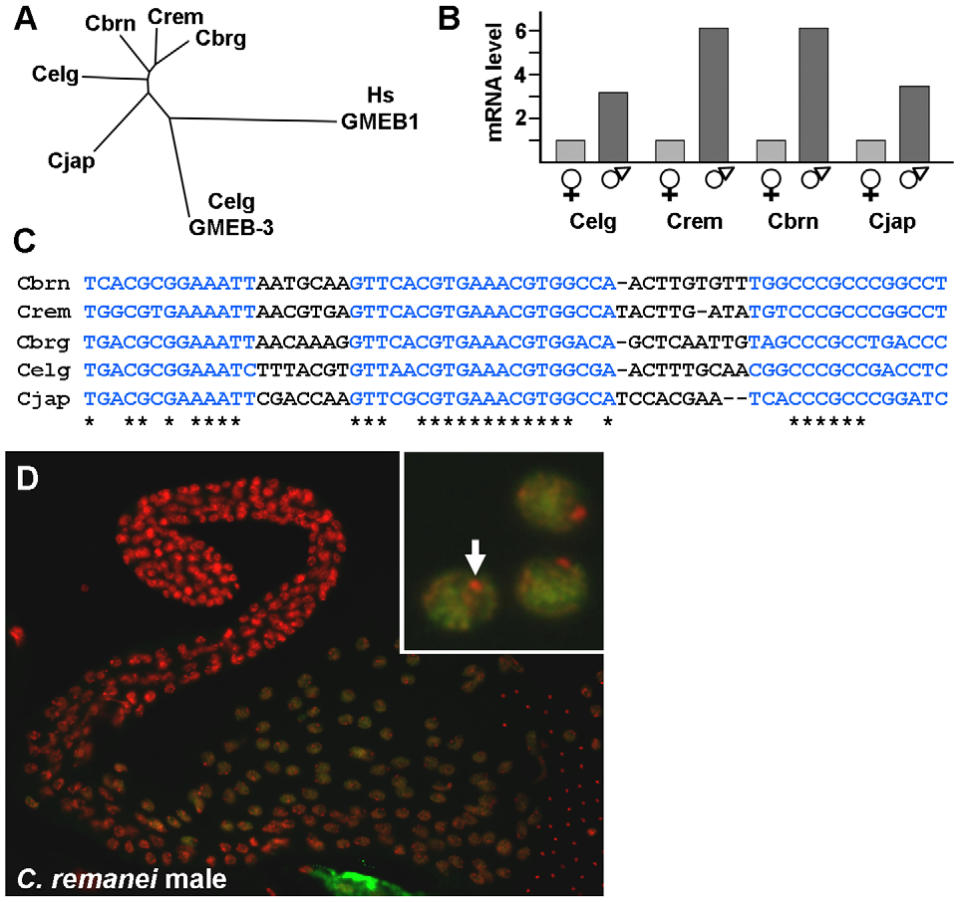
*spe-44* conservation in *Caenorhabditis* species. A. Tree indicating the relationship of the SPE-44 orthologs from the indicated species. CLUSTALW2 [81] was used to perform multiple sequence alignment of complete amino acid sequences for the following proteins: Wormbase accession numbers CN01523 (*C. brenneri*; abbreviated Cbrn), RP47564 (*C. remanei*; Crem), CBP08202 (*C. briggsae*; Cbrg), CE05305 (*C. elegans*; Celg), and JA05534 (*C. japonica*; Cjap). Also included were the paralogous GMEB-3 from *C. elegans* (accession number CE31443; Celg GMEB-3), and human GMEB1 as an outgroup (NCBI accession NP_077808; Hs GMEB1). Branch length reflects the degree of similarity. B. Sex-biased expression. Relative read number from RNA-Seq data; female/hermaphrodite values were normalized to one for each species. *Celg, C. elegans; Crem, C. remanei; Cbrn, C. brenneri, Cjap, C. japonica*. C. Conservation of *spe-44* upstream sequences. Blue indicates conserved sequence elements; asterisks indicate identical nucleotides. D. SPE-44 protein localization in *C. remanei*. Dissected male gonad co-stained with anti-SPE-44 antibody (green) and DAPI (red). Higher magnification (inset) reveals exclusion from a single DNA focus, presumably X (arrow).

All nematode species share an unusual mode of sperm motility (amoeboid crawling) and a novel protein (MSP) that underlies that motility. Similarly, the regulation of sperm gene expression might be conserved among nematodes. Complete genomes are available for five species of the *Caenorhabditis* group, so those sequences were screened for *spe-44*-related genes. *C. elegans* itself contains a paralagous gene, *gmeb-3*, whereas each of the remaining species contains a single *spe-44* homolog. These homologs encode proteins that are more similar to SPE-44 than GMEB-3 and likely orthologous, and the tree agrees well with the current phylogeny of these species (Figure 8A). Sequences related to *spe-44* were also identified in more distant nematode species (data not shown); however, conservation was limited largely to the SAND domain, which is represented in several *C. elegans* genes, and the degree of similarity was insufficient to distinguish the relationship unambiguously. Although the extent of *spe-44* conservation across the phylum is unknown, the gene is clearly conserved within the *Caenorhabditis* group.

An examination of sex-biased transcription in species of the *Caenorhabditis* group is currently underway (C. Thomas, manuscript in preparation), so those RNA-Seq data were examined for differences in *spe-44* expression. In all four species under study (*C. elegans*, *C. remanei*, *C. brenneri*, and *C. japonica*), the *spe-44* ortholog exhibited significant male-biased expression (Figure 8B). Note that the degree of male-biased expression may be greater than indicated by these data, which are derived from adult animals; in *C. elegans*, peak levels of *spe-44* mRNA are observed in L3 larvae and decline through the L4 and adult stages (Figure 5A). Although the developmental profiles of *spe-44* transcription in the other species are currently unknown, the data from adults clearly demonstrate that male-biased expression is a conserved property of *spe-44*.

The conservation of gene expression patterns among species can be reflected in the conservation of promoter elements that regulate transcription. Therefore, we performed multiple sequence alignment of the upstream regions from the five *spe-44* orthologs. We discovered three highly conserved sequence elements (Figure 8C, in blue) within 150 nucleotides of the initiation codon, which could potentially represent binding sites for unknown regulatory factors. Thus, the sequence conservation of *spe-44* is not limited to the coding sequences but extends to putative promoter elements.

Another distinctive property of SPE-44 in *C. elegans* is its restriction to meiotic autosomes in developing sperm. To determine if this distribution pattern is likewise conserved, isolated gonads from *C. remanei* males were immunostained with the anti-SPE-44 antibody (which recognizes a carboxy-terminal epitope whose sequence is conserved among the *spe-44* orthologs). As in *C. elegans*, we observed SPE-44 labeling of the chromatin of pachytene nuclei, with the notable exception of the presumed X chromosome (Figure 8D, arrow in inset). The conservation of gene expression and protein distribution in both male/hermaphrodite (*C. elegans*) and male/female (*C. remanei*, *C. brenneri*, and *C. japonica*) species strongly suggests that the observed pattern represents the ancestral state among this group of nematodes. Although we have yet to directly demonstrate that *spe-44* is required for spermatogenesis and sperm gene regulation in the other species, the degree of sequence conservation within the putative promoter and coding sequence, coupled with male-enriched expression and the pattern of protein distribution in the germline, makes a compelling case for an evolutionarily conserved role.

## Discussion

This work identifies *spe-44* as a critical regulator of sperm-specific transcription in the *C. elegans* germline. Mutation of *spe-44* results in sperm-specific sterility, a consequence of aberrant sperm development that includes cell cycle arrest and defects in sperm organelle assembly. The *spe-44* gene is expressed in the sperm-producing germline at the onset of sperm fate specification, and its product is required for the appropriate expression of several hundred sperm-enriched transcripts. Targets of *spe-44* include a number of genes known to be required for sperm development. The *spe-44* gene and promoter sequence, male-biased expression pattern, and germline protein distribution are conserved among nematode species, consistent with an evolutionarily conserved role in the regulation of sperm gene expression. *spe-44* lies at the terminus of the germline sex determination pathway, and its primary role is to implement cell fate specification by promoting sperm-specific transcription.

The *spe-44* deletion phenotype is complex and multifaceted, as might be expected for an early acting factor that regulates a large number of sperm-specific genes. Sperm cell fate is specified appropriately in *spe-44* mutants, but these gametes are unable to properly implement the programs of meiotic cell division and differentiation. Initiation of meiosis appears normal, but the ensuing defects in chromosome segregation suggest that *spe-44* spermatocytes either lack, or are unable to properly regulate, key spindle and kinetochore components necessary for chromosome attachment. Ultimately, *spe-44* spermatocytes arrest at M-phase and fail to undergo the budding division. The relationship between these two events is unclear. Cell cycle arrest might block progression of the budding division (e.g., via a checkpoint mechanism); alternatively, the segregation of cell cycle regulatory factors (perhaps in association with the meiotic spindles) to the residual body might be required for the completion of meiosis. In either case, the defects in fibrous body and membranous organelle assembly likely contribute to the failure of the budding division. FBs are composed of MSP assembled in a paracrystalline array, and MOs contain a variety of secretory and membrane proteins. FB-MO complexes are thought to facilitate the segregation of these critical components into the budding spermatid and away from the residual body, so the failure of this partitioning event in *spe-44* mutants is not surprising.

The observed phenotypes presumably reflect reduced levels of multiple components necessary for specific aspects of spermatogenesis. The *spe-44* mutation causes a defect in the expression of several hundred sperm genes, which are obvious candidates for those functions. For example, mutations in the *spe-44* target *spe-7* are associated with defects in fibrous body assembly similar to those observed in *spe-44* mutants [M. Presler, K. Messina, and D. Shakes, pers. comm.]. The reduction in *MSP* expression might also contribute to this phenotype. However, any effect may be modest, as the *spe-44* mutation targets only a subset of the *MSP* genes and their expression is reduced but not abrogated. Additional targets of *spe-44* that might contribute to the membranous organelle assembly defects include *spe-10* and *spe-17*, which are required for both the structural integrity of the membranous organelles and the proper segregation of components into the residual body during spermatid formation [60], [61]. Similarly, M-phase arrest might reflect reduced expression of the *spe-44* target CDC14, a phosphatase that normally functions to inactivate cyclin-dependent kinase and promote M-phase exit [62].

SPE-44 was selected as a candidate transcriptional regulator on the basis of its SAND domain. SAND domain proteins exhibit intrinsic transcriptional activation ability in a variety of reporter assays, a property shared by SPE-44 (Figure 6C). However, these proteins appear to function primarily through interactions with other transcriptional regulators. In some cases, the binding partners are canonical transcription factors. For example, GMEB-1 and GMEB-2 function with the glucocorticoid receptor to modulate target gene expression [63], while DEAF-1 cooperates with the Hox gene Deformed [40]. SAND domain proteins have also been implicated in chromatin-mediated regulation of transcription. AIRE is part of a large protein complex that includes various chromatin components [64], SP100 protein interacts with HMG and HP-1 heterochromatin proteins [65], [66], and GMEB binds the histone acetylase CBP [63]. In plants, ULTRAPETALA1 has been shown to function in the Trithorax group chromatin remodeling complex to antagonize the repressive effects of histone methylation by the Polycomb complex [67]. The observed distribution pattern of SPE-44 suggests that it, too, interacts closely with chromatin markers; the protein localizes broadly along the length of the autosomes while being specifically excluded from the X chromosome, which is known to contain repressive chromatin modifications. Identification of SPE-44-interacting factors will be crucial in determining the mechanism(s) by which SPE-44 and other SAND-domain proteins regulate gene expression.

SPE-44 is not the sole transcriptional regulator that promotes sperm-specific gene expression. The list of *spe-44* targets comprises only a subset of the 1,343 sperm-enriched genes identified previously [22], [23], and a majority of Spe genes with demonstrated roles in spermatogenesis are expressed appropriately in *spe-44* animals. Ten additional transcriptional regulator homologs exhibit sperm-enriched expression, and those are potential mediators of sperm gene transcription. Although *elt-1* expression is governed by SPE-44, the other transcription factors are not and could promote sperm gene transcription independently of SPE-44. In addition to those candidates, transcription factors that also function in somatic tissues might not have been identified as sperm-enriched but could nonetheless promote sperm gene expression in the germline. Alternatively, the observed sperm-enriched expression of some genes could reflect repression during oogenesis, and negative regulators might be predicted to exhibit oocyte-enriched, rather than sperm-enriched, expression.

The conservation of *spe-44*, and its presumed role in sperm gene transcription, may be somewhat surprising given the rapid evolution of reproduction within the *Caenorhabditis* group of nematodes. Hermaphroditism has evolved independently at least twice from the ancestral male/female species [68], [69]. Differences in the germline sex determination programs between *C. elegans* and *C. briggsae* include gene loss, gene gain, and reordering of the pathway components [70]–[72]. Nonetheless, the data strongly suggest that *spe-44* function has been retained. The orthologous gene exhibits male-biased expression among the male/female species of the group. That expression likely reflects the conservation of sequence elements within the putative promoters of these orthologs. Most remarkable is the conserved pattern of SPE-44 protein localization in the male germline of *C. remanei*, including binding to the autosomes and exclusion from the presumptive X chromosome. Sperm genes are largely absent from the X chromosome in *C. elegans*, and chromatin markers indicate that X is transcriptionally silent at the time of sperm gene expression [22], [59]. Although the chromosomal distribution of sperm genes in nematodes other than *C. elegans* is currently unknown, germline silencing of the X chromosome is evolutionarily conserved among this group [59]. This observation strongly predicts a similar restriction of sperm genes to the autosomes, the site of SPE-44 binding, among these *Caenorhabditis* species. Taken together, the data are consistent with a conserved role for *spe-44* in sperm gene expression.

One unanswered question is how the expression of *spe-44* is itself regulated by the germline sex determination pathway. Our quantitative RT-PCR results clearly indicate that, in *C. elegans*, *spe-44* is governed by the terminal components FEM-1, FEM-3, and FOG-1 at the level of transcription and/or mRNA stability. The FEM proteins are part of a CUL-2-dependent E3 ubiquitin ligase complex, so one potential mechanism would be via degradation of a repressor of *spe-44* expression. That repressor is unlikely to be TRA-1, despite its demonstrated role as a target of FEM-dependent degradation and a repressor of male sexual fate in the soma. The FEM proteins specify gamete fate in the absence of TRA-1, and the *spe-44* promoter region does not contain sequences that match the known TRA-1 consensus binding site [73]. However, there are highly conserved sequence elements upstream of *spe-44* that might serve as binding sites for as-yet-unidentified regulatory proteins, which could be targets of FEM-dependent degradation. Similarly, FOG-1 is predicted to govern the translation of unknown factors that specify gamete fate, one or more of which might control transcription of *spe-44*. Alternatively, the putative polyadenylation-binding activity of FOG-1 might play a more direct role in regulating *spe-44* transcript stability.

The timing and specificity of *spe-44* expression indicate its critical role in implementing germline sexual fate. SPE-44 is one of the earliest known markers of sperm fate in *C. elegans*. Both temporally and spatially, SPE-44 precedes the production of other sperm-specific components. The precise, cell-specific pattern of SPE-44 localization during the spermatogenesis-to-oogenesis transition of hermaphrodites predicts that the transcription, translation, and protein stability of SPE-44 must be tightly regulated by a variety of feedback mechanisms. This exquisite restriction of SPE-44 to developing spermatocytes provides a powerful tool for the analysis of mutants that, due to defects in the germline sex determination program, produce gametes of sexually indeterminate character.

## Materials and Methods

*C. elegans* strains were obtained from the Caenorhabditis Genetics Center, and are derived from the wild-type isolate N2 (Bristol). The *spe-44(ok1400)* deletion allele was isolated by the Gene Knockout Consortium [43] and backcrossed six times to N2 prior to analysis. Additional mutations include: *dpy-20(e1282)IV, fem-1(hc17)IV, fem-3(q20gf)IV, fog-1(q253)I, let-92(s677)IV, unc-22(s7)IV*. A linked *spe-44(ok1400) dpy-20(e1282)* mutant strain, balanced by *let-92(s677) unc-22(s7)*, was generated to facilitate discrimination of homozygous lines. Homozygous *spe-44* males were obtained by mating balanced heterozygous males to homozygous Dpy Spe hermaphrodites and picking Dpy male progeny. Sodium hypochlorite treatment of gravid adults was used to obtain embryos for age-synchronized analyses. Strains were propagated on OP50-seeded NGM plates and maintained at 15°C unless otherwise indicated. Genetic manipulations were carried out by standard methods [74]. Self-fertility assays were performed by total progeny counts of individual hermaphrodites; cross-fertility was assessed by mating individual hermaphrodites with four males for 24 hours, then counting total progeny.

Single-worm PCR detection of the *spe-44(ok1400)* deletion utilized primers HES-343, HES-364, and HES-502 (all primers listed in Table S5). Microinjection rescue was performed with plasmid pHS584, a 6.5 kbp fragment of the *spe-44* genomic interval; included in the microinjection mix were plasmid pRF4, which contains the dominant *rol-6(su1006)* as a morphological marker, and genomic DNA, which enhances germline expression by formation of complex arrays [75], [76]. Rescue was determined by injecting balanced *spe-44(ok1400) dpy-20(e1282)/let-92(s677) unc-22(s7)* hermaphrodites, picking individual dumpy rollers from the F2 generation grown at 25°C, and counting total progeny. For qPCR, RNA was isolated via Trizol (Invitrogen) from triplicate samples (each ∼500 animals) grown at 25°C at the indicated developmental stage, reverse-transcribed with Superscript II (Invitrogen), and amplified with primers HES-502 and HES-539 (for *spe-44*) or AKA-70 and AKA-71 (for *act-1*). For yeast expression assays, the *spe-44* cDNA was amplified with primers HES-531 and HES-532 and inserted into a derivative of pJG4-5 [77] that lacks the transcriptional activation domain. Putative promoter fragments were amplified from genomic DNA with primers HES-612 and HES-613 (*spe-7*) or HES-583 and HES-584 (*spe-4*) and inserted into a derivative of pLacZi (Clontech); the same *spe-4* upstream sequence is sufficient to confer *in vivo* transgene rescue of *spe-4* sterility [78]. Yeast media and manipulations followed standard protocols [79].

Intact gonads were obtained by dissection of individual worms. Sperm spreads were obtained by further applying slight pressure to the coverslip. Antibody staining of dissected gonads followed established protocols [e.g., see 46]. Specimens were incubated with primary antibodies for 2–3 hours at room temperature unless otherwise indicated. Primary antibodies included: FITC-labeled anti-alpha-tubulin (DMIA, Sigma) (used at 1∶100 dilution), anti-MSP (4D5, gift from D. Greenstein) (1∶200), anti-phospho-histone H3 (serine10) (Upstate Biotechnology) (1∶150), anti-MPM2 (DAKO Corp.) (1∶100 overnight at 4°C), anti-histone H3 dimethylated Lys 9 (Upstate Biotechnology) (1∶50), 1CB4 [49]. The SPE-44 antibody (1∶100) was generated in rabbits against a C-terminal peptide epitope and affinity-purified (Open Biosystems). Affinity-purified secondary antibodies (Jackson Immunoresearch Laboratories) (1∶100) included goat anti-rabbit TRITC-labeled IgG and FITC- or DyLight-labeled goat anti-mouse IgG. Images were acquired under differential inference contrast or epifluorescence using an Olympus BX60 microscope equipped with a Cooke Sensicam cooled CCD camera and IPLab software. In some cases, images were minimally processed to enhance contrast either with IPLab software or Photoshop.

*In situ* hybridization for *spe-44* germ line expression was performed on dissected gonads following fixation [80]. Digoxigenin-labeled, single-stranded sense and antisense probes were generated from a 1.3 kb *spe-44* cDNA fragment by linear amplification according to the manufacturer's protocol (Roche). Following hybridization, probe detection was by colorimetric assay with alkaline phosphatase-conjugated anti-digoxigenin antibodies and NBT/BCIP substrate.

Worms for microarray screening were obtained by hand-picking samples of 50 L4 males of the indicated genotype. RNA was isolated by Trizol treatment and ethanol precipitation using linear polyacrylamide (GeneElute LPA; Sigma-Aldrich) as carrier. RNA was amplified and labeled according to the manufacturer's protocol (Nugen). Microarray screening was performed in triplicate using GeneChip *C. elegans* genome arrays (Affymetrix). Microarray data were analyzed by Microarray Suite 5.0 (Affymetrix) and Genomics Suite (Partek) software, using a p-value threshold of 0.05 for differential expression. Sequences for comparative analysis of *Caenorhabditis* species were obtained from WormBase release WS220 [www.wormbase.org]. Multiple sequence alignments of *spe-44* homologs and promoters were performed with CLUSTALW2 [81].

## Supporting Information

Figure S1 Spermatogenesis defect in *spe-44* hermaphrodites. To explore the nature of the sperm defect, spermathecae of young adult hermaphrodites (24–36 hours past the adult molt) were examined for the presence of sperm. In wild-type hermaphrodites, light microscopy revealed abundant motile spermatozoa within the spermathecae of intact adults (A, arrow). In contrast, the spermathecae of *spe-44* hermaphrodites were devoid of spermatozoa (B, arrowhead). The absence of sperm could reflect either a failure to produce sperm due to germ line feminization, or a defect in spermatogenesis that prevents the formation of motile spermatozoa. To distinguish between these two possibilities, we examined isolated gonads of *spe-44* hermaphrodites during the sperm/oocyte transition. Round immotile cells (sp), much smaller than oocytes (ooc), were observed in the region immediately proximal to the first oocyte; these cells appear identical to the aberrant spermatocytes observed in *spe-44* males (C; higher magnification in D). Therefore, *spe-44* germlines are not feminized but instead produce defective spermatocytes.
(TIF)

Figure S2 SPE-44 localization during male development. Dissected gonads from wild-type males at A) L2, B) early L3, and C) L4 developmental stages co-stained with DAPI (red) and anti-SPE-44 antibody (green).
(TIF)

Figure S3 Specificity of anti-SPE-44 antibody. A–B. Dissected gonads from wild-type (A) or *spe-44* (B) adult males co-stained with DAPI (red) and anti-SPE-44 antibody (green). Images were taken at identical exposures for comparison. C–D. Same images as A and B, respectively, showing anti-SPE-44 antibody alone.
(TIF)

Figure S4 SPE-44 localization in mid-L3 hermaphrodite. Dissected gonad co-stained with DAPI (red) and anti-SPE-44 antibody (green). SPE-44 is restricted to the pachytene region.
(TIF)

Table S1 Candidate sperm transcriptional regulators. Listed are genes with homology to known transcriptional regulators that exhibit sperm-enriched expression.
(DOC)

Table S2 Summary of microarray results. Listed are genes that exhibit greater than two-fold difference in expression between wild-type and *spe-44* L4 males with p-value<0.05. Fields (in order) are: WormBase Identifier; *spe-44*/wild-type expression ratio; p-value; and germline profile classification from Reinke et al. [23].
(DOC)

Table S3 GO terms associated with *spe-44* down-regulated genes. Listed are the genes from Table S2 with reduced expression in the *spe-44* mutant strain, and all of the Gene Ontology terms associated with those genes.
(DOC)

Table S4 GO terms associated with *spe-44* up-regulated genes. Listed are the genes from Table S2 with increased expression in the *spe-44* mutant strain, and all of the Gene Ontology terms associated with those genes.
(DOC)

Table S5 Oligonucleotide sequences. Listed are primers used for single-worm PCR, quantitative RT-PCR, and plasmid constructions.
(DOC)
