# Supplementary figures and images for: SPE-44 Implements Sperm Cell Fate
Source: PLoS Genet. 2012 Apr 26;8(4):e1002678. doi: 10.1371/journal.pgen.1002678 (PMC3343087; doi:10.1371/journal.pgen.1002678)

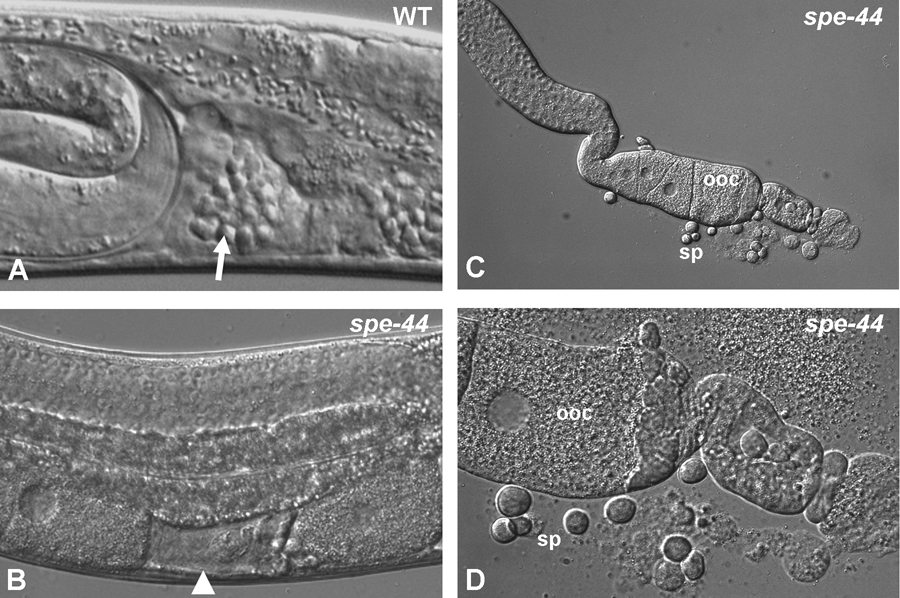

Supplement: Figure S1 — Spermatogenesis defect in spe-44 hermaphrodites. To explore the nature of the sperm defect, spermathecae of young adult hermaphrodites (24–36 hours past the adult molt) were examined for the presence of sperm. In wild-type hermaphrodites, light microscopy revealed abundant motile spermatozoa within the spermathecae of intact adults (A, arrow). In contrast, the spermathecae of spe-44 hermaphrodites were devoid of spermatozoa (B, arrowhead). The absence of sperm could reflect either a failure to produce sperm due to germ line feminization, or a defect in spermatogenesis that prevents the formation of motile spermatozoa. To distinguish between these two possibilities, we examined isolated gonads of spe-44 hermaphrodites during the sperm/oocyte transition. Round immotile cells (sp), much smaller than oocytes (ooc), were observed in the region immediately proximal to the first oocyte; these cells appear identical to the aberrant spermatocytes observed in spe-44 males (C; higher magnification in D). Therefore, spe-44 germlines are not feminized but instead produce defective spermatocytes. (TIF) [file pgen.1002678.s001.tif]

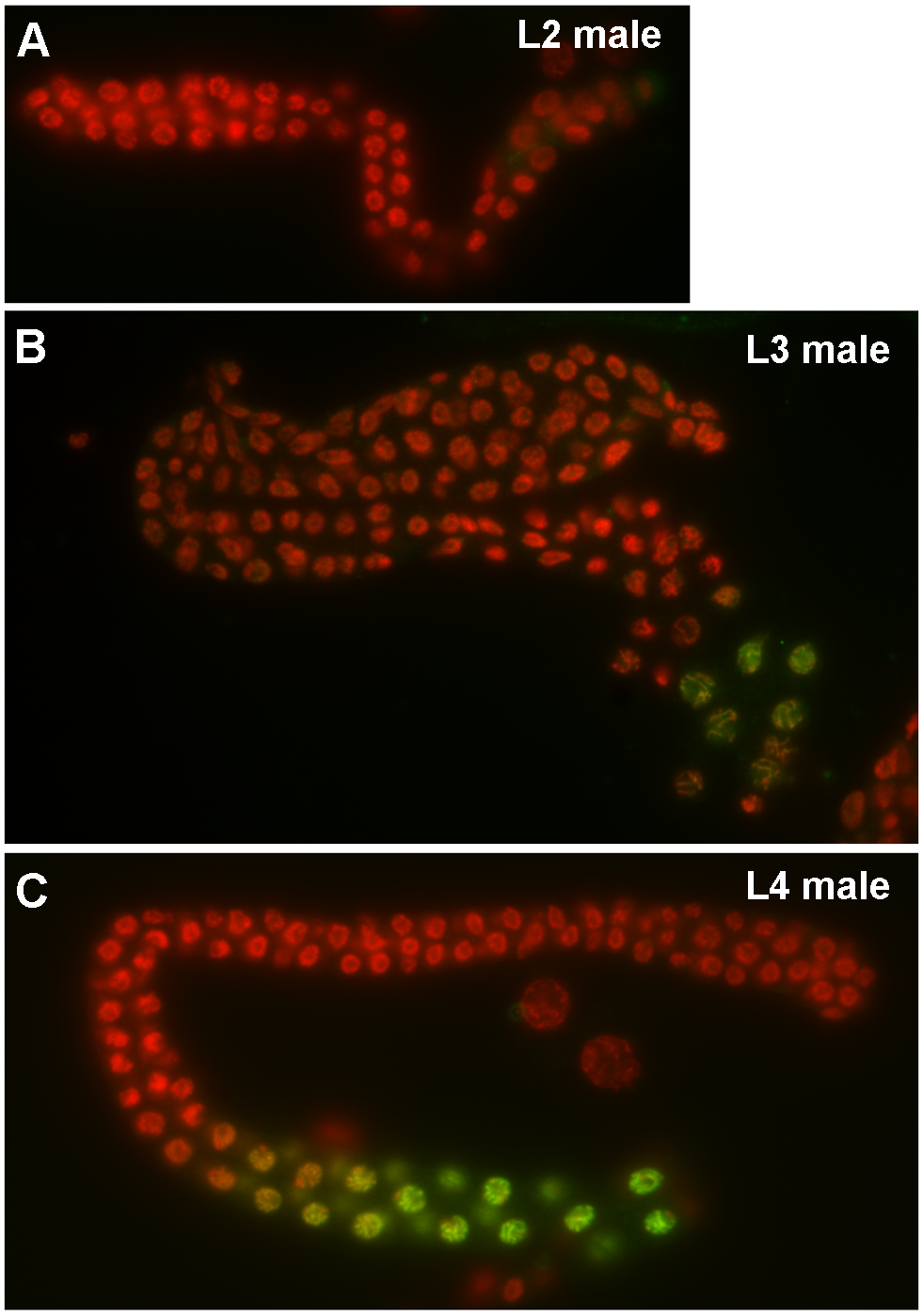

Supplement: Figure S2 — SPE-44 localization during male development. Dissected gonads from wild-type males at A) L2, B) early L3, and C) L4 developmental stages co-stained with DAPI (red) and anti-SPE-44 antibody (green). (TIF) [file pgen.1002678.s002.tif]

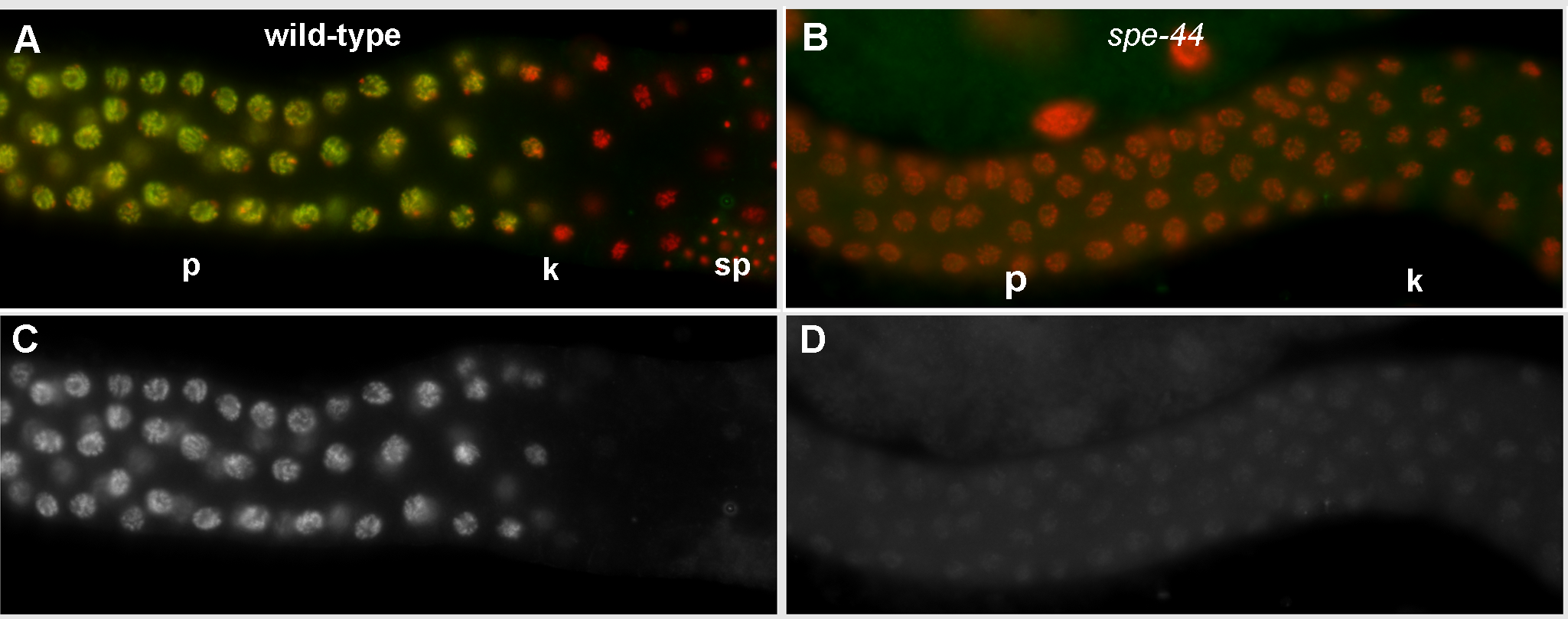

Supplement: Figure S3 — Specificity of anti-SPE-44 antibody. A–B. Dissected gonads from wild-type (A) or spe-44 (B) adult males co-stained with DAPI (red) and anti-SPE-44 antibody (green). Images were taken at identical exposures for comparison. C–D. Same images as A and B, respectively, showing anti-SPE-44 antibody alone. (TIF) [file pgen.1002678.s003.tif]

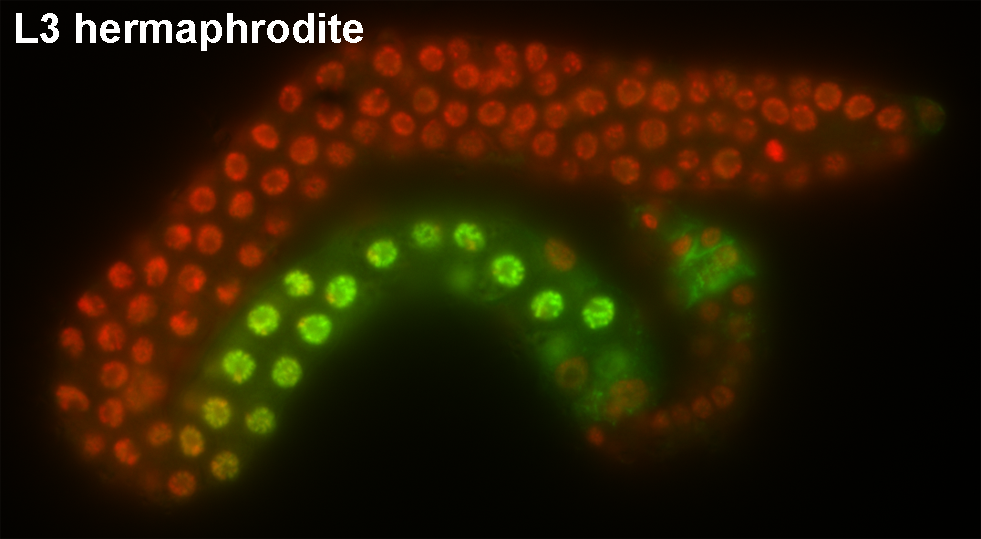

Supplement: Figure S4 — SPE-44 localization in mid-L3 hermaphrodite. Dissected gonad co-stained with DAPI (red) and anti-SPE-44 antibody (green). SPE-44 is restricted to the pachytene region. (TIF) [file pgen.1002678.s004.tif]
