# Supplementary material for: SPE-44 Implements Sperm Cell Fate
Source: PLoS Genet. 2012 Apr 26;8(4):e1002678. doi: 10.1371/journal.pgen.1002678 (PMC3343087; doi:10.1371/journal.pgen.1002678)
Supplement: Table S1 — Candidate sperm transcriptional regulators. Listed are genes with homology to known transcriptional regulators that exhibit sperm-enriched expression. (DOC) [file pgen.1002678.s005.doc]

| **Table S1. List of candidate sperm transcriptional regulators** | | |
| --- | --- | --- |
| **Gene** | **Sperm-enriched expression^a^** | **Functional class** |
| *attf-1* | 6.6 | SAND domain |
| *C25G4.4* | 2.1 | SAND domain |
| *ceh-48* | 6.6 | Homeodomain |
| *elt-1* | 2.5 | GATA factor |
| *F26F4.8* | 4.6 | *ovo/shb* homolog |
| *F43G6.6* | 13.8 | PHD domain |
| *htas-1* | 57.1 | Histone H2A variant |
| *his-70* | 22.7 | Histone H3 variant |
| *nhr-43* | 8.7 | Nuclear hormone receptor |
| *nhr-62* | 2.3 | Nuclear hormone receptor |
| *T20H4.2* | 15.9 | C2H2 zinc finger |
| Expression ratio of *fem-3(gf)/fem-1(lf)* adults; from Reinke et al., 2004. | | |
