# Supplementary material for: SPE-44 Implements Sperm Cell Fate
Source: PLoS Genet. 2012 Apr 26;8(4):e1002678. doi: 10.1371/journal.pgen.1002678 (PMC3343087; doi:10.1371/journal.pgen.1002678)
Supplement: Table S2 — Summary of microarray results. Listed are genes that exhibit greater than two-fold difference in expression between wild-type and spe-44 L4 males with p-value<0.05. Fields (in order) are: WormBase Identifier; spe-44/wild-type expression ratio; p-value; and germline profile classification from Reinke et al. [23]. (DOC) [file pgen.1002678.s006.doc]

| **Table S2. Microarray results for differentially expressed genes** | | | |
| --- | --- | --- | --- |
| **WormBase Identifier** | **spe-44/N2 ratio** | **p-value** | **germline class** |
| WBGene00015051 | -131.368 | 0.000526155 | shared sperm |
| WBGene00006056 | -103.649 | 0.000386138 | shared sperm |
| WBGene00014247 | -92.8397 | 0.00370385 | shared sperm |
| WBGene00013713 | -83.5952 | 0.00119041 | shared sperm |
| WBGene00013956 | -67.6136 | 0.000574509 | shared sperm |
| WBGene00011176 | -67.5573 | 0.000426456 | shared sperm |
| WBGene00017851 | -60.7094 | 0.000273513 | shared sperm |
| WBGene00044062 | -59.404 | 1.66E-05 | shared sperm |
| WBGene00007457 | -55.4972 | 0.000511737 | shared sperm |
| WBGene00018253 | -52.8289 | 0.000764469 | shared sperm |
| WBGene00007763 | -50.3724 | 0.000770751 | male sperm |
| WBGene00017815 | -48.6809 | 0.00108885 | shared sperm |
| WBGene00021880 | -45.0949 | 0.00151118 |  |
| WBGene00011478 | -39.3435 | 0.00163367 | herm sperm |
| WBGene00012087 | -37.4506 | 0.00303398 | shared sperm |
| WBGene00007987 | -37.0741 | 0.002775 | shared sperm |
| WBGene00022090 | -35.8174 | 0.000747376 | shared sperm |
| WBGene00015516 | -34.8722 | 0.00210937 | shared sperm |
| WBGene00044684 | -34.4539 | 0.00110126 | shared sperm |
| WBGene00021993 | -34.2815 | 0.000233363 | shared sperm |
| WBGene00007888 | -34.1713 | 0.00238183 | shared sperm |
| WBGene00018158 | -34.0049 | 0.000441485 | shared intrinsic |
| WBGene00021996 | -30.0199 | 0.00124692 | shared sperm |
| WBGene00007308 | -29.1946 | 0.00159971 | shared sperm |
| WBGene00016327 | -29.1896 | 0.000493285 | shared sperm |
| WBGene00022652 | -29.1539 | 0.000784427 | shared sperm |
| WBGene00021537 | -28.1239 | 0.0019266 |  |
| WBGene00009308 | -27.3584 | 0.00124224 | shared sperm |
| WBGene00018336 | -26.6038 | 0.000515182 |  |
| WBGene00018949 | -26.0932 | 0.000777001 |  |
| WBGene00016322 | -25.9481 | 0.00628284 | shared sperm |
| WBGene00011467 | -25.5497 | 0.00120731 | shared sperm |
| WBGene00023490 | -25.2213 | 0.000540216 |  |
| WBGene00007478 | -24.8056 | 0.00100747 | shared sperm |
| WBGene00016399 | -24.4584 | 0.000854178 | shared sperm |
| WBGene00001839 | -23.4876 | 0.000565632 |  |
| WBGene00017808 | -22.8624 | 0.00190904 | male sperm |
| WBGene00007948 | -22.7425 | 0.00692119 | male intrinsic |
| WBGene00022007 | -22.5566 | 0.000143227 |  |
| WBGene00044475 | -22.4946 | 0.000778484 |  |
| WBGene00016612 | -22.4704 | 0.00560903 | shared sperm |
| WBGene00016730 | -22.2108 | 0.00204637 | shared sperm |
| WBGene00011619 | -22.1124 | 0.00323969 |  |
| WBGene00009031 | -21.2019 | 0.00276747 | shared sperm |
| WBGene00016357 | -20.1909 | 0.0026435 |  |
| WBGene00022707 | -19.527 | 0.0016028 | shared sperm |
| WBGene00014238 | -18.9142 | 0.00288095 | shared sperm |
| WBGene00011466 | -18.4716 | 0.000971066 | shared sperm |
| WBGene00016336 | -18.3427 | 0.00215865 | male sperm |
| WBGene00018332 | -18.1972 | 0.00166501 | shared sperm |
| WBGene00018119 | -18.1961 | 0.00198677 | shared sperm |
| WBGene00017902 | -18.1642 | 0.00101233 | shared sperm |
| WBGene00004899 | -18.1242 | 0.00100197 |  |
| WBGene00006057 | -17.8787 | 0.000254795 | shared sperm |
| WBGene00015987 | -17.4984 | 0.00234447 | shared sperm |
| WBGene00001424 | -17.4099 | 0.000360208 | shared sperm |
| WBGene00012741 | -17.0504 | 0.0011398 | shared sperm |
| WBGene00019431 | -17.0005 | 0.00141996 | shared sperm |
| WBGene00017910 | -16.8302 | 0.0014741 | sperm |
| WBGene00017112 | -16.6712 | 0.00062184 | shared sperm |
| WBGene00016946 | -16.4988 | 0.00164935 | shared sperm |
| WBGene00019151 | -16.4193 | 0.000359616 |  |
| WBGene00013192 | -16.0655 | 0.00316161 | herm sperm |
| WBGene00007224 | -16.0168 | 0.00178719 | herm sperm |
| WBGene00019081 | -15.9697 | 0.00117525 | shared sperm |
| WBGene00016183 | -15.8195 | 0.00579346 | sperm |
| WBGene00006047 | -15.699 | 0.00110171 | shared sperm |
| WBGene00023424 | -15.5446 | 0.000553523 |  |
| WBGene00013710 | -15.0867 | 0.00264366 | shared sperm |
| WBGene00012177 | -14.9843 | 0.00120477 | shared sperm |
| WBGene00014229 | -14.859 | 0.000162185 |  |
| WBGene00013700 | -14.7663 | 0.000933355 | shared sperm |
| WBGene00008660 | -14.5148 | 0.00232776 | shared sperm |
| WBGene00016351 | -14.4707 | 0.00101561 |  |
| WBGene00007732 | -14.3786 | 0.0157085 | shared sperm |
| WBGene00006645 | -14.0047 | 0.000946352 | herm sperm |
| WBGene00011968 | -13.9579 | 0.00466524 |  |
| WBGene00011001 | -13.7864 | 7.61E-05 | sperm |
| WBGene00021016 | -13.7621 | 0.00062314 | herm sperm |
| WBGene00018087 | -13.74 | 0.000434458 | male sperm |
| WBGene00010763 | -13.3501 | 0.00248069 | herm sperm |
| WBGene00022632 | -13.3161 | 0.000743055 | shared sperm |
| WBGene00008313 | -13.0092 | 0.0032085 | shared sperm |
| WBGene00022709 | -12.7812 | 0.00134083 | shared sperm |
| WBGene00012855 | -12.6418 | 0.000290453 | shared sperm |
| WBGene00008662 | -12.6101 | 0.000647717 | male sperm |
| WBGene00007269 | -12.1387 | 0.00372714 | male intrinsic |
| WBGene00010623 | -12.1001 | 0.00229323 | shared sperm |
| WBGene00016707 | -11.8985 | 0.011858 | shared sperm |
| WBGene00006625 | -11.6238 | 0.00259032 | shared sperm |
| WBGene00017672 | -11.5439 | 0.00572066 | shared sperm |
| WBGene00015765 | -11.4542 | 0.0107917 | shared sperm |
| WBGene00007777 | -11.068 | 0.00115245 | shared sperm |
| WBGene00019260 | -10.8933 | 0.0034485 |  |
| WBGene00016698 | -10.7271 | 0.00145049 | shared sperm |
| WBGene00019950 | -10.6663 | 0.000951659 |  |
| WBGene00020713 | -10.4937 | 0.000435919 | shared sperm |
| WBGene00020370 | -10.4848 | 0.00328036 | male intrinsic |
| WBGene00016054 | -10.2384 | 0.000201789 | shared sperm |
| WBGene00010682 | -10.1068 | 0.00188715 | shared sperm |
| WBGene00009028 | -10.0841 | 0.00173072 | shared sperm |
| WBGene00007159 | -10.0522 | 0.0121153 | shared sperm |
| WBGene00020356 | -10.0409 | 0.0204074 |  |
| WBGene00007639 | -10.0398 | 0.00104285 | shared sperm |
| WBGene00007337 | -9.9968 | 0.00407588 | shared sperm |
| WBGene00004973 | -9.99076 | 0.00187011 | sperm |
| WBGene00012786 | -9.97483 | 0.000302323 | shared sperm |
| WBGene00020656 | -9.87779 | 0.000806439 | herm sperm |
| WBGene00017055 | -9.76121 | 0.00298473 |  |
| WBGene00020419 | -9.75209 | 0.0141646 | sperm |
| WBGene00015997 | -9.68651 | 0.010553 | shared sperm |
| WBGene00007354 | -9.60557 | 0.00761245 | shared sperm |
| WBGene00018168 | -9.58013 | 0.00345273 | shared sperm |
| WBGene00022143 | -9.56199 | 0.00259534 |  |
| WBGene00010046 | -9.48782 | 0.00520218 | shared sperm |
| WBGene00007756 | -9.42232 | 0.0037588 | sperm |
| WBGene00005013 | -9.3663 | 0.00231198 | male sperm |
| WBGene00010829 | -9.24855 | 0.00268422 | shared sperm |
| WBGene00009446 | -9.20971 | 0.000820034 | shared sperm |
| WBGene00018359 | -9.18519 | 0.00344498 | shared sperm |
| WBGene00013165 | -9.15627 | 0.00222631 |  |
| WBGene00016942 | -9.12191 | 0.005298 | shared sperm |
| WBGene00000248 | -9.04898 | 0.00277391 | sperm |
| WBGene00014157 | -8.85177 | 0.0016544 | shared sperm |
| WBGene00020380 | -8.7673 | 0.0104723 |  |
| WBGene00013033 | -8.72356 | 0.00591946 | shared sperm |
| WBGene00012784 | -8.50543 | 0.00424307 | herm sperm |
| WBGene00004908 | -8.47249 | 0.0124681 |  |
| WBGene00021030 | -8.33441 | 0.00117575 | sperm |
| WBGene00015094 | -8.25115 | 0.00199589 | shared sperm |
| WBGene00011295 | -8.21587 | 0.00257971 |  |
| WBGene00009646 | -8.12819 | 0.006332 | herm sperm |
| WBGene00008590 | -8.12563 | 0.000426506 | shared sperm |
| WBGene00010883 | -7.91816 | 0.00055777 | shared sperm |
| WBGene00013303 | -7.87303 | 0.00237235 | sperm |
| WBGene00006048 | -7.84404 | 0.000309099 | shared sperm |
| WBGene00006719 | -7.72584 | 0.00129259 | shared sperm |
| WBGene00011008 | -7.68322 | 0.00606397 | herm sperm |
| WBGene00019015 | -7.67939 | 0.00641733 | shared sperm |
| WBGene00009948 | -7.60591 | 5.68E-05 | shared sperm |
| WBGene00013158 | -7.5965 | 0.00490707 | shared sperm |
| WBGene00020372 | -7.51493 | 0.000291528 | shared sperm |
| WBGene00013701 | -7.46573 | 0.00144112 | sperm |
| WBGene00008383 | -7.44474 | 0.00616068 | shared sperm |
| WBGene00016753 | -7.36432 | 0.000826777 | herm sperm |
| WBGene00015004 | -7.35289 | 0.00104585 | sperm |
| WBGene00022752 | -7.3465 | 0.0010192 | herm sperm |
| WBGene00022102 | -7.33914 | 0.00372776 |  |
| WBGene00008581 | -7.21878 | 0.0021293 | shared sperm |
| WBGene00012642 | -7.21085 | 0.00398688 |  |
| WBGene00007382 | -7.20654 | 0.00990053 |  |
| WBGene00009755 | -7.19337 | 0.0017315 | shared sperm |
| WBGene00013478 | -7.19011 | 0.00146586 | shared sperm |
| WBGene00009042 | -7.17873 | 0.000144815 | sperm |
| WBGene00009592 | -7.16122 | 0.00548758 | shared sperm |
| WBGene00020435 | -7.07126 | 0.00309769 | shared sperm |
| WBGene00002208 | -7.00048 | 0.000895814 | shared sperm |
| WBGene00015026 | -6.99389 | 0.0130941 | herm sperm |
| WBGene00011191 | -6.93767 | 0.0139274 | shared sperm |
| WBGene00010634 | -6.93161 | 0.0027003 | herm sperm |
| WBGene00013087 | -6.92438 | 0.000589593 | shared sperm |
| WBGene00018301 | -6.92309 | 0.00216859 | shared sperm |
| WBGene00008137 | -6.86985 | 0.0135593 | shared sperm |
| WBGene00015193 | -6.86862 | 0.0218339 | shared sperm |
| WBGene00015937 | -6.81793 | 0.000265573 | shared sperm |
| WBGene00020350 | -6.80735 | 0.00363975 | shared sperm |
| WBGene00015661 | -6.80014 | 0.00291 | shared sperm |
| WBGene00008333 | -6.73859 | 0.0123493 | sperm |
| WBGene00008433 | -6.72787 | 0.00152599 | shared intrinsic |
| WBGene00001249 | -6.64459 | 0.00489688 | sperm |
| WBGene00012849 | -6.6131 | 0.00423844 | male sperm |
| WBGene00010611 | -6.56973 | 0.000432818 | shared sperm |
| WBGene00020433 | -6.47381 | 0.0154982 | herm intrinsic |
| WBGene00003438 | -6.42015 | 0.00101331 | shared sperm |
| WBGene00017114 | -6.4121 | 0.00990921 | shared sperm |
| WBGene00012627 | -6.38116 | 0.0206964 | male sperm |
| WBGene00014239 | -6.35958 | 0.00912036 | shared sperm |
| WBGene00012636 | -6.31406 | 0.000953446 | male sperm |
| WBGene00012831 | -6.20839 | 0.0169584 | shared sperm |
| WBGene00010254 | -6.19344 | 0.00821811 | shared sperm |
| WBGene00021428 | -6.17772 | 0.000844788 | male intrinsic |
| WBGene00013521 | -6.16517 | 0.00205545 | shared sperm |
| WBGene00017679 | -6.13838 | 0.00109698 | male sperm |
| WBGene00018165 | -6.09354 | 0.00355984 | sperm |
| WBGene00022385 | -6.04046 | 0.00591324 | sperm |
| WBGene00013951 | -6.03694 | 0.00867935 | male sperm |
| WBGene00007243 | -6.00713 | 0.0101653 |  |
| WBGene00022699 | -5.99289 | 0.00217625 | shared sperm |
| WBGene00009125 | -5.93699 | 0.00579198 | male sperm |
| WBGene00016742 | -5.9339 | 0.00211939 | shared sperm |
| WBGene00003443 | -5.92461 | 9.53E-05 | shared sperm |
| WBGene00003442 | -5.90441 | 7.45E-05 | shared sperm |
| WBGene00019530 | -5.85859 | 0.00369619 | shared sperm |
| WBGene00022833 | -5.8386 | 0.00599838 |  |
| WBGene00021630 | -5.82303 | 0.00958369 | herm sperm |
| WBGene00012873 | -5.78358 | 0.00855034 | sperm |
| WBGene00008487 | -5.76267 | 0.00869509 | male sperm |
| WBGene00011911 | -5.7155 | 0.00628389 | shared sperm |
| WBGene00019812 | -5.70864 | 0.0062147 | shared sperm |
| WBGene00019461 | -5.66481 | 0.000862577 | sperm |
| WBGene00015049 | -5.6244 | 0.0103237 | shared intrinsic |
| WBGene00012297 | -5.62264 | 0.00363725 | sperm |
| WBGene00022780 | -5.52333 | 0.000688806 | shared sperm |
| WBGene00022003 | -5.49879 | 0.00861095 |  |
| WBGene00007274 | -5.47986 | 0.00176845 | herm sperm |
| WBGene00019175 | -5.45453 | 0.000670858 | shared sperm |
| WBGene00011134 | -5.44932 | 0.00117702 |  |
| WBGene00022617 | -5.42921 | 0.00413668 | male sperm |
| WBGene00009043 | -5.33494 | 0.00511792 | shared sperm |
| WBGene00007721 | -5.19043 | 0.00729535 | shared sperm |
| WBGene00006051 | -5.10587 | 0.00728992 | shared sperm |
| WBGene00019879 | -5.09329 | 0.000367834 | shared sperm |
| WBGene00015629 | -5.09023 | 0.00296547 | shared sperm |
| WBGene00010779 | -5.08822 | 0.00121853 | shared sperm |
| WBGene00012012 | -5.0297 | 0.00226835 | shared sperm |
| WBGene00020019 | -5.01315 | 4.21E-05 |  |
| WBGene00019425 | -4.99153 | 0.000761291 |  |
| WBGene00018068 | -4.96888 | 0.00174173 | male sperm |
| WBGene00011483 | -4.95394 | 0.00977972 |  |
| WBGene00019062 | -4.94589 | 0.0126629 | shared sperm |
| WBGene00009039 | -4.94031 | 0.0106267 | shared intrinsic |
| WBGene00012519 | -4.93136 | 0.0240584 | male intrinsic |
| WBGene00010915 | -4.90391 | 0.0191124 | herm sperm |
| WBGene00011777 | -4.83354 | 0.00126888 | shared sperm |
| WBGene00018773 | -4.81438 | 0.00245285 | herm sperm |
| WBGene00010265 | -4.8137 | 0.0226728 | shared sperm |
| WBGene00022467 | -4.80825 | 0.000624053 |  |
| WBGene00017802 | -4.80691 | 0.00497951 | shared sperm |
| WBGene00008854 | -4.73846 | 0.00245578 | herm sperm |
| WBGene00007825 | -4.71801 | 0.00179469 |  |
| WBGene00009005 | -4.70736 | 0.00347777 | sperm |
| WBGene00007610 | -4.68549 | 0.00330478 | shared sperm |
| WBGene00018001 | -4.6589 | 0.000670596 | shared sperm |
| WBGene00015093 | -4.63194 | 0.0183876 |  |
| WBGene00011129 | -4.55489 | 0.0216562 |  |
| WBGene00011501 | -4.55373 | 0.0043603 | herm oocyte |
| WBGene00020913 | -4.53718 | 0.00534102 | sperm |
| WBGene00012379 | -4.53312 | 0.0045243 | shared sperm |
| WBGene00012486 | -4.51942 | 0.00648819 | shared sperm |
| WBGene00050896 | -4.51345 | 0.00202663 |  |
| WBGene00010636 | -4.50853 | 0.00734971 | shared sperm |
| WBGene00019717 | -4.49052 | 0.00265261 |  |
| WBGene00020898 | -4.48271 | 0.0042619 | shared intrinsic |
| WBGene00013089 | -4.44705 | 0.0086722 |  |
| WBGene00020381 | -4.44057 | 0.00505232 | shared sperm |
| WBGene00011290 | -4.42237 | 0.00349952 | shared sperm |
| WBGene00013290 | -4.37249 | 0.0444498 | shared sperm |
| WBGene00013085 | -4.36959 | 0.00256061 | male sperm |
| WBGene00013987 | -4.36225 | 0.00483721 | shared sperm |
| WBGene00013318 | -4.33408 | 0.00213479 | male sperm |
| WBGene00013374 | -4.32658 | 0.00595108 | shared intrinsic |
| WBGene00015422 | -4.32582 | 0.020605 | shared sperm |
| WBGene00009491 | -4.31591 | 0.000626947 | male sperm |
| WBGene00003055 | -4.31081 | 0.0014465 |  |
| WBGene00007198 | -4.29914 | 0.0124336 | herm sperm |
| WBGene00015215 | -4.26568 | 0.00312604 | shared sperm |
| WBGene00013723 | -4.23091 | 0.00325217 | shared sperm |
| WBGene00010599 | -4.22892 | 0.0183162 |  |
| WBGene00016246 | -4.22324 | 0.00148831 | sperm |
| WBGene00006718 | -4.18595 | 0.0100981 |  |
| WBGene00010900 | -4.17094 | 0.00223819 | shared sperm |
| WBGene00021871 | -4.13708 | 0.0170458 |  |
| WBGene00020353 | -4.11265 | 0.0064333 | shared sperm |
| WBGene00003431 | -4.09991 | 0.000298664 | shared sperm |
| WBGene00007549 | -4.062 | 0.000131274 | sperm |
| WBGene00015032 | -4.05672 | 0.00587583 | male sperm |
| WBGene00018631 | -4.01822 | 0.00699596 |  |
| WBGene00007548 | -4.01757 | 0.0125201 | sperm |
| WBGene00012905 | -4.0148 | 0.00971959 | shared sperm |
| WBGene00013198 | -3.99475 | 0.0122358 |  |
| WBGene00016491 | -3.97662 | 0.00155322 | herm sperm |
| WBGene00013696 | -3.97145 | 0.00428657 | shared sperm |
| WBGene00022630 | -3.96862 | 0.0357831 | shared sperm |
| WBGene00020416 | -3.90639 | 0.00410778 | shared sperm |
| WBGene00019024 | -3.89302 | 0.0022973 | shared sperm |
| WBGene00006487 | -3.88142 | 0.030026 |  |
| WBGene00021298 | -3.87854 | 0.0089378 |  |
| WBGene00018098 | -3.87539 | 0.0226686 |  |
| WBGene00013712 | -3.85499 | 0.00652867 | male sperm |
| WBGene00019026 | -3.84225 | 0.000738687 |  |
| WBGene00008529 | -3.82503 | 0.0162585 | shared sperm |
| WBGene00018065 | -3.82308 | 0.0209883 |  |
| WBGene00005012 | -3.79668 | 0.00066525 | shared sperm |
| WBGene00010366 | -3.77307 | 0.00455972 | shared sperm |
| WBGene00009589 | -3.75972 | 0.000269059 |  |
| WBGene00018347 | -3.75665 | 0.000855983 | shared sperm |
| WBGene00003430 | -3.72695 | 0.000314709 | shared sperm |
| WBGene00017703 | -3.72593 | 0.0326726 | sperm |
| WBGene00011951 | -3.70426 | 0.0344356 | shared sperm |
| WBGene00021457 | -3.70082 | 0.0199537 | sperm |
| WBGene00003457 | -3.69385 | 0.000693699 | shared sperm |
| WBGene00021608 | -3.68541 | 0.0192309 |  |
| WBGene00012787 | -3.68389 | 0.0130237 | sperm |
| WBGene00009016 | -3.67402 | 0.0157904 | shared sperm |
| WBGene00022440 | -3.66252 | 0.0091721 |  |
| WBGene00020992 | -3.64259 | 0.0398622 |  |
| WBGene00021786 | -3.61728 | 0.010684 |  |
| WBGene00018999 | -3.58496 | 0.00832547 | shared sperm |
| WBGene00015034 | -3.58254 | 0.00208051 |  |
| WBGene00012656 | -3.56017 | 0.0482931 | male sperm |
| WBGene00016950 | -3.54953 | 0.0224145 | sperm |
| WBGene00010612 | -3.53572 | 0.0203457 |  |
| WBGene00010719 | -3.53405 | 0.015029 | shared sperm |
| WBGene00004949 | -3.52197 | 0.0113964 |  |
| WBGene00008623 | -3.51733 | 0.0107164 | male intrinsic |
| WBGene00021215 | -3.50938 | 0.0189977 |  |
| WBGene00045484 | -3.50416 | 0.00919884 |  |
| WBGene00014005 | -3.50361 | 0.0395047 | shared sperm |
| WBGene00022108 | -3.49876 | 0.011588 |  |
| WBGene00019135 | -3.49572 | 0.000238678 |  |
| WBGene00004904 | -3.49191 | 0.0307758 | shared sperm |
| WBGene00002120 | -3.46806 | 0.00573695 | shared sperm |
| WBGene00011669 | -3.46297 | 0.00771332 | shared sperm |
| WBGene00016083 | -3.46095 | 0.00707048 | shared sperm |
| WBGene00016511 | -3.45638 | 0.00749334 | sperm |
| WBGene00010487 | -3.44943 | 0.00355947 | male intrinsic |
| WBGene00003456 | -3.41096 | 0.000127403 | shared sperm |
| WBGene00007383 | -3.40157 | 0.00316309 | male sperm |
| WBGene00017533 | -3.38885 | 0.0266755 | shared sperm |
| WBGene00020973 | -3.38061 | 0.0320769 |  |
| WBGene00003470 | -3.37188 | 0.00238037 | shared sperm |
| WBGene00020940 | -3.36485 | 0.0126868 |  |
| WBGene00007335 | -3.36095 | 0.00827534 | shared sperm |
| WBGene00012925 | -3.36049 | 0.012681 |  |
| WBGene00009927 | -3.34533 | 0.00580317 |  |
| WBGene00019531 | -3.34518 | 0.0434398 | shared sperm |
| WBGene00008332 | -3.31517 | 0.00076758 |  |
| WBGene00016587 | -3.31238 | 0.00159263 |  |
| WBGene00009324 | -3.3102 | 0.0220463 | herm sperm |
| WBGene00004964 | -3.30564 | 0.0483051 |  |
| WBGene00020066 | -3.28083 | 0.0114354 | male sperm |
| WBGene00017553 | -3.26632 | 0.0162412 | shared sperm |
| WBGene00007740 | -3.25177 | 0.000750531 | shared sperm |
| WBGene00021908 | -3.21216 | 0.00339124 | male intrinsic |
| WBGene00019405 | -3.2056 | 0.000443381 | sperm |
| WBGene00015729 | -3.19088 | 0.00492945 |  |
| WBGene00009054 | -3.19073 | 0.000410276 | shared sperm |
| WBGene00020904 | -3.1729 | 0.0156199 | sperm |
| WBGene00022760 | -3.16847 | 0.000447671 | shared sperm |
| WBGene00011910 | -3.15491 | 0.00688188 | shared sperm |
| WBGene00022008 | -3.13666 | 0.00411635 |  |
| WBGene00003424 | -3.09339 | 0.000317524 | shared sperm |
| WBGene00012877 | -3.08271 | 0.0211872 |  |
| WBGene00019084 | -3.07978 | 0.00375479 | herm sperm |
| WBGene00018760 | -3.06318 | 0.00230071 |  |
| WBGene00009708 | -3.02353 | 0.0367803 | male sperm |
| WBGene00022243 | -3.01829 | 0.00019437 |  |
| WBGene00009492 | -3.01123 | 0.0309358 | shared sperm |
| WBGene00011173 | -3.00839 | 0.00624755 | shared sperm |
| WBGene00016320 | -3.00824 | 0.00861407 | sperm |
| WBGene00012827 | -3.00658 | 0.00784111 | shared sperm |
| WBGene00014793 | -2.99922 | 0.0394917 |  |
| WBGene00011053 | -2.99581 | 0.00724597 |  |
| WBGene00009149 | -2.97927 | 0.0372268 | shared sperm |
| WBGene00020985 | -2.96937 | 0.00100317 | sperm |
| WBGene00009195 | -2.95307 | 0.0055999 |  |
| WBGene00016058 | -2.93953 | 0.000174753 | shared sperm |
| WBGene00006997 | -2.93886 | 0.0182489 |  |
| WBGene00007148 | -2.93565 | 0.00514743 | male sperm |
| WBGene00012010 | -2.93201 | 0.00479455 | male sperm |
| WBGene00017636 | -2.92818 | 0.00111105 | shared sperm |
| WBGene00008725 | -2.92264 | 0.0272461 | male sperm |
| WBGene00015909 | -2.91697 | 0.0103528 |  |
| WBGene00019949 | -2.91437 | 0.0193641 | shared sperm |
| WBGene00009470 | -2.89608 | 0.0347405 | shared sperm |
| WBGene00019834 | -2.87537 | 0.0324421 | herm sperm |
| WBGene00011088 | -2.85979 | 0.0168925 | sperm |
| WBGene00011174 | -2.85252 | 0.0485859 | shared sperm |
| WBGene00010574 | -2.84775 | 0.00379318 |  |
| WBGene00008802 | -2.83498 | 0.00934011 | male sperm |
| WBGene00016075 | -2.8313 | 0.0174096 |  |
| WBGene00013461 | -2.81901 | 0.0025047 |  |
| WBGene00010596 | -2.81444 | 0.0444926 | herm sperm |
| WBGene00007794 | -2.8031 | 0.00183916 | shared sperm |
| WBGene00006624 | -2.7983 | 0.0159017 | male sperm |
| WBGene00011789 | -2.79422 | 0.0483885 | herm sperm |
| WBGene00010460 | -2.78539 | 0.00706098 | herm sperm |
| WBGene00020052 | -2.78341 | 0.00798268 | sperm |
| WBGene00010181 | -2.75982 | 0.00271496 | shared sperm |
| WBGene00018498 | -2.74785 | 0.00311707 | sperm |
| WBGene00003462 | -2.74553 | 0.000132783 | shared sperm |
| WBGene00022679 | -2.74114 | 0.0435796 |  |
| WBGene00008124 | -2.722 | 0.00146472 | shared sperm |
| WBGene00021151 | -2.70734 | 0.0400981 | shared sperm |
| WBGene00018121 | -2.69913 | 0.0378962 | shared sperm |
| WBGene00194816 | -2.69704 | 0.0494457 |  |
| WBGene00017654 | -2.68878 | 0.0327484 |  |
| WBGene00013999 | -2.68741 | 0.012001 | shared sperm |
| WBGene00011533 | -2.67684 | 0.0101582 | shared sperm |
| WBGene00013890 | -2.67665 | 0.0205441 | shared sperm |
| WBGene00009549 | -2.67554 | 0.0319707 | herm sperm |
| WBGene00015627 | -2.67279 | 0.0129382 | shared sperm |
| WBGene00019080 | -2.65912 | 0.00719081 | male sperm |
| WBGene00013773 | -2.65562 | 0.0420988 | male intrinsic |
| WBGene00017279 | -2.6553 | 0.0117052 |  |
| WBGene00007666 | -2.63628 | 0.0206957 |  |
| WBGene00019255 | -2.62448 | 0.00392575 | herm sperm |
| WBGene00017700 | -2.62298 | 0.00353908 | sperm |
| WBGene00008266 | -2.61686 | 0.0228243 |  |
| WBGene00017156 | -2.61401 | 0.00657889 |  |
| WBGene00010184 | -2.60985 | 0.00878531 | sperm |
| WBGene00010538 | -2.59182 | 0.00154196 | sperm |
| WBGene00017050 | -2.58551 | 0.0348635 | shared sperm |
| WBGene00022763 | -2.58058 | 0.0366843 | shared sperm |
| WBGene00043187 | -2.57531 | 0.00146507 | shared sperm |
| WBGene00014129 | -2.56945 | 0.0382057 |  |
| WBGene00007633 | -2.56435 | 0.0404172 | herm sperm |
| WBGene00009513 | -2.56299 | 0.04899 | shared sperm |
| WBGene00013888 | -2.54077 | 0.00415937 | shared sperm |
| WBGene00020881 | -2.53892 | 0.0321649 |  |
| WBGene00007300 | -2.53852 | 0.0234694 |  |
| WBGene00006704 | -2.53557 | 0.00405256 | herm intrinsic |
| WBGene00007567 | -2.532 | 0.0371722 |  |
| WBGene00016825 | -2.52388 | 0.0236584 | male sperm |
| WBGene00016613 | -2.52251 | 0.0193176 | sperm |
| WBGene00017501 | -2.50881 | 0.0458183 |  |
| WBGene00017195 | -2.50404 | 0.0259593 | sperm |
| WBGene00009782 | -2.50225 | 0.0193709 | male sperm |
| WBGene00009030 | -2.4915 | 0.00161698 |  |
| WBGene00007440 | -2.49084 | 0.046608 |  |
| WBGene00016912 | -2.48684 | 0.0154325 |  |
| WBGene00004739 | -2.46057 | 0.00614848 |  |
| WBGene00000222 | -2.45912 | 0.0327331 |  |
| WBGene00001185 | -2.45623 | 0.00718376 |  |
| WBGene00020468 | -2.45576 | 0.0249369 | shared sperm |
| WBGene00007571 | -2.45525 | 0.0361764 | herm intrinsic |
| WBGene00013175 | -2.44766 | 0.0257993 | shared sperm |
| WBGene00018004 | -2.44729 | 0.00146574 |  |
| WBGene00013458 | -2.4418 | 0.0303311 |  |
| WBGene00009550 | -2.43403 | 0.0344526 | shared sperm |
| WBGene00009457 | -2.42898 | 0.00752405 | shared sperm |
| WBGene00021294 | -2.41279 | 0.0137924 |  |
| WBGene00000865 | -2.41006 | 0.000961069 | herm intrinsic |
| WBGene00043147 | -2.40615 | 0.00024894 | shared sperm |
| WBGene00006039 | -2.40201 | 0.000870364 |  |
| WBGene00008724 | -2.40043 | 0.0132404 | shared sperm |
| WBGene00017853 | -2.3987 | 0.0143132 |  |
| WBGene00020550 | -2.39637 | 0.00111109 |  |
| WBGene00008651 | -2.36823 | 0.0348168 | sperm |
| WBGene00019302 | -2.36194 | 0.0333829 |  |
| WBGene00012118 | -2.35969 | 0.0353793 |  |
| WBGene00015246 | -2.35151 | 0.0277394 |  |
| WBGene00014240 | -2.34939 | 0.010266 | shared sperm |
| WBGene00017810 | -2.34188 | 0.0124819 |  |
| WBGene00012233 | -2.34129 | 0.00277785 |  |
| WBGene00015678 | -2.34004 | 0.00338676 |  |
| WBGene00015033 | -2.33843 | 0.0208317 |  |
| WBGene00003452 | -2.3348 | 7.09E-05 | shared sperm |
| WBGene00003435 | -2.33318 | 0.00104848 | shared sperm |
| WBGene00020044 | -2.33204 | 0.000742247 |  |
| WBGene00003429 | -2.32866 | 9.84E-05 | shared sperm |
| WBGene00008606 | -2.32178 | 0.015536 |  |
| WBGene00016562 | -2.32161 | 0.0130645 |  |
| WBGene00010014 | -2.32131 | 0.0490416 | sperm |
| WBGene00003185 | -2.3181 | 0.0172384 | oocyte |
| WBGene00010056 | -2.31738 | 0.000737453 | herm intrinsic |
| WBGene00014116 | -2.30812 | 0.0193282 | shared sperm |
| WBGene00008125 | -2.29238 | 0.0212685 | shared sperm |
| WBGene00013357 | -2.28044 | 0.0113503 |  |
| WBGene00009469 | -2.27488 | 0.00919992 |  |
| WBGene00000584 | -2.26973 | 0.0428876 |  |
| WBGene00003426 | -2.25681 | 7.09E-05 | shared sperm |
| WBGene00016288 | -2.25492 | 0.0125401 | shared sperm |
| WBGene00015386 | -2.25289 | 0.0168779 | male sperm |
| WBGene00020248 | -2.2521 | 0.0115644 |  |
| WBGene00001709 | -2.25162 | 0.0146313 |  |
| WBGene00016738 | -2.25105 | 0.0380122 |  |
| WBGene00019623 | -2.24471 | 0.00992282 |  |
| WBGene00014042 | -2.24111 | 0.00675885 | herm sperm |
| WBGene00011405 | -2.23946 | 0.0348406 | shared sperm |
| WBGene00002052 | -2.23592 | 0.0296178 | sperm |
| WBGene00017139 | -2.2348 | 0.00333544 |  |
| WBGene00012811 | -2.23186 | 0.0325652 |  |
| WBGene00003468 | -2.22367 | 8.05E-05 | shared sperm |
| WBGene00050879 | -2.21352 | 0.0148358 |  |
| WBGene00020737 | -2.21333 | 0.016034 |  |
| WBGene00021289 | -2.2106 | 0.0324575 |  |
| WBGene00018083 | -2.21058 | 0.00454974 | shared sperm |
| WBGene00007748 | -2.21057 | 0.0239395 |  |
| WBGene00013547 | -2.209 | 0.0365961 |  |
| WBGene00021761 | -2.20623 | 0.00145056 |  |
| WBGene00020508 | -2.20156 | 0.0420225 |  |
| WBGene00013473 | -2.19623 | 0.0113709 | shared sperm |
| WBGene00004971 | -2.19598 | 0.0216596 | shared sperm |
| WBGene00020580 | -2.19199 | 0.0245686 | sperm |
| WBGene00007574 | -2.18833 | 0.0178614 | sperm |
| WBGene00009442 | -2.18181 | 0.000328417 | herm intrinsic |
| WBGene00006895 | -2.18153 | 0.0470564 |  |
| WBGene00010743 | -2.17769 | 0.00983002 | sperm |
| WBGene00022884 | -2.17377 | 0.00201562 | shared sperm |
| WBGene00001638 | -2.1672 | 0.00488931 |  |
| WBGene00003469 | -2.16431 | 0.000332243 | shared sperm |
| WBGene00044989 | -2.16414 | 0.0235553 |  |
| WBGene00022002 | -2.16002 | 0.000528778 | shared sperm |
| WBGene00018410 | -2.15533 | 0.0430049 |  |
| WBGene00000383 | -2.1518 | 0.0373288 | oocyte |
| WBGene00009956 | -2.15028 | 0.0375097 | shared sperm |
| WBGene00013011 | -2.1468 | 0.0177843 |  |
| WBGene00009238 | -2.14678 | 0.0458998 | shared sperm |
| WBGene00005019 | -2.14372 | 0.0249199 | herm intrinsic |
| WBGene00011139 | -2.14315 | 0.0147069 |  |
| WBGene00001178 | -2.13819 | 0.0468811 |  |
| WBGene00011114 | -2.13736 | 0.0411188 |  |
| WBGene00019513 | -2.13241 | 0.0135085 | oocyte |
| WBGene00010859 | -2.13183 | 0.0389745 |  |
| WBGene00011203 | -2.1315 | 0.0471575 | shared sperm |
| WBGene00003444 | -2.12545 | 3.90E-05 | shared sperm |
| WBGene00013524 | -2.12524 | 0.0342595 | shared sperm |
| WBGene00003936 | -2.11963 | 0.000911756 |  |
| WBGene00012339 | -2.11916 | 0.0105396 |  |
| WBGene00013522 | -2.11911 | 0.0326665 |  |
| WBGene00006661 | -2.11085 | 0.0285719 | male intrinsic |
| WBGene00012295 | -2.10249 | 0.0292142 |  |
| WBGene00015084 | -2.10108 | 0.0256927 | shared intrinsic |
| WBGene00009548 | -2.10013 | 0.00500617 | herm sperm |
| WBGene00012408 | -2.09653 | 0.0202168 |  |
| WBGene00008629 | -2.08415 | 0.0263085 |  |
| WBGene00001045 | -2.07433 | 0.0131406 |  |
| WBGene00018122 | -2.06807 | 0.0103143 | shared sperm |
| WBGene00015934 | -2.06732 | 0.0104466 | sperm |
| WBGene00007916 | -2.05599 | 0.0464766 |  |
| WBGene00009465 | -2.05349 | 0.0480783 | shared intrinsic |
| WBGene00020400 | -2.04436 | 0.0234887 |  |
| WBGene00013013 | -2.03015 | 0.0267439 |  |
| WBGene00012122 | -2.02897 | 0.0207506 |  |
| WBGene00017335 | -2.02595 | 0.0249718 | sperm |
| WBGene00015560 | -2.02146 | 0.0483716 |  |
| WBGene00001159 | -2.02101 | 0.00243036 |  |
| WBGene00003446 | -2.02062 | 9.67E-05 | shared sperm |
| WBGene00002879 | -2.01735 | 0.00675394 |  |
| WBGene00016514 | -2.00972 | 0.00464186 |  |
| WBGene00020902 | -2.00507 | 0.000737652 |  |
| WBGene00016860 | -2.00414 | 0.0367662 |  |
| WBGene00018147 | -2.00303 | 0.0396559 |  |
| WBGene00012755 | -2.00171 | 0.036524 |  |
| WBGene00001328 | 2.01003 | 0.00277957 |  |
| WBGene00001651 | 2.01016 | 0.0180454 |  |
| WBGene00007513 | 2.01094 | 0.000564027 | herm oocyte |
| WBGene00010055 | 2.01242 | 0.00178332 |  |
| WBGene00002035 | 2.01735 | 0.0314495 | oocyte |
| WBGene00018840 | 2.0193 | 0.000472675 | male intrinsic |
| WBGene00004723 | 2.0219 | 0.0308663 | herm intrinsic |
| WBGene00017194 | 2.02198 | 0.00470534 |  |
| WBGene00022882 | 2.02406 | 0.00328797 | herm intrinsic |
| WBGene00021924 | 2.02594 | 0.0232318 |  |
| WBGene00003738 | 2.02611 | 0.0316472 |  |
| WBGene00043302 | 2.02636 | 0.00498985 |  |
| WBGene00010139 | 2.02818 | 0.0216589 |  |
| WBGene00017978 | 2.02963 | 0.00311668 | shared sperm |
| WBGene00006481 | 2.03882 | 0.0364539 | herm oocyte |
| WBGene00019520 | 2.04077 | 0.0491607 |  |
| WBGene00016659 | 2.04851 | 0.0221544 |  |
| WBGene00010013 | 2.0499 | 0.00676388 |  |
| WBGene00002263 | 2.05058 | 0.0359858 |  |
| WBGene00001584 | 2.05178 | 0.0116543 | herm intrinsic |
| WBGene00015818 | 2.05234 | 0.0116454 | herm intrinsic |
| WBGene00019458 | 2.05264 | 0.00924956 | herm oocyte |
| WBGene00005080 | 2.05582 | 0.0154083 |  |
| WBGene00014114 | 2.05719 | 0.028951 |  |
| WBGene00009366 | 2.06055 | 0.045326 | herm oocyte |
| WBGene00001263 | 2.06434 | 0.0463308 |  |
| WBGene00016429 | 2.07041 | 0.0149664 | sperm |
| WBGene00017236 | 2.07272 | 0.0426844 |  |
| WBGene00003804 | 2.07542 | 0.0154931 | herm oocyte |
| WBGene00010562 | 2.07554 | 0.039995 | herm oocyte |
| WBGene00012926 | 2.07654 | 0.0122866 |  |
| WBGene00020068 | 2.07741 | 0.045854 | herm intrinsic |
| WBGene00021559 | 2.07825 | 0.00835065 |  |
| WBGene00003789 | 2.07887 | 0.02242 | herm intrinsic |
| WBGene00007025 | 2.08273 | 0.00196638 |  |
| WBGene00010839 | 2.09039 | 0.00950294 | herm intrinsic |
| WBGene00044916 | 2.09213 | 0.00924187 |  |
| WBGene00011817 | 2.09703 | 0.0101069 |  |
| WBGene00003134 | 2.09988 | 0.0356272 | shared intrinsic |
| WBGene00019811 | 2.10039 | 0.000476402 | herm oocyte |
| WBGene00012373 | 2.10662 | 0.0333996 |  |
| WBGene00009779 | 2.10816 | 0.000317187 |  |
| WBGene00006779 | 2.10989 | 0.00500927 |  |
| WBGene00010467 | 2.11281 | 0.000917985 |  |
| WBGene00012352 | 2.11307 | 0.00418817 |  |
| WBGene00022749 | 2.11316 | 0.0366947 |  |
| WBGene00016960 | 2.11503 | 0.0192768 | herm intrinsic |
| WBGene00020256 | 2.12079 | 0.0337116 |  |
| WBGene00012555 | 2.12153 | 0.00637203 |  |
| WBGene00017567 | 2.12242 | 0.0496906 |  |
| WBGene00007000 | 2.12586 | 0.0011393 | oocyte |
| WBGene00001874 | 2.12739 | 0.00350033 | herm oocyte |
| WBGene00019345 | 2.13061 | 0.0115082 |  |
| WBGene00008119 | 2.13394 | 0.0341254 |  |
| WBGene00020556 | 2.13397 | 0.0135499 |  |
| WBGene00000998 | 2.1354 | 0.00552902 |  |
| WBGene00008863 | 2.13547 | 0.0471054 |  |
| WBGene00017803 | 2.13593 | 0.0285892 | sperm |
| WBGene00017042 | 2.13643 | 0.0252258 |  |
| WBGene00010583 | 2.1423 | 0.0335657 | herm oocyte |
| WBGene00008779 | 2.14266 | 0.0216729 |  |
| WBGene00004859 | 2.14825 | 0.00125295 |  |
| WBGene00011132 | 2.14859 | 0.00427611 |  |
| WBGene00017319 | 2.15581 | 0.0469287 |  |
| WBGene00011753 | 2.16022 | 0.0227085 |  |
| WBGene00020207 | 2.1629 | 0.0284561 |  |
| WBGene00001595 | 2.16705 | 0.0342445 | shared oocyte |
| WBGene00016816 | 2.16943 | 0.0434617 | herm oocyte |
| WBGene00001831 | 2.17136 | 0.0369158 | shared oocyte |
| WBGene00010776 | 2.18183 | 0.0200046 |  |
| WBGene00006324 | 2.18265 | 0.0287829 |  |
| WBGene00011915 | 2.18513 | 0.00499787 |  |
| WBGene00013978 | 2.18524 | 0.0454207 | shared sperm |
| WBGene00019572 | 2.18592 | 0.0204464 |  |
| WBGene00018064 | 2.18814 | 0.0145195 | herm intrinsic |
| WBGene00004391 | 2.19052 | 0.0160703 |  |
| WBGene00017547 | 2.19385 | 0.0407664 | herm oocyte |
| WBGene00002025 | 2.19864 | 0.0145751 |  |
| WBGene00000070 | 2.20006 | 0.0386529 |  |
| WBGene00017144 | 2.20165 | 0.00571969 |  |
| WBGene00003026 | 2.20223 | 0.0261378 | herm oocyte |
| WBGene00006571 | 2.20351 | 0.0080386 |  |
| WBGene00015476 | 2.20895 | 0.00333811 |  |
| WBGene00010334 | 2.21076 | 0.0175566 |  |
| WBGene00003421 | 2.21327 | 0.0481603 | herm oocyte |
| WBGene00012971 | 2.21436 | 0.0296349 | herm intrinsic |
| WBGene00018271 | 2.2161 | 0.00857748 |  |
| WBGene00017316 | 2.21804 | 0.0498063 | herm intrinsic |
| WBGene00007564 | 2.22153 | 0.00059372 |  |
| WBGene00008934 | 2.22544 | 0.0227412 |  |
| WBGene00019057 | 2.2282 | 0.00620936 |  |
| WBGene00004053 | 2.23143 | 0.0164621 | sperm |
| WBGene00000378 | 2.23482 | 0.011057 | herm intrinsic |
| WBGene00043056 | 2.23496 | 0.0439724 |  |
| WBGene00000511 | 2.23752 | 0.00132098 |  |
| WBGene00011831 | 2.23756 | 0.0351197 |  |
| WBGene00019792 | 2.23757 | 0.0113792 | herm intrinsic |
| WBGene00000250 | 2.24148 | 0.00412958 | shared oocyte |
| WBGene00010015 | 2.24238 | 0.012314 |  |
| WBGene00001974 | 2.24584 | 0.0257492 | herm oocyte |
| WBGene00009586 | 2.24733 | 0.00513154 |  |
| WBGene00008921 | 2.2493 | 0.00820855 | herm oocyte |
| WBGene00015547 | 2.25112 | 0.0172203 |  |
| WBGene00013717 | 2.25452 | 0.0257859 |  |
| WBGene00002497 | 2.25498 | 0.0070506 |  |
| WBGene00018337 | 2.25603 | 0.0316028 | herm oocyte |
| WBGene00011893 | 2.25783 | 0.0148357 |  |
| WBGene00006206 | 2.27532 | 0.00354485 |  |
| WBGene00019437 | 2.27654 | 0.0131873 |  |
| WBGene00016444 | 2.28238 | 0.00904296 | herm oocyte |
| WBGene00021886 | 2.28616 | 0.0143089 |  |
| WBGene00010621 | 2.28641 | 0.0448981 | herm oocyte |
| WBGene00016402 | 2.28659 | 0.021001 | herm intrinsic |
| WBGene00016142 | 2.28869 | 0.0131422 | herm intrinsic |
| WBGene00194818 | 2.29171 | 0.015523 |  |
| WBGene00007772 | 2.29366 | 0.000899781 |  |
| WBGene00013893 | 2.2939 | 0.000367628 |  |
| WBGene00021275 | 2.30033 | 0.0447842 |  |
| WBGene00001483 | 2.30496 | 0.0269641 | shared intrinsic |
| WBGene00002280 | 2.30961 | 0.00194692 |  |
| WBGene00002368 | 2.31164 | 0.01829 | herm oocyte |
| WBGene00020316 | 2.32197 | 0.00213354 | herm intrinsic |
| WBGene00015524 | 2.32385 | 0.029131 |  |
| WBGene00012608 | 2.32821 | 0.00428694 |  |
| WBGene00001740 | 2.33026 | 0.0440372 | herm intrinsic |
| WBGene00005143 | 2.33785 | 0.0203059 |  |
| WBGene00003230 | 2.33802 | 0.0457418 | shared oocyte |
| WBGene00020160 | 2.33957 | 0.00658025 |  |
| WBGene00016499 | 2.34223 | 0.00639643 |  |
| WBGene00007972 | 2.34461 | 0.00229788 | herm oocyte |
| WBGene00020085 | 2.35093 | 0.00848026 |  |
| WBGene00010988 | 2.35938 | 0.0182228 |  |
| WBGene00010176 | 2.3622 | 0.0141762 |  |
| WBGene00013720 | 2.36612 | 0.0264511 | oocyte |
| WBGene00009124 | 2.36986 | 0.0099655 | herm intrinsic |
| WBGene00001170 | 2.37475 | 0.0459871 |  |
| WBGene00010273 | 2.38038 | 0.0263837 |  |
| WBGene00002027 | 2.39121 | 0.0174682 |  |
| WBGene00009163 | 2.39533 | 0.037674 | herm oocyte |
| WBGene00017271 | 2.40111 | 0.0358004 |  |
| WBGene00020629 | 2.40125 | 0.000498725 | herm intrinsic |
| WBGene00009645 | 2.40144 | 0.0461848 |  |
| WBGene00021768 | 2.40753 | 0.0429845 |  |
| WBGene00017658 | 2.41579 | 0.0418626 |  |
| WBGene00015150 | 2.41897 | 0.0335072 |  |
| WBGene00014018 | 2.42108 | 0.00693733 |  |
| WBGene00019819 | 2.42256 | 0.0427845 | sperm |
| WBGene00020415 | 2.4266 | 0.0128132 |  |
| WBGene00019518 | 2.43091 | 0.0242589 |  |
| WBGene00007620 | 2.43675 | 0.00944299 | herm oocyte |
| WBGene00012460 | 2.44033 | 0.0189525 |  |
| WBGene00016211 | 2.44037 | 0.0190731 | herm intrinsic |
| WBGene00019595 | 2.45334 | 0.00868024 | herm oocyte |
| WBGene00007013 | 2.46094 | 0.00400193 |  |
| WBGene00018857 | 2.46374 | 0.00283183 |  |
| WBGene00003045 | 2.47004 | 0.0069388 | herm intrinsic |
| WBGene00003882 | 2.47459 | 0.00763082 | herm oocyte |
| WBGene00016700 | 2.47638 | 0.0242802 |  |
| WBGene00000753 | 2.47726 | 0.0211306 |  |
| WBGene00006935 | 2.47822 | 0.0426353 |  |
| WBGene00006349 | 2.49074 | 0.0467021 | oocyte |
| WBGene00006437 | 2.49199 | 0.00238193 | oocyte |
| WBGene00003499 | 2.49391 | 0.0456808 | herm intrinsic |
| WBGene00000959 | 2.4951 | 0.00222782 |  |
| WBGene00013810 | 2.49603 | 0.00421809 | shared sperm |
| WBGene00021201 | 2.52211 | 0.0378242 | herm intrinsic |
| WBGene00016966 | 2.53492 | 0.00832307 | herm oocyte |
| WBGene00019832 | 2.54312 | 0.0171348 |  |
| WBGene00000871 | 2.57339 | 0.0047985 | herm oocyte |
| WBGene00008143 | 2.57561 | 0.0299565 |  |
| WBGene00018497 | 2.57795 | 0.0333409 |  |
| WBGene00008081 | 2.59487 | 0.00264124 | herm oocyte |
| WBGene00008061 | 2.59955 | 0.0230245 | shared oocyte |
| WBGene00017627 | 2.60995 | 0.0349658 | sperm |
| WBGene00006489 | 2.62426 | 0.00516513 | herm oocyte |
| WBGene00004150 | 2.62433 | 0.00120595 |  |
| WBGene00012606 | 2.6315 | 0.00645904 |  |
| WBGene00004372 | 2.63974 | 0.041344 |  |
| WBGene00001996 | 2.64132 | 0.00270847 |  |
| WBGene00018425 | 2.64169 | 0.0202283 |  |
| WBGene00006474 | 2.64514 | 0.0426461 | herm oocyte |
| WBGene00020171 | 2.67414 | 0.00173831 |  |
| WBGene00006562 | 2.72073 | 0.0176492 | herm oocyte |
| WBGene00012538 | 2.72206 | 0.0110351 |  |
| WBGene00009668 | 2.74731 | 0.0333767 | herm oocyte |
| WBGene00010363 | 2.76558 | 0.0469498 | herm oocyte |
| WBGene00012524 | 2.79333 | 0.0173095 | herm intrinsic |
| WBGene00015549 | 2.80019 | 0.00328992 |  |
| WBGene00000196 | 2.82255 | 0.0199206 |  |
| WBGene00014256 | 2.83258 | 0.0167653 |  |
| WBGene00007709 | 2.84491 | 0.00994635 |  |
| WBGene00015002 | 2.84533 | 0.000616669 |  |
| WBGene00009604 | 2.8571 | 0.0310802 |  |
| WBGene00021624 | 2.86717 | 0.0166273 |  |
| WBGene00017771 | 2.87316 | 0.000712338 |  |
| WBGene00013418 | 2.87455 | 0.0113217 |  |
| WBGene00018393 | 2.88552 | 0.0279573 |  |
| WBGene00001481 | 2.89213 | 0.0167169 |  |
| WBGene00008497 | 2.89422 | 0.049752 | sperm |
| WBGene00002184 | 2.89706 | 0.00407927 |  |
| WBGene00013349 | 2.90365 | 0.00211733 |  |
| WBGene00013124 | 2.91723 | 0.0050074 |  |
| WBGene00016683 | 2.93445 | 0.000289062 |  |
| WBGene00022881 | 2.95799 | 0.000884649 | herm oocyte |
| WBGene00002047 | 2.95889 | 0.023094 |  |
| WBGene00009677 | 2.97948 | 0.0166546 |  |
| WBGene00023418 | 2.98207 | 0.0199612 |  |
| WBGene00020128 | 2.98507 | 0.0193957 |  |
| WBGene00010650 | 2.98866 | 0.000223433 | shared sperm |
| WBGene00015189 | 3.00411 | 0.0383917 |  |
| WBGene00000687 | 3.02748 | 0.000655602 |  |
| WBGene00007422 | 3.02987 | 0.017827 |  |
| WBGene00011923 | 3.03003 | 0.0158726 |  |
| WBGene00018050 | 3.03461 | 0.000973949 |  |
| WBGene00017152 | 3.03863 | 0.00192422 |  |
| WBGene00000377 | 3.05199 | 0.0348219 | herm oocyte |
| WBGene00004052 | 3.06337 | 0.000989863 |  |
| WBGene00018138 | 3.07526 | 0.0159985 |  |
| WBGene00018507 | 3.10818 | 0.00067363 |  |
| WBGene00020859 | 3.11184 | 0.00116271 |  |
| WBGene00022635 | 3.13478 | 0.00892825 |  |
| WBGene00008594 | 3.13667 | 0.0304263 |  |
| WBGene00020964 | 3.13963 | 0.041088 | herm intrinsic |
| WBGene00002055 | 3.15329 | 0.008365 |  |
| WBGene00011696 | 3.16334 | 0.00908387 | oocyte |
| WBGene00003798 | 3.16724 | 0.0190237 | herm oocyte |
| WBGene00016441 | 3.17046 | 0.00255727 | shared sperm |
| WBGene00004157 | 3.18011 | 0.00166182 |  |
| WBGene00001532 | 3.18034 | 0.000715393 |  |
| WBGene00003480 | 3.19283 | 0.00146645 |  |
| WBGene00015973 | 3.21662 | 0.00626577 |  |
| WBGene00000634 | 3.27445 | 0.00137508 |  |
| WBGene00016767 | 3.3034 | 0.00320174 |  |
| WBGene00007302 | 3.31851 | 0.0202431 |  |
| WBGene00009114 | 3.33138 | 0.000206421 |  |
| WBGene00011350 | 3.40071 | 0.000789473 | herm oocyte |
| WBGene00001310 | 3.40901 | 0.00942825 | oocyte |
| WBGene00011147 | 3.41288 | 0.000127474 |  |
| WBGene00009891 | 3.43737 | 0.00757105 |  |
| WBGene00022257 | 3.4389 | 0.0209834 |  |
| WBGene00018500 | 3.44662 | 0.00407691 | shared sperm |
| WBGene00006307 | 3.46608 | 0.000510487 | herm oocyte |
| WBGene00009395 | 3.48497 | 0.00588924 |  |
| WBGene00021967 | 3.52421 | 0.0409729 | male sperm |
| WBGene00011262 | 3.54282 | 0.030467 |  |
| WBGene00006445 | 3.55069 | 0.0020068 |  |
| WBGene00009780 | 3.55931 | 0.000188343 |  |
| WBGene00012785 | 3.5678 | 0.0102115 | shared sperm |
| WBGene00007347 | 3.67703 | 0.0107202 |  |
| WBGene00007304 | 3.69638 | 0.0100378 |  |
| WBGene00005681 | 3.70357 | 0.000391982 |  |
| WBGene00002130 | 3.72936 | 0.00156263 |  |
| WBGene00000465 | 3.7347 | 0.00539854 | shared oocyte |
| WBGene00000722 | 3.78015 | 0.0331162 |  |
| WBGene00015537 | 3.79588 | 0.0194699 |  |
| WBGene00006814 | 3.82862 | 0.0167017 |  |
| WBGene00006436 | 3.87968 | 0.000612879 | herm intrinsic |
| WBGene00017804 | 4.14298 | 0.00904185 |  |
| WBGene00008171 | 4.30023 | 0.00911011 |  |
| WBGene00017589 | 4.45222 | 0.00807561 |  |
| WBGene00018229 | 4.66912 | 0.0105649 |  |
| WBGene00195045 | 4.69788 | 0.010124 |  |
| WBGene00000397 | 4.83652 | 0.000718503 |  |
| WBGene00011227 | 4.95985 | 0.0203308 |  |
| WBGene00014074 | 5.14759 | 0.000160785 |  |
| WBGene00017871 | 5.45827 | 0.000154369 |  |
| WBGene00018295 | 5.72244 | 4.17E-05 |  |
| WBGene00019929 | 5.93425 | 0.00508933 | sperm |
| WBGene00004062 | 6.00341 | 0.0155444 |  |
| WBGene00014220 | 6.17479 | 0.000277753 | oocyte |
| WBGene00019353 | 6.74276 | 1.34E-05 |  |
| WBGene00011908 | 6.9802 | 0.00141519 | herm oocyte |
| WBGene00021027 | 7.01566 | 0.00141091 |  |
| WBGene00021780 | 7.55795 | 0.000132614 |  |
| WBGene00021620 | 8.9626 | 0.049761 |  |
| WBGene00001204 | 9.45776 | 1.58E-05 |  |
| WBGene00021619 | 13.9241 | 0.0106326 |  |
| WBGene00017876 | 15.1957 | 8.83E-10 |  |
