# Supplementary material for: SPE-44 Implements Sperm Cell Fate
Source: PLoS Genet. 2012 Apr 26;8(4):e1002678. doi: 10.1371/journal.pgen.1002678 (PMC3343087; doi:10.1371/journal.pgen.1002678)
Supplement: Table S3 — GO terms associated with spe-44 down-regulated genes. Listed are the genes from Table S2 with reduced expression in the spe-44 mutant strain, and all of the Gene Ontology terms associated with those genes. (DOC) [file pgen.1002678.s007.doc]

| **Table S3. GO terms associated with genes down-regulated in *spe-44*** | |
| --- | --- |
| **Wormbase Gene Identifier** | **GO term^a^** |
| WBGene00011467 | 2,3-dihydro-2,3-dihydroxybenzoate dehydrogenase activity |
| WBGene00005019 | 3-beta-hydroxy-delta5-steroid dehydrogenase activity |
| WBGene00008802 | acid phosphatase activity |
| WBGene00003185 | activation of JUN kinase activity |
| WBGene00003185 | activation of MAPK activity |
| WBGene00003185 | activation of MAPK activity |
| WBGene00018949 | acyl-CoA binding |
| WBGene00016491 | acyl-CoA dehydrogenase activity |
| WBGene00020419 | acyl-CoA dehydrogenase activity |
| WBGene00016511 | acyl-CoA metabolic process |
| WBGene00016511 | acyl-CoA thioesterase activity |
| WBGene00008581 | acyltransferase activity |
| WBGene00008629 | acyltransferase activity |
| WBGene00002879 | adenylate kinase activity |
| WBGene00001638 | alpha-1,3-mannosylglycoprotein 2-beta-N-acetylglucosaminyltransferase activity |
| WBGene00007825 | antioxidant activity |
| WBGene00019717 | antioxidant activity |
| WBGene00007756 | asparaginase activity |
| WBGene00001709 | ATP binding |
| WBGene00002208 | ATP binding |
| WBGene00002879 | ATP binding |
| WBGene00003185 | ATP binding |
| WBGene00006895 | ATP binding |
| WBGene00007269 | ATP binding |
| WBGene00007335 | ATP binding |
| WBGene00007777 | ATP binding |
| WBGene00008487 | ATP binding |
| WBGene00009039 | ATP binding |
| WBGene00009324 | ATP binding |
| WBGene00010056 | ATP binding |
| WBGene00010366 | ATP binding |
| WBGene00010883 | ATP binding |
| WBGene00011191 | ATP binding |
| WBGene00011466 | ATP binding |
| WBGene00011910 | ATP binding |
| WBGene00012010 | ATP binding |
| WBGene00012925 | ATP binding |
| WBGene00013701 | ATP binding |
| WBGene00014005 | ATP binding |
| WBGene00015049 | ATP binding |
| WBGene00015629 | ATP binding |
| WBGene00015934 | ATP binding |
| WBGene00016587 | ATP binding |
| WBGene00017050 | ATP binding |
| WBGene00017672 | ATP binding |
| WBGene00017802 | ATP binding |
| WBGene00018004 | ATP binding |
| WBGene00018122 | ATP binding |
| WBGene00018301 | ATP binding |
| WBGene00018999 | ATP binding |
| WBGene00019081 | ATP binding |
| WBGene00019461 | ATP binding |
| WBGene00020381 | ATP binding |
| WBGene00020435 | ATP binding |
| WBGene00020580 | ATP binding |
| WBGene00020992 | ATP binding |
| WBGene00021016 | ATP binding |
| WBGene00021151 | ATP binding |
| WBGene00022102 | ATP binding |
| WBGene00022108 | ATP binding |
| WBGene00022632 | ATP binding |
| WBGene00022707 | ATP binding |
| WBGene00022780 | ATP binding |
| WBGene00012925 | ATPase activity |
| WBGene00019461 | ATPase activity |
| WBGene00017195 | axon |
| WBGene00001178 | axon guidance |
| WBGene00013696 | axonal fasciculation |
| WBGene00020350 | axonal fasciculation |
| WBGene00001424 | binding |
| WBGene00005019 | binding |
| WBGene00007732 | binding |
| WBGene00008332 | binding |
| WBGene00008333 | binding |
| WBGene00008660 | binding |
| WBGene00009030 | binding |
| WBGene00009956 | binding |
| WBGene00011467 | binding |
| WBGene00011501 | binding |
| WBGene00012177 | binding |
| WBGene00012786 | binding |
| WBGene00013013 | binding |
| WBGene00013357 | binding |
| WBGene00015193 | binding |
| WBGene00016912 | binding |
| WBGene00017810 | binding |
| WBGene00018949 | binding |
| WBGene00019950 | binding |
| WBGene00005019 | biosynthetic process |
| WBGene00012295 | biosynthetic process |
| WBGene00021215 | biosynthetic process |
| WBGene00000584 | body morphogenesis |
| WBGene00001159 | body morphogenesis |
| WBGene00001249 | body morphogenesis |
| WBGene00004949 | body morphogenesis |
| WBGene00010623 | body morphogenesis |
| WBGene00009589 | calcium ion binding |
| WBGene00012233 | calcium-dependent cysteine-type endopeptidase activity |
| WBGene00001839 | carboxy-lyase activity |
| WBGene00001839 | carboxylic acid metabolic process |
| WBGene00001839 | catalytic activity |
| WBGene00005019 | catalytic activity |
| WBGene00007548 | catalytic activity |
| WBGene00008332 | catalytic activity |
| WBGene00008333 | catalytic activity |
| WBGene00011173 | catalytic activity |
| WBGene00011174 | catalytic activity |
| WBGene00011203 | catalytic activity |
| WBGene00011467 | catalytic activity |
| WBGene00012177 | catalytic activity |
| WBGene00012295 | catalytic activity |
| WBGene00012855 | catalytic activity |
| WBGene00021215 | catalytic activity |
| WBGene00022752 | catalytic activity |
| WBGene00008137 | cation transmembrane transporter activity |
| WBGene00009513 | cation transmembrane transporter activity |
| WBGene00008137 | cation transport |
| WBGene00009513 | cation transport |
| WBGene00013011 | cell communication |
| WBGene00000383 | cell division |
| WBGene00000865 | cell division |
| WBGene00001045 | cell redox homeostasis |
| WBGene00007825 | cell redox homeostasis |
| WBGene00009238 | cell redox homeostasis |
| WBGene00013547 | cell redox homeostasis |
| WBGene00019717 | cell redox homeostasis |
| WBGene00001839 | cellular amino acid and derivative metabolic process |
| WBGene00007756 | cellular amino acid metabolic process |
| WBGene00005019 | cellular metabolic process |
| WBGene00000383 | cellular response to DNA damage stimulus |
| WBGene00022108 | cellular response to DNA damage stimulus |
| WBGene00003185 | cellular response to stress |
| WBGene00006997 | centrosome |
| WBGene00006997 | centrosome localization |
| WBGene00000248 | chemosensory behavior |
| WBGene00001709 | chemotaxis |
| WBGene00010056 | chromosome |
| WBGene00006997 | chromosome segregation |
| WBGene00005019 | coenzyme binding |
| WBGene00010056 | condensed nuclear chromosome |
| WBGene00008629 | cysteine-type endopeptidase activity |
| WBGene00000383 | cytokinesis |
| WBGene00000865 | cytokinesis |
| WBGene00003185 | cytoplasm |
| WBGene00005019 | cytoplasm |
| WBGene00011910 | cytoplasm |
| WBGene00012297 | cytoplasm |
| WBGene00000383 | dephosphorylation |
| WBGene00009492 | dephosphorylation |
| WBGene00010636 | dephosphorylation |
| WBGene00015026 | dephosphorylation |
| WBGene00016942 | dephosphorylation |
| WBGene00022709 | dephosphorylation |
| WBGene00001249 | determination of adult life span |
| WBGene00007987 | determination of adult life span |
| WBGene00015093 | determination of adult life span |
| WBGene00015094 | determination of adult life span |
| WBGene00019530 | determination of adult life span |
| WBGene00021457 | determination of adult life span |
| WBGene00022090 | determination of adult life span |
| WBGene00000584 | determination of left/right symmetry |
| WBGene00000584 | DNA binding |
| WBGene00004949 | DNA binding |
| WBGene00007732 | DNA binding |
| WBGene00014240 | DNA binding |
| WBGene00015934 | DNA binding |
| WBGene00045484 | DNA replication initiation |
| WBGene00007916 | dopamine beta-monooxygenase activity |
| WBGene00005019 | dTDP-4-dehydrorhamnose reductase activity |
| WBGene00006997 | dynein light intermediate chain binding |
| WBGene00016860 | electron carrier activity |
| WBGene00001159 | embryonic body morphogenesis |
| WBGene00006895 | embryonic development |
| WBGene00010623 | embryonic development |
| WBGene00000248 | embryonic development ending in birth or egg hatching |
| WBGene00000383 | embryonic development ending in birth or egg hatching |
| WBGene00000865 | embryonic development ending in birth or egg hatching |
| WBGene00001159 | embryonic development ending in birth or egg hatching |
| WBGene00001249 | embryonic development ending in birth or egg hatching |
| WBGene00001424 | embryonic development ending in birth or egg hatching |
| WBGene00002879 | embryonic development ending in birth or egg hatching |
| WBGene00003055 | embryonic development ending in birth or egg hatching |
| WBGene00003429 | embryonic development ending in birth or egg hatching |
| WBGene00003430 | embryonic development ending in birth or egg hatching |
| WBGene00003431 | embryonic development ending in birth or egg hatching |
| WBGene00003442 | embryonic development ending in birth or egg hatching |
| WBGene00003443 | embryonic development ending in birth or egg hatching |
| WBGene00003457 | embryonic development ending in birth or egg hatching |
| WBGene00003470 | embryonic development ending in birth or egg hatching |
| WBGene00003936 | embryonic development ending in birth or egg hatching |
| WBGene00004949 | embryonic development ending in birth or egg hatching |
| WBGene00005019 | embryonic development ending in birth or egg hatching |
| WBGene00006895 | embryonic development ending in birth or egg hatching |
| WBGene00006997 | embryonic development ending in birth or egg hatching |
| WBGene00007777 | embryonic development ending in birth or egg hatching |
| WBGene00008433 | embryonic development ending in birth or egg hatching |
| WBGene00008529 | embryonic development ending in birth or egg hatching |
| WBGene00009030 | embryonic development ending in birth or egg hatching |
| WBGene00009039 | embryonic development ending in birth or egg hatching |
| WBGene00009442 | embryonic development ending in birth or egg hatching |
| WBGene00009469 | embryonic development ending in birth or egg hatching |
| WBGene00010056 | embryonic development ending in birth or egg hatching |
| WBGene00010596 | embryonic development ending in birth or egg hatching |
| WBGene00010623 | embryonic development ending in birth or egg hatching |
| WBGene00010719 | embryonic development ending in birth or egg hatching |
| WBGene00010829 | embryonic development ending in birth or egg hatching |
| WBGene00011001 | embryonic development ending in birth or egg hatching |
| WBGene00011191 | embryonic development ending in birth or egg hatching |
| WBGene00011501 | embryonic development ending in birth or egg hatching |
| WBGene00011533 | embryonic development ending in birth or egg hatching |
| WBGene00015678 | embryonic development ending in birth or egg hatching |
| WBGene00016336 | embryonic development ending in birth or egg hatching |
| WBGene00016942 | embryonic development ending in birth or egg hatching |
| WBGene00017195 | embryonic development ending in birth or egg hatching |
| WBGene00018999 | embryonic development ending in birth or egg hatching |
| WBGene00019084 | embryonic development ending in birth or egg hatching |
| WBGene00020416 | embryonic development ending in birth or egg hatching |
| WBGene00020550 | embryonic development ending in birth or egg hatching |
| WBGene00021871 | embryonic development ending in birth or egg hatching |
| WBGene00022617 | embryonic development ending in birth or egg hatching |
| WBGene00022699 | embryonic development ending in birth or egg hatching |
| WBGene00022760 | embryonic development ending in birth or egg hatching |
| WBGene00010915 | endoplasmic reticulum membrane |
| WBGene00011467 | enterobactin biosynthetic process |
| WBGene00001249 | epidermal cell fate specification |
| WBGene00004964 | establishment of nucleus localization |
| WBGene00005019 | extracellular polysaccharide biosynthetic process |
| WBGene00001159 | extracellular region |
| WBGene00002120 | extracellular region |
| WBGene00003055 | extracellular region |
| WBGene00015246 | extracellular region |
| WBGene00015516 | extracellular region |
| WBGene00017055 | extracellular region |
| WBGene00001185 | extracellular space |
| WBGene00016491 | FAD binding |
| WBGene00020419 | FAD binding |
| WBGene00003185 | female meiosis |
| WBGene00001185 | fibroblast growth factor receptor binding |
| WBGene00001185 | fibroblast growth factor receptor signaling pathway |
| WBGene00001709 | G-protein coupled receptor kinase activity |
| WBGene00010634 | G-protein coupled receptor protein signaling pathway |
| WBGene00016246 | G-protein coupled receptor protein signaling pathway |
| WBGene00008529 | gamete generation |
| WBGene00018999 | gamete generation |
| WBGene00006895 | gastrulation |
| WBGene00006895 | gastrulation with mouth forming first |
| WBGene00019151 | gluconeogenesis |
| WBGene00001638 | Golgi membrane |
| WBGene00000584 | gonad development |
| WBGene00000248 | growth |
| WBGene00000865 | growth |
| WBGene00002879 | growth |
| WBGene00003055 | growth |
| WBGene00003936 | growth |
| WBGene00004949 | growth |
| WBGene00006895 | growth |
| WBGene00007335 | growth |
| WBGene00008266 | growth |
| WBGene00009442 | growth |
| WBGene00010596 | growth |
| WBGene00010719 | growth |
| WBGene00011001 | growth |
| WBGene00011191 | growth |
| WBGene00014229 | growth |
| WBGene00017853 | growth |
| WBGene00001185 | growth factor activity |
| WBGene00000248 | GTP binding |
| WBGene00019151 | GTP binding |
| WBGene00000248 | GTPase activity |
| WBGene00001045 | heat shock protein binding |
| WBGene00016860 | heme binding |
| WBGene00000248 | hermaphrodite genitalia development |
| WBGene00000584 | hermaphrodite genitalia development |
| WBGene00001159 | hermaphrodite genitalia development |
| WBGene00002879 | hermaphrodite genitalia development |
| WBGene00004949 | hermaphrodite genitalia development |
| WBGene00006895 | hermaphrodite genitalia development |
| WBGene00010719 | hermaphrodite genitalia development |
| WBGene00014229 | hermaphrodite genitalia development |
| WBGene00016730 | hermaphrodite genitalia development |
| WBGene00018999 | hermaphrodite genitalia development |
| WBGene00019255 | hermaphrodite genitalia development |
| WBGene00007916 | histidine catabolic process |
| WBGene00002120 | hormone activity |
| WBGene00007354 | hydrolase activity |
| WBGene00007548 | hydrolase activity |
| WBGene00007763 | hydrolase activity |
| WBGene00008124 | hydrolase activity |
| WBGene00009054 | hydrolase activity |
| WBGene00009948 | hydrolase activity |
| WBGene00010265 | hydrolase activity |
| WBGene00012741 | hydrolase activity |
| WBGene00015661 | hydrolase activity |
| WBGene00017636 | hydrolase activity |
| WBGene00018359 | hydrolase activity |
| WBGene00018410 | hydrolase activity |
| WBGene00019255 | hydrolase activity |
| WBGene00020985 | hydrolase activity |
| WBGene00022617 | hydrolase activity |
| WBGene00001709 | hyperosmotic response |
| WBGene00001159 | identical protein binding |
| WBGene00000248 | inductive cell migration |
| WBGene00001159 | integral to membrane |
| WBGene00001424 | integral to membrane |
| WBGene00001638 | integral to membrane |
| WBGene00004899 | integral to membrane |
| WBGene00004904 | integral to membrane |
| WBGene00004908 | integral to membrane |
| WBGene00004964 | integral to membrane |
| WBGene00005019 | integral to membrane |
| WBGene00006487 | integral to membrane |
| WBGene00006645 | integral to membrane |
| WBGene00006661 | integral to membrane |
| WBGene00006997 | integral to membrane |
| WBGene00007159 | integral to membrane |
| WBGene00007224 | integral to membrane |
| WBGene00007382 | integral to membrane |
| WBGene00007383 | integral to membrane |
| WBGene00007478 | integral to membrane |
| WBGene00007549 | integral to membrane |
| WBGene00007567 | integral to membrane |
| WBGene00007571 | integral to membrane |
| WBGene00007666 | integral to membrane |
| WBGene00007888 | integral to membrane |
| WBGene00007987 | integral to membrane |
| WBGene00008137 | integral to membrane |
| WBGene00008433 | integral to membrane |
| WBGene00008581 | integral to membrane |
| WBGene00008606 | integral to membrane |
| WBGene00009016 | integral to membrane |
| WBGene00009030 | integral to membrane |
| WBGene00009042 | integral to membrane |
| WBGene00009125 | integral to membrane |
| WBGene00009149 | integral to membrane |
| WBGene00009491 | integral to membrane |
| WBGene00009513 | integral to membrane |
| WBGene00009592 | integral to membrane |
| WBGene00009646 | integral to membrane |
| WBGene00009708 | integral to membrane |
| WBGene00009782 | integral to membrane |
| WBGene00010014 | integral to membrane |
| WBGene00010599 | integral to membrane |
| WBGene00010612 | integral to membrane |
| WBGene00010634 | integral to membrane |
| WBGene00010829 | integral to membrane |
| WBGene00010900 | integral to membrane |
| WBGene00010915 | integral to membrane |
| WBGene00011001 | integral to membrane |
| WBGene00011088 | integral to membrane |
| WBGene00011533 | integral to membrane |
| WBGene00011619 | integral to membrane |
| WBGene00011968 | integral to membrane |
| WBGene00012122 | integral to membrane |
| WBGene00012339 | integral to membrane |
| WBGene00012627 | integral to membrane |
| WBGene00012784 | integral to membrane |
| WBGene00012811 | integral to membrane |
| WBGene00012925 | integral to membrane |
| WBGene00013011 | integral to membrane |
| WBGene00013089 | integral to membrane |
| WBGene00013192 | integral to membrane |
| WBGene00013198 | integral to membrane |
| WBGene00013318 | integral to membrane |
| WBGene00013458 | integral to membrane |
| WBGene00013473 | integral to membrane |
| WBGene00013713 | integral to membrane |
| WBGene00013888 | integral to membrane |
| WBGene00014129 | integral to membrane |
| WBGene00015032 | integral to membrane |
| WBGene00015033 | integral to membrane |
| WBGene00015084 | integral to membrane |
| WBGene00015093 | integral to membrane |
| WBGene00015215 | integral to membrane |
| WBGene00015386 | integral to membrane |
| WBGene00015560 | integral to membrane |
| WBGene00015627 | integral to membrane |
| WBGene00015997 | integral to membrane |
| WBGene00016054 | integral to membrane |
| WBGene00016083 | integral to membrane |
| WBGene00016246 | integral to membrane |
| WBGene00016336 | integral to membrane |
| WBGene00016351 | integral to membrane |
| WBGene00016357 | integral to membrane |
| WBGene00016514 | integral to membrane |
| WBGene00016613 | integral to membrane |
| WBGene00016753 | integral to membrane |
| WBGene00016860 | integral to membrane |
| WBGene00016950 | integral to membrane |
| WBGene00017114 | integral to membrane |
| WBGene00017139 | integral to membrane |
| WBGene00017156 | integral to membrane |
| WBGene00017553 | integral to membrane |
| WBGene00017654 | integral to membrane |
| WBGene00017703 | integral to membrane |
| WBGene00017815 | integral to membrane |
| WBGene00017853 | integral to membrane |
| WBGene00018121 | integral to membrane |
| WBGene00018147 | integral to membrane |
| WBGene00018165 | integral to membrane |
| WBGene00018168 | integral to membrane |
| WBGene00018253 | integral to membrane |
| WBGene00018332 | integral to membrane |
| WBGene00018498 | integral to membrane |
| WBGene00018773 | integral to membrane |
| WBGene00019024 | integral to membrane |
| WBGene00019062 | integral to membrane |
| WBGene00019080 | integral to membrane |
| WBGene00019135 | integral to membrane |
| WBGene00019405 | integral to membrane |
| WBGene00019425 | integral to membrane |
| WBGene00019623 | integral to membrane |
| WBGene00020066 | integral to membrane |
| WBGene00020353 | integral to membrane |
| WBGene00020370 | integral to membrane |
| WBGene00020416 | integral to membrane |
| WBGene00020433 | integral to membrane |
| WBGene00020468 | integral to membrane |
| WBGene00020656 | integral to membrane |
| WBGene00020737 | integral to membrane |
| WBGene00020904 | integral to membrane |
| WBGene00020913 | integral to membrane |
| WBGene00021294 | integral to membrane |
| WBGene00021298 | integral to membrane |
| WBGene00021537 | integral to membrane |
| WBGene00021630 | integral to membrane |
| WBGene00022008 | integral to membrane |
| WBGene00022090 | integral to membrane |
| WBGene00022143 | integral to membrane |
| WBGene00022243 | integral to membrane |
| WBGene00022385 | integral to membrane |
| WBGene00022440 | integral to membrane |
| WBGene00022467 | integral to membrane |
| WBGene00022833 | integral to membrane |
| WBGene00023424 | integral to membrane |
| WBGene00043187 | integral to membrane |
| WBGene00044062 | integral to membrane |
| WBGene00044475 | integral to membrane |
| WBGene00050879 | integral to membrane |
| WBGene00050896 | integral to membrane |
| WBGene00001159 | integral to plasma membrane |
| WBGene00004904 | integral to plasma membrane |
| WBGene00004908 | integral to plasma membrane |
| WBGene00012787 | integral to plasma membrane |
| WBGene00002052 | intermediate filament |
| WBGene00009149 | intracellular |
| WBGene00011533 | intracellular |
| WBGene00011619 | intracellular |
| WBGene00012233 | intracellular |
| WBGene00014240 | intracellular |
| WBGene00015084 | intracellular |
| WBGene00044989 | intracellular |
| WBGene00001178 | iron ion binding |
| WBGene00016860 | iron ion binding |
| WBGene00003185 | JUN kinase kinase activity |
| WBGene00001178 | L-ascorbic acid binding |
| WBGene00001159 | lipid catabolic process |
| WBGene00016288 | lipid catabolic process |
| WBGene00010915 | lipid metabolic process |
| WBGene00003429 | lipid storage |
| WBGene00003430 | lipid storage |
| WBGene00003431 | lipid storage |
| WBGene00003438 | lipid storage |
| WBGene00003470 | lipid storage |
| WBGene00006719 | lipid storage |
| WBGene00007354 | lipid storage |
| WBGene00008433 | lipid storage |
| WBGene00011191 | lipid storage |
| WBGene00011466 | lipid storage |
| WBGene00012177 | lipid storage |
| WBGene00015032 | lipid storage |
| WBGene00015049 | lipid storage |
| WBGene00017195 | lipid storage |
| WBGene00017703 | lipid storage |
| WBGene00019151 | lipid storage |
| WBGene00020416 | lipid storage |
| WBGene00020550 | lipid storage |
| WBGene00021298 | lipid storage |
| WBGene00022760 | lipid storage |
| WBGene00044062 | lipid storage |
| WBGene00016587 | lipopolysaccharide biosynthetic process |
| WBGene00004964 | localization |
| WBGene00000865 | locomotion |
| WBGene00001159 | locomotion |
| WBGene00001249 | locomotion |
| WBGene00004949 | locomotion |
| WBGene00006051 | locomotion |
| WBGene00006895 | locomotion |
| WBGene00007198 | locomotion |
| WBGene00007354 | locomotion |
| WBGene00008433 | locomotion |
| WBGene00009549 | locomotion |
| WBGene00010719 | locomotion |
| WBGene00011191 | locomotion |
| WBGene00012877 | locomotion |
| WBGene00014229 | locomotion |
| WBGene00016698 | locomotion |
| WBGene00016730 | locomotion |
| WBGene00044989 | locomotion |
| WBGene00010915 | lysophospholipase activity |
| WBGene00003185 | male meiosis |
| WBGene00003185 | MAP kinase kinase activity |
| WBGene00003185 | MAP kinase kinase activity |
| WBGene00003185 | MAPKKK cascade |
| WBGene00003185 | MAPKKK cascade |
| WBGene00004904 | membrane |
| WBGene00004908 | membrane |
| WBGene00006487 | membrane |
| WBGene00006661 | membrane |
| WBGene00008137 | membrane |
| WBGene00009513 | membrane |
| WBGene00012786 | membrane |
| WBGene00012787 | membrane |
| WBGene00012925 | membrane |
| WBGene00016587 | membrane |
| WBGene00018168 | membrane |
| WBGene00021294 | membrane |
| WBGene00004964 | membrane fusion |
| WBGene00004964 | membrane-bounded organelle |
| WBGene00005019 | metabolic process |
| WBGene00007440 | metabolic process |
| WBGene00007548 | metabolic process |
| WBGene00008332 | metabolic process |
| WBGene00008333 | metabolic process |
| WBGene00008581 | metabolic process |
| WBGene00011088 | metabolic process |
| WBGene00011173 | metabolic process |
| WBGene00011174 | metabolic process |
| WBGene00011467 | metabolic process |
| WBGene00012177 | metabolic process |
| WBGene00012855 | metabolic process |
| WBGene00016491 | metabolic process |
| WBGene00019255 | metabolic process |
| WBGene00020419 | metabolic process |
| WBGene00022752 | metabolic process |
| WBGene00006487 | metal ion transmembrane transporter activity |
| WBGene00006487 | metal ion transport |
| WBGene00017553 | metalloendopeptidase activity |
| WBGene00017553 | metallopeptidase activity |
| WBGene00000248 | microtubule |
| WBGene00000248 | microtubule |
| WBGene00013712 | microtubule associated complex |
| WBGene00013712 | microtubule motor activity |
| WBGene00000248 | microtubule-based movement |
| WBGene00000248 | microtubule-based process |
| WBGene00000248 | microtubule-based process |
| WBGene00013712 | microtubule-based process |
| WBGene00000383 | midbody |
| WBGene00003936 | molting cycle, collagen and cuticulin-based cuticle |
| WBGene00010596 | molting cycle, collagen and cuticulin-based cuticle |
| WBGene00010719 | molting cycle, collagen and cuticulin-based cuticle |
| WBGene00011088 | monooxygenase activity |
| WBGene00016860 | monooxygenase activity |
| WBGene00000248 | morphogenesis of an epithelium |
| WBGene00001159 | morphogenesis of an epithelium |
| WBGene00001249 | morphogenesis of an epithelium |
| WBGene00004949 | morphogenesis of an epithelium |
| WBGene00005019 | morphogenesis of an epithelium |
| WBGene00006895 | morphogenesis of an epithelium |
| WBGene00014229 | morphogenesis of an epithelium |
| WBGene00001159 | multicellular organismal development |
| WBGene00001709 | multicellular organismal process |
| WBGene00012295 | NAD biosynthetic process |
| WBGene00003055 | negative regulation of DNA endoreduplication |
| WBGene00000865 | negative regulation of multicellular organism growth |
| WBGene00003055 | negative regulation of multicellular organism growth |
| WBGene00003055 | negative regulation of multicellular organism growth |
| WBGene00010719 | negative regulation of multicellular organism growth |
| WBGene00001159 | negative regulation of vulval development |
| WBGene00000248 | nematode larval development |
| WBGene00000865 | nematode larval development |
| WBGene00002879 | nematode larval development |
| WBGene00003055 | nematode larval development |
| WBGene00003936 | nematode larval development |
| WBGene00004949 | nematode larval development |
| WBGene00005019 | nematode larval development |
| WBGene00006895 | nematode larval development |
| WBGene00007335 | nematode larval development |
| WBGene00008266 | nematode larval development |
| WBGene00008433 | nematode larval development |
| WBGene00009442 | nematode larval development |
| WBGene00010596 | nematode larval development |
| WBGene00010719 | nematode larval development |
| WBGene00011001 | nematode larval development |
| WBGene00011191 | nematode larval development |
| WBGene00014229 | nematode larval development |
| WBGene00017195 | nematode larval development |
| WBGene00017853 | nematode larval development |
| WBGene00004904 | neurotransmitter transport |
| WBGene00004908 | neurotransmitter transport |
| WBGene00012787 | neurotransmitter transport |
| WBGene00004904 | neurotransmitter:sodium symporter activity |
| WBGene00004908 | neurotransmitter:sodium symporter activity |
| WBGene00012787 | neurotransmitter:sodium symporter activity |
| WBGene00006997 | nuclear envelope |
| WBGene00000383 | nucleic acid binding |
| WBGene00002879 | nucleobase, nucleoside, nucleotide and nucleic acid metabolic process |
| WBGene00002879 | nucleobase, nucleoside, nucleotide kinase activity |
| WBGene00010056 | nucleoplasm |
| WBGene00012925 | nucleoside-triphosphatase activity |
| WBGene00019461 | nucleoside-triphosphatase activity |
| WBGene00014240 | nucleosome |
| WBGene00014240 | nucleosome assembly |
| WBGene00012925 | nucleotide binding |
| WBGene00019461 | nucleotide binding |
| WBGene00002879 | nucleotide kinase activity |
| WBGene00012295 | nucleotidyltransferase activity |
| WBGene00021215 | nucleotidyltransferase activity |
| WBGene00000222 | nucleus |
| WBGene00000584 | nucleus |
| WBGene00000865 | nucleus |
| WBGene00001249 | nucleus |
| WBGene00004949 | nucleus |
| WBGene00007732 | nucleus |
| WBGene00014240 | nucleus |
| WBGene00006997 | nucleus localization |
| WBGene00001709 | olfactory behavior |
| WBGene00003185 | oocyte differentiation |
| WBGene00000865 | oocyte maturation |
| WBGene00000865 | oogenesis |
| WBGene00004964 | organelle organization |
| WBGene00000584 | oviposition |
| WBGene00001159 | oviposition |
| WBGene00001249 | oviposition |
| WBGene00004949 | oviposition |
| WBGene00005019 | oviposition |
| WBGene00006997 | oviposition |
| WBGene00001178 | oxidation reduction |
| WBGene00004739 | oxidation reduction |
| WBGene00007440 | oxidation reduction |
| WBGene00008332 | oxidation reduction |
| WBGene00008333 | oxidation reduction |
| WBGene00011088 | oxidation reduction |
| WBGene00011467 | oxidation reduction |
| WBGene00012177 | oxidation reduction |
| WBGene00016491 | oxidation reduction |
| WBGene00016860 | oxidation reduction |
| WBGene00020419 | oxidation reduction |
| WBGene00001178 | oxidoreductase activity |
| WBGene00004739 | oxidoreductase activity |
| WBGene00007440 | oxidoreductase activity |
| WBGene00007825 | oxidoreductase activity |
| WBGene00008332 | oxidoreductase activity |
| WBGene00008333 | oxidoreductase activity |
| WBGene00011088 | oxidoreductase activity |
| WBGene00011467 | oxidoreductase activity |
| WBGene00012177 | oxidoreductase activity |
| WBGene00016491 | oxidoreductase activity |
| WBGene00019717 | oxidoreductase activity |
| WBGene00020419 | oxidoreductase activity |
| WBGene00001178 | oxidoreductase activity, acting on paired donors, with incorporation or reduction of molecular oxygen |
| WBGene00016491 | oxidoreductase activity, acting on the CH-CH group of donors |
| WBGene00020419 | oxidoreductase activity, acting on the CH-CH group of donors |
| WBGene00011910 | P granule |
| WBGene00000584 | pattern specification process |
| WBGene00001159 | pharyngeal muscle development |
| WBGene00005019 | pharynx development |
| WBGene00000383 | phosphatase activity |
| WBGene00009492 | phosphatase activity |
| WBGene00010636 | phosphatase activity |
| WBGene00015026 | phosphatase activity |
| WBGene00016942 | phosphatase activity |
| WBGene00022709 | phosphatase activity |
| WBGene00010915 | phosphatidylcholine metabolic process |
| WBGene00019151 | phosphoenolpyruvate carboxykinase activity |
| WBGene00013011 | phosphoinositide binding |
| WBGene00001159 | phospholipase A2 activity |
| WBGene00016288 | phospholipase A2 activity |
| WBGene00001159 | phospholipid metabolic process |
| WBGene00016288 | phospholipid metabolic process |
| WBGene00012012 | phosphopantetheine binding |
| WBGene00016587 | phosphotransferase activity, alcohol group as acceptor |
| WBGene00002879 | phosphotransferase activity, phosphate group as acceptor |
| WBGene00001159 | plasma membrane fusion |
| WBGene00004964 | positive regulation of cell size |
| WBGene00000383 | positive regulation of growth rate |
| WBGene00001709 | positive regulation of growth rate |
| WBGene00002879 | positive regulation of growth rate |
| WBGene00003055 | positive regulation of growth rate |
| WBGene00003055 | positive regulation of growth rate |
| WBGene00003431 | positive regulation of growth rate |
| WBGene00003435 | positive regulation of growth rate |
| WBGene00003470 | positive regulation of growth rate |
| WBGene00003936 | positive regulation of growth rate |
| WBGene00004739 | positive regulation of growth rate |
| WBGene00004949 | positive regulation of growth rate |
| WBGene00006895 | positive regulation of growth rate |
| WBGene00007335 | positive regulation of growth rate |
| WBGene00007574 | positive regulation of growth rate |
| WBGene00008433 | positive regulation of growth rate |
| WBGene00008529 | positive regulation of growth rate |
| WBGene00009039 | positive regulation of growth rate |
| WBGene00009469 | positive regulation of growth rate |
| WBGene00009589 | positive regulation of growth rate |
| WBGene00010623 | positive regulation of growth rate |
| WBGene00010779 | positive regulation of growth rate |
| WBGene00011001 | positive regulation of growth rate |
| WBGene00011405 | positive regulation of growth rate |
| WBGene00014229 | positive regulation of growth rate |
| WBGene00015193 | positive regulation of growth rate |
| WBGene00017501 | positive regulation of growth rate |
| WBGene00017853 | positive regulation of growth rate |
| WBGene00018999 | positive regulation of growth rate |
| WBGene00019834 | positive regulation of growth rate |
| WBGene00022760 | positive regulation of growth rate |
| WBGene00007987 | positive regulation of locomotion |
| WBGene00008433 | positive regulation of locomotion |
| WBGene00000248 | positive regulation of multicellular organism growth |
| WBGene00001159 | positive regulation of multicellular organism growth |
| WBGene00002879 | positive regulation of multicellular organism growth |
| WBGene00006039 | positive regulation of multicellular organism growth |
| WBGene00006997 | positive regulation of multicellular organism growth |
| WBGene00010719 | positive regulation of multicellular organism growth |
| WBGene00001249 | positive regulation of specific transcription from RNA polymerase II promoter |
| WBGene00001249 | positive regulation of transcription from RNA polymerase II promoter |
| WBGene00000584 | positive regulation of vulval development |
| WBGene00001159 | post-embryonic body morphogenesis |
| WBGene00006704 | post-translational protein modification |
| WBGene00006718 | post-translational protein modification |
| WBGene00006719 | post-translational protein modification |
| WBGene00006661 | potassium channel activity |
| WBGene00006661 | potassium ion transport |
| WBGene00000248 | pronuclear migration |
| WBGene00002879 | pronuclear migration |
| WBGene00006997 | pronuclear migration |
| WBGene00000383 | **protein amino acid dephosphorylation** |
| WBGene00007610 | **protein amino acid dephosphorylation** |
| WBGene00009492 | **protein amino acid dephosphorylation** |
| WBGene00009548 | **protein amino acid dephosphorylation** |
| WBGene00010634 | **protein amino acid dephosphorylation** |
| WBGene00010636 | **protein amino acid dephosphorylation** |
| WBGene00015026 | **protein amino acid dephosphorylation** |
| WBGene00016698 | **protein amino acid dephosphorylation** |
| WBGene00016742 | **protein amino acid dephosphorylation** |
| WBGene00016942 | **protein amino acid dephosphorylation** |
| WBGene00018347 | **protein amino acid dephosphorylation** |
| WBGene00022090 | **protein amino acid dephosphorylation** |
| WBGene00022709 | **protein amino acid dephosphorylation** |
| WBGene00001638 | protein amino acid N-linked glycosylation |
| WBGene00001709 | **protein amino acid phosphorylation** |
| WBGene00002208 | **protein amino acid phosphorylation** |
| WBGene00003185 | **protein amino acid phosphorylation** |
| WBGene00006895 | **protein amino acid phosphorylation** |
| WBGene00007269 | **protein amino acid phosphorylation** |
| WBGene00007335 | **protein amino acid phosphorylation** |
| WBGene00007777 | **protein amino acid phosphorylation** |
| WBGene00008487 | **protein amino acid phosphorylation** |
| WBGene00009039 | **protein amino acid phosphorylation** |
| WBGene00009324 | **protein amino acid phosphorylation** |
| WBGene00010366 | **protein amino acid phosphorylation** |
| WBGene00010883 | **protein amino acid phosphorylation** |
| WBGene00011191 | **protein amino acid phosphorylation** |
| WBGene00011466 | **protein amino acid phosphorylation** |
| WBGene00012010 | **protein amino acid phosphorylation** |
| WBGene00013701 | **protein amino acid phosphorylation** |
| WBGene00014005 | **protein amino acid phosphorylation** |
| WBGene00015049 | **protein amino acid phosphorylation** |
| WBGene00015629 | **protein amino acid phosphorylation** |
| WBGene00017050 | **protein amino acid phosphorylation** |
| WBGene00017672 | **protein amino acid phosphorylation** |
| WBGene00017802 | **protein amino acid phosphorylation** |
| WBGene00018004 | **protein amino acid phosphorylation** |
| WBGene00018122 | **protein amino acid phosphorylation** |
| WBGene00018301 | **protein amino acid phosphorylation** |
| WBGene00018999 | **protein amino acid phosphorylation** |
| WBGene00019081 | **protein amino acid phosphorylation** |
| WBGene00020381 | **protein amino acid phosphorylation** |
| WBGene00020435 | **protein amino acid phosphorylation** |
| WBGene00020580 | **protein amino acid phosphorylation** |
| WBGene00020992 | **protein amino acid phosphorylation** |
| WBGene00021016 | **protein amino acid phosphorylation** |
| WBGene00021151 | **protein amino acid phosphorylation** |
| WBGene00022102 | **protein amino acid phosphorylation** |
| WBGene00022108 | **protein amino acid phosphorylation** |
| WBGene00022632 | **protein amino acid phosphorylation** |
| WBGene00022707 | **protein amino acid phosphorylation** |
| WBGene00022780 | **protein amino acid phosphorylation** |
| WBGene00002208 | protein binding |
| WBGene00005013 | protein binding |
| WBGene00006895 | protein binding |
| WBGene00007574 | protein binding |
| WBGene00007633 | protein binding |
| WBGene00007666 | protein binding |
| WBGene00008266 | protein binding |
| WBGene00008487 | protein binding |
| WBGene00008629 | protein binding |
| WBGene00009039 | protein binding |
| WBGene00012010 | protein binding |
| WBGene00012297 | protein binding |
| WBGene00012873 | protein binding |
| WBGene00013011 | protein binding |
| WBGene00013461 | protein binding |
| WBGene00014005 | protein binding |
| WBGene00015516 | protein binding |
| WBGene00015937 | protein binding |
| WBGene00016320 | protein binding |
| WBGene00018999 | protein binding |
| WBGene00019081 | protein binding |
| WBGene00020992 | protein binding |
| WBGene00021151 | protein binding |
| WBGene00022780 | protein binding |
| WBGene00007574 | protein catabolic process |
| WBGene00000248 | protein complex |
| WBGene00000222 | protein dimerization activity |
| WBGene00001045 | protein folding |
| WBGene00006047 | protein homodimerization activity |
| WBGene00001709 | **protein kinase activity** |
| WBGene00002208 | **protein kinase activity** |
| WBGene00003185 | **protein kinase activity** |
| WBGene00006895 | **protein kinase activity** |
| WBGene00007269 | **protein kinase activity** |
| WBGene00007335 | **protein kinase activity** |
| WBGene00007777 | **protein kinase activity** |
| WBGene00008487 | **protein kinase activity** |
| WBGene00009039 | **protein kinase activity** |
| WBGene00009324 | **protein kinase activity** |
| WBGene00010366 | **protein kinase activity** |
| WBGene00010883 | **protein kinase activity** |
| WBGene00011191 | **protein kinase activity** |
| WBGene00011466 | **protein kinase activity** |
| WBGene00012010 | **protein kinase activity** |
| WBGene00013701 | **protein kinase activity** |
| WBGene00014005 | **protein kinase activity** |
| WBGene00015049 | **protein kinase activity** |
| WBGene00015629 | **protein kinase activity** |
| WBGene00017050 | **protein kinase activity** |
| WBGene00017672 | **protein kinase activity** |
| WBGene00017802 | **protein kinase activity** |
| WBGene00018004 | **protein kinase activity** |
| WBGene00018122 | **protein kinase activity** |
| WBGene00018301 | **protein kinase activity** |
| WBGene00018999 | **protein kinase activity** |
| WBGene00019081 | **protein kinase activity** |
| WBGene00020381 | **protein kinase activity** |
| WBGene00020435 | **protein kinase activity** |
| WBGene00020580 | **protein kinase activity** |
| WBGene00020992 | **protein kinase activity** |
| WBGene00021016 | **protein kinase activity** |
| WBGene00021151 | **protein kinase activity** |
| WBGene00022102 | **protein kinase activity** |
| WBGene00022108 | **protein kinase activity** |
| WBGene00022632 | **protein kinase activity** |
| WBGene00022707 | **protein kinase activity** |
| WBGene00022780 | **protein kinase activity** |
| WBGene00000248 | protein polymerization |
| WBGene00006997 | protein self-association |
| WBGene00001709 | **protein serine/threonine kinase activity** |
| WBGene00002208 | **protein serine/threonine kinase activity** |
| WBGene00003185 | **protein serine/threonine kinase activity** |
| WBGene00006895 | **protein serine/threonine kinase activity** |
| WBGene00007269 | **protein serine/threonine kinase activity** |
| WBGene00007335 | **protein serine/threonine kinase activity** |
| WBGene00007777 | **protein serine/threonine kinase activity** |
| WBGene00008487 | **protein serine/threonine kinase activity** |
| WBGene00009039 | **protein serine/threonine kinase activity** |
| WBGene00009324 | **protein serine/threonine kinase activity** |
| WBGene00010366 | **protein serine/threonine kinase activity** |
| WBGene00010883 | **protein serine/threonine kinase activity** |
| WBGene00011191 | **protein serine/threonine kinase activity** |
| WBGene00011466 | **protein serine/threonine kinase activity** |
| WBGene00012010 | **protein serine/threonine kinase activity** |
| WBGene00013701 | **protein serine/threonine kinase activity** |
| WBGene00014005 | **protein serine/threonine kinase activity** |
| WBGene00015049 | **protein serine/threonine kinase activity** |
| WBGene00015629 | **protein serine/threonine kinase activity** |
| WBGene00017050 | **protein serine/threonine kinase activity** |
| WBGene00017672 | **protein serine/threonine kinase activity** |
| WBGene00017802 | **protein serine/threonine kinase activity** |
| WBGene00018004 | **protein serine/threonine kinase activity** |
| WBGene00018122 | **protein serine/threonine kinase activity** |
| WBGene00018301 | **protein serine/threonine kinase activity** |
| WBGene00018999 | **protein serine/threonine kinase activity** |
| WBGene00019081 | **protein serine/threonine kinase activity** |
| WBGene00020381 | **protein serine/threonine kinase activity** |
| WBGene00020435 | **protein serine/threonine kinase activity** |
| WBGene00020580 | **protein serine/threonine kinase activity** |
| WBGene00020992 | **protein serine/threonine kinase activity** |
| WBGene00021016 | **protein serine/threonine kinase activity** |
| WBGene00021151 | **protein serine/threonine kinase activity** |
| WBGene00022102 | **protein serine/threonine kinase activity** |
| WBGene00022108 | **protein serine/threonine kinase activity** |
| WBGene00022632 | **protein serine/threonine kinase activity** |
| WBGene00022707 | **protein serine/threonine kinase activity** |
| WBGene00022780 | **protein serine/threonine kinase activity** |
| WBGene00002208 | **protein tyrosine kinase activity** |
| WBGene00006895 | **protein tyrosine kinase activity** |
| WBGene00008487 | **protein tyrosine kinase activity** |
| WBGene00009039 | **protein tyrosine kinase activity** |
| WBGene00012010 | **protein tyrosine kinase activity** |
| WBGene00014005 | **protein tyrosine kinase activity** |
| WBGene00018999 | **protein tyrosine kinase activity** |
| WBGene00019081 | **protein tyrosine kinase activity** |
| WBGene00021151 | **protein tyrosine kinase activity** |
| WBGene00022780 | **protein tyrosine kinase activity** |
| WBGene00000383 | **protein tyrosine phosphatase activity** |
| WBGene00007610 | **protein tyrosine phosphatase activity** |
| WBGene00009492 | **protein tyrosine phosphatase activity** |
| WBGene00009548 | **protein tyrosine phosphatase activity** |
| WBGene00010634 | **protein tyrosine phosphatase activity** |
| WBGene00010636 | **protein tyrosine phosphatase activity** |
| WBGene00015026 | **protein tyrosine phosphatase activity** |
| WBGene00016698 | **protein tyrosine phosphatase activity** |
| WBGene00016742 | **protein tyrosine phosphatase activity** |
| WBGene00016942 | **protein tyrosine phosphatase activity** |
| WBGene00018347 | **protein tyrosine phosphatase activity** |
| WBGene00022090 | **protein tyrosine phosphatase activity** |
| WBGene00022709 | **protein tyrosine phosphatase activity** |
| WBGene00000383 | **protein tyrosine/serine/threonine phosphatase activity** |
| WBGene00010636 | **protein tyrosine/serine/threonine phosphatase activity** |
| WBGene00016942 | **protein tyrosine/serine/threonine phosphatase activity** |
| WBGene00022090 | **protein tyrosine/serine/threonine phosphatase activity** |
| WBGene00006624 | proteolysis |
| WBGene00006625 | proteolysis |
| WBGene00008629 | proteolysis |
| WBGene00012233 | proteolysis |
| WBGene00017553 | proteolysis |
| WBGene00004964 | pseudopodium organization |
| WBGene00019151 | purine nucleotide binding |
| WBGene00001839 | pyridoxal phosphate binding |
| WBGene00006895 | receptor-mediated endocytosis |
| WBGene00008433 | receptor-mediated endocytosis |
| WBGene00011129 | receptor-mediated endocytosis |
| WBGene00017853 | receptor-mediated endocytosis |
| WBGene00017902 | receptor-mediated endocytosis |
| WBGene00001185 | regulation of cell migration |
| WBGene00003185 | regulation of growth rate |
| WBGene00001185 | regulation of oviposition |
| WBGene00006704 | regulation of protein metabolic process |
| WBGene00006718 | regulation of protein metabolic process |
| WBGene00006719 | regulation of protein metabolic process |
| WBGene00044989 | regulation of Rho protein signal transduction |
| WBGene00000584 | regulation of transcription |
| WBGene00015934 | regulation of transcription |
| WBGene00000222 | regulation of transcription, DNA-dependent |
| WBGene00000584 | regulation of transcription, DNA-dependent |
| WBGene00001249 | regulation of transcription, DNA-dependent |
| WBGene00015934 | regulation of transcription, DNA-dependent |
| WBGene00000248 | reproduction |
| WBGene00000584 | reproduction |
| WBGene00000865 | reproduction |
| WBGene00002879 | reproduction |
| WBGene00003055 | reproduction |
| WBGene00003185 | reproduction |
| WBGene00003443 | reproduction |
| WBGene00004949 | reproduction |
| WBGene00005019 | reproduction |
| WBGene00006039 | reproduction |
| WBGene00006895 | reproduction |
| WBGene00008266 | reproduction |
| WBGene00008854 | reproduction |
| WBGene00009016 | reproduction |
| WBGene00009039 | reproduction |
| WBGene00009308 | reproduction |
| WBGene00009442 | reproduction |
| WBGene00010623 | reproduction |
| WBGene00010636 | reproduction |
| WBGene00010682 | reproduction |
| WBGene00010719 | reproduction |
| WBGene00011176 | reproduction |
| WBGene00013011 | reproduction |
| WBGene00017853 | reproduction |
| WBGene00018999 | reproduction |
| WBGene00019151 | reproduction |
| WBGene00021880 | reproduction |
| WBGene00003185 | response to cadmium ion |
| WBGene00003185 | response to copper ion |
| WBGene00015093 | response to heat |
| WBGene00015094 | response to heat |
| WBGene00003185 | response to starvation |
| WBGene00003185 | response to starvation |
| WBGene00003185 | response to temperature stimulus |
| WBGene00044989 | Rho guanyl-nucleotide exchange factor activity |
| WBGene00011533 | ribosome |
| WBGene00015084 | ribosome |
| WBGene00008433 | RNA interference |
| WBGene00011129 | secretion by cell |
| WBGene00009238 | selenium binding |
| WBGene00000222 | sequence-specific DNA binding |
| WBGene00000584 | sequence-specific DNA binding |
| WBGene00001249 | sequence-specific DNA binding |
| WBGene00001249 | sequence-specific DNA binding |
| WBGene00014240 | sequence-specific DNA binding |
| WBGene00015934 | sequence-specific DNA binding |
| WBGene00006624 | serine-type endopeptidase activity |
| WBGene00006625 | serine-type endopeptidase activity |
| WBGene00003185 | serine-type endopeptidase inhibitor activity |
| WBGene00008854 | serine-type endopeptidase inhibitor activity |
| WBGene00013303 | serine-type endopeptidase inhibitor activity |
| WBGene00019812 | serine-type endopeptidase inhibitor activity |
| WBGene00001709 | signal transducer activity |
| WBGene00013011 | signal transducer activity |
| WBGene00001709 | signal transduction |
| WBGene00006704 | small conjugating protein ligase activity |
| WBGene00006718 | small conjugating protein ligase activity |
| WBGene00006719 | small conjugating protein ligase activity |
| WBGene00001249 | specific RNA polymerase II transcription factor activity |
| WBGene00004964 | sperm motility |
| WBGene00004964 | spermatid development |
| WBGene00004971 | spermatid development |
| WBGene00004973 | spermatid development |
| WBGene00001249 | spermatogenesis |
| WBGene00000383 | spindle |
| WBGene00005019 | steroid biosynthetic process |
| WBGene00011533 | structural constituent of ribosome |
| WBGene00015084 | structural constituent of ribosome |
| WBGene00013290 | structural constituent of vitelline membrane |
| WBGene00021457 | structural constituent of vitelline membrane |
| WBGene00000248 | **structural molecule activity** |
| WBGene00000248 | **structural molecule activity** |
| WBGene00002052 | **structural molecule activity** |
| WBGene00003424 | **structural molecule activity** |
| WBGene00003426 | **structural molecule activity** |
| WBGene00003429 | **structural molecule activity** |
| WBGene00003430 | **structural molecule activity** |
| WBGene00003431 | **structural molecule activity** |
| WBGene00003435 | **structural molecule activity** |
| WBGene00003438 | **structural molecule activity** |
| WBGene00003442 | **structural molecule activity** |
| WBGene00003443 | **structural molecule activity** |
| WBGene00003444 | **structural molecule activity** |
| WBGene00003446 | **structural molecule activity** |
| WBGene00003452 | **structural molecule activity** |
| WBGene00003456 | **structural molecule activity** |
| WBGene00003457 | **structural molecule activity** |
| WBGene00003468 | **structural molecule activity** |
| WBGene00003469 | **structural molecule activity** |
| WBGene00003470 | **structural molecule activity** |
| WBGene00006039 | **structural molecule activity** |
| WBGene00006047 | **structural molecule activity** |
| WBGene00007721 | **structural molecule activity** |
| WBGene00007948 | **structural molecule activity** |
| WBGene00007987 | **structural molecule activity** |
| WBGene00008724 | **structural molecule activity** |
| WBGene00009031 | **structural molecule activity** |
| WBGene00009470 | **structural molecule activity** |
| WBGene00009550 | **structural molecule activity** |
| WBGene00010184 | **structural molecule activity** |
| WBGene00010254 | **structural molecule activity** |
| WBGene00011478 | **structural molecule activity** |
| WBGene00011911 | **structural molecule activity** |
| WBGene00013165 | **structural molecule activity** |
| WBGene00013773 | **structural molecule activity** |
| WBGene00014247 | **structural molecule activity** |
| WBGene00016399 | **structural molecule activity** |
| WBGene00018119 | **structural molecule activity** |
| WBGene00018336 | **structural molecule activity** |
| WBGene00019431 | **structural molecule activity** |
| WBGene00021428 | **structural molecule activity** |
| WBGene00022002 | **structural molecule activity** |
| WBGene00022760 | **structural molecule activity** |
| WBGene00017195 | synapse |
| WBGene00001159 | syncytium formation by plasma membrane fusion |
| WBGene00001159 | syncytium formation by plasma membrane fusion |
| WBGene00001159 | syncytium formation by plasma membrane fusion |
| WBGene00001159 | tail morphogenesis |
| WBGene00001159 | tail tip morphogenesis |
| WBGene00000222 | transcription factor activity |
| WBGene00000584 | transcription factor activity |
| WBGene00001249 | transcription factor activity |
| WBGene00001249 | transcription factor activity |
| WBGene00015934 | transcription factor activity |
| WBGene00000584 | transcription regulator activity |
| WBGene00000584 | transcription repressor binding |
| WBGene00016753 | transferase activity, transferring acyl groups other than amino-acyl groups |
| WBGene00003055 | transforming growth factor beta receptor signaling pathway |
| WBGene00011533 | translation |
| WBGene00015084 | translation |
| WBGene00015093 | translation |
| WBGene00015094 | translation |
| WBGene00006487 | transmembrane transport |
| WBGene00007549 | transmembrane transport |
| WBGene00008137 | transmembrane transport |
| WBGene00009513 | transmembrane transport |
| WBGene00012786 | transmembrane transport |
| WBGene00012811 | transmembrane transport |
| WBGene00013318 | transmembrane transport |
| WBGene00007549 | transport |
| WBGene00012786 | transport |
| WBGene00013318 | transport |
| WBGene00007549 | transporter activity |
| WBGene00013318 | transporter activity |
| WBGene00015422 | ubiquitin thiolesterase activity |
| WBGene00007633 | ubiquitin-dependent protein catabolic process |
| WBGene00015422 | ubiquitin-dependent protein catabolic process |
| WBGene00001045 | unfolded protein binding |
| WBGene00004899 | vesicle-mediated transport |
| WBGene00044062 | vesicle-mediated transport |
| WBGene00013290 | vitelline membrane formation |
| WBGene00021457 | vitelline membrane formation |
| WBGene00001159 | vulval development |
| WBGene00005019 | vulval development |
| WBGene00001178 | zinc ion binding |
| WBGene00001249 | zinc ion binding |
| WBGene00004964 | zinc ion binding |
| WBGene00005013 | zinc ion binding |
| WBGene00006895 | zinc ion binding |
| WBGene00007666 | zinc ion binding |
| WBGene00007740 | zinc ion binding |
| WBGene00008266 | zinc ion binding |
| WBGene00008433 | zinc ion binding |
| WBGene00008606 | zinc ion binding |
| WBGene00012379 | zinc ion binding |
| WBGene00012873 | zinc ion binding |
| WBGene00016320 | zinc ion binding |
| WBGene00020066 | zinc ion binding |
| **^a^**Over-represented terms shown in **bold.** | |
