# Supplementary material for: SPE-44 Implements Sperm Cell Fate
Source: PLoS Genet. 2012 Apr 26;8(4):e1002678. doi: 10.1371/journal.pgen.1002678 (PMC3343087; doi:10.1371/journal.pgen.1002678)
Supplement: Table S4 — GO terms associated with spe-44 up-regulated genes. Listed are the genes from Table S2 with increased expression in the spe-44 mutant strain, and all of the Gene Ontology terms associated with those genes. (DOC) [file pgen.1002678.s008.doc]

| **Table S4. GO terms associated with genes up-regulated in *spe-44*** | |
| --- | --- |
| **Wormbase Gene Identifier** | **GO term^a^** |
| WBGene00012608 | 3-dehydroquinate synthase activity |
| WBGene00014220 | 3'-5' exonuclease activity |
| WBGene00011262 | acid phosphatase activity |
| WBGene00019792 | actin binding |
| WBGene00000959 | activation of protein kinase C activity by G-protein coupled receptor protein signaling pathway |
| WBGene00000070 | adenylate cyclase activity |
| WBGene00000196 | alanine-tRNA ligase activity |
| WBGene00000196 | alanyl-tRNA aminoacylation |
| WBGene00006935 | aminoacyl-tRNA ligase activity |
| WBGene00003134 | anaphase-promoting complex |
| WBGene00001170 | apoptosis |
| WBGene00001204 | apoptosis |
| WBGene00001170 | apoptotic mitochondrial changes |
| WBGene00001170 | apoptotic mitochondrial changes |
| WBGene00012608 | aromatic amino acid family biosynthetic process |
| WBGene00000196 | **ATP binding** |
| WBGene00000377 | **ATP binding** |
| WBGene00000378 | **ATP binding** |
| WBGene00001532 | **ATP binding** |
| WBGene00001740 | **ATP binding** |
| WBGene00002025 | **ATP binding** |
| WBGene00002035 | **ATP binding** |
| WBGene00003421 | **ATP binding** |
| WBGene00004062 | **ATP binding** |
| WBGene00004391 | **ATP binding** |
| WBGene00004859 | **ATP binding** |
| WBGene00006436 | **ATP binding** |
| WBGene00006437 | **ATP binding** |
| WBGene00006779 | **ATP binding** |
| WBGene00006814 | **ATP binding** |
| WBGene00006935 | **ATP binding** |
| WBGene00007513 | **ATP binding** |
| WBGene00008119 | **ATP binding** |
| WBGene00009124 | **ATP binding** |
| WBGene00010015 | **ATP binding** |
| WBGene00010562 | **ATP binding** |
| WBGene00010839 | **ATP binding** |
| WBGene00011893 | **ATP binding** |
| WBGene00012352 | **ATP binding** |
| WBGene00012785 | **ATP binding** |
| WBGene00013124 | **ATP binding** |
| WBGene00013978 | **ATP binding** |
| WBGene00020964 | **ATP binding** |
| WBGene00022635 | **ATP binding** |
| WBGene00044916 | **ATP binding** |
| WBGene00009124 | ATP-dependent DNA helicase activity |
| WBGene00010839 | ATP-dependent DNA helicase activity |
| WBGene00008119 | ATP-dependent helicase activity |
| WBGene00009124 | ATP-dependent helicase activity |
| WBGene00010839 | ATP-dependent helicase activity |
| WBGene00020964 | ATP-dependent helicase activity |
| WBGene00004062 | ATPase activity |
| WBGene00007513 | ATPase activity |
| WBGene00010562 | ATPase activity |
| WBGene00044916 | ATPase activity |
| WBGene00006562 | axon |
| WBGene00012352 | axon |
| WBGene00001328 | axonal defasciculation |
| WBGene00000998 | axonal fasciculation |
| WBGene00006349 | axonal fasciculation |
| WBGene00013720 | axonal fasciculation |
| WBGene00022635 | axonal fasciculation |
| WBGene00003738 | basal part of cell |
| WBGene00001263 | basement membrane |
| WBGene00001328 | basement membrane |
| WBGene00001328 | basement membrane |
| WBGene00002280 | basement membrane |
| WBGene00003738 | basement membrane |
| WBGene00003134 | binding |
| WBGene00007709 | binding |
| WBGene00008594 | binding |
| WBGene00009114 | binding |
| WBGene00009395 | binding |
| WBGene00011350 | binding |
| WBGene00014114 | binding |
| WBGene00016966 | binding |
| WBGene00019819 | binding |
| WBGene00020068 | binding |
| WBGene00022257 | binding |
| WBGene00001740 | biosynthetic process |
| WBGene00000871 | body morphogenesis |
| WBGene00001328 | body morphogenesis |
| WBGene00001974 | body morphogenesis |
| WBGene00002055 | body morphogenesis |
| WBGene00002497 | body morphogenesis |
| WBGene00003026 | body morphogenesis |
| WBGene00003134 | body morphogenesis |
| WBGene00003882 | body morphogenesis |
| WBGene00006437 | body morphogenesis |
| WBGene00007013 | body morphogenesis |
| WBGene00007513 | body morphogenesis |
| WBGene00011908 | body morphogenesis |
| WBGene00000397 | calcium ion binding |
| WBGene00000998 | calcium ion binding |
| WBGene00003738 | calcium ion binding |
| WBGene00008081 | calcium ion binding |
| WBGene00008779 | calcium ion binding |
| WBGene00020859 | calcium ion binding |
| WBGene00006779 | calmodulin binding |
| WBGene00006779 | calmodulin-dependent protein kinase activity |
| WBGene00007422 | carbohydrate binding |
| WBGene00004052 | carbohydrate metabolic process |
| WBGene00007422 | carbohydrate metabolic process |
| WBGene00011147 | carbohydrate metabolic process |
| WBGene00011923 | carbohydrate metabolic process |
| WBGene00017771 | carbon-sulfur lyase activity |
| WBGene00006562 | carboxy-lyase activity |
| WBGene00006562 | carboxylic acid metabolic process |
| WBGene00006562 | catalytic activity |
| WBGene00010055 | catalytic activity |
| WBGene00011350 | catalytic activity |
| WBGene00011893 | catalytic activity |
| WBGene00011923 | catalytic activity |
| WBGene00017316 | catalytic activity |
| WBGene00019819 | catalytic activity |
| WBGene00020256 | catalytic activity |
| WBGene00001651 | cation transmembrane transporter activity |
| WBGene00001651 | cation transport |
| WBGene00000397 | cell adhesion |
| WBGene00001328 | cell adhesion |
| WBGene00002368 | cell cortex |
| WBGene00012352 | cell cortex |
| WBGene00019792 | cell cycle arrest |
| WBGene00000871 | cell division |
| WBGene00001974 | cell division |
| WBGene00010015 | cell division |
| WBGene00022257 | cell division |
| WBGene00001204 | cell fate commitment |
| WBGene00001483 | cell fate specification |
| WBGene00001328 | cell migration |
| WBGene00001996 | cell proliferation |
| WBGene00006562 | cell soma |
| WBGene00012352 | cell soma |
| WBGene00003480 | cell-matrix adhesion |
| WBGene00003738 | cell-matrix adhesion |
| WBGene00006562 | cellular amino acid and derivative metabolic process |
| WBGene00000377 | cellular protein metabolic process |
| WBGene00000378 | cellular protein metabolic process |
| WBGene00002025 | cellular protein metabolic process |
| WBGene00003230 | centrosome |
| WBGene00003421 | chiasma formation |
| WBGene00000465 | chitin binding |
| WBGene00000465 | chitin metabolic process |
| WBGene00001996 | chromatin |
| WBGene00001996 | chromatin assembly or disassembly |
| WBGene00001874 | chromatin binding |
| WBGene00001996 | chromatin binding |
| WBGene00010988 | cobalamin binding |
| WBGene00001263 | collagen |
| WBGene00002280 | collagen |
| WBGene00002497 | collagen biosynthetic process |
| WBGene00001831 | condensed chromosome, centromeric region |
| WBGene00020316 | condensed nuclear chromosome |
| WBGene00000998 | copper ion binding |
| WBGene00020556 | copper ion binding |
| WBGene00000070 | cyclic nucleotide biosynthetic process |
| WBGene00001532 | cyclic nucleotide biosynthetic process |
| WBGene00000871 | cyclin-dependent protein kinase holoenzyme complex |
| WBGene00000871 | cyclin-dependent protein kinase regulator activity |
| WBGene00002035 | cysteine-type endopeptidase activity |
| WBGene00013893 | cytochrome-c oxidase activity |
| WBGene00000871 | cytokinesis |
| WBGene00001974 | cytokinesis |
| WBGene00002047 | cytokinesis |
| WBGene00009163 | cytokinesis |
| WBGene00010015 | cytokinesis |
| WBGene00022257 | cytokinesis |
| WBGene00000196 | cytoplasm |
| WBGene00000377 | cytoplasm |
| WBGene00000378 | cytoplasm |
| WBGene00000998 | cytoplasm |
| WBGene00001481 | cytoplasm |
| WBGene00001595 | cytoplasm |
| WBGene00002025 | cytoplasm |
| WBGene00003026 | cytoplasm |
| WBGene00003230 | cytoplasm |
| WBGene00006935 | cytoplasm |
| WBGene00012352 | cytoplasm |
| WBGene00001170 | cytosol |
| WBGene00001595 | cytosol |
| WBGene00004859 | dauer larval development |
| WBGene00001204 | defecation |
| WBGene00006349 | defecation |
| WBGene00006349 | defense response |
| WBGene00006779 | defense response |
| WBGene00012352 | dendrite |
| WBGene00007302 | dephosphorylation |
| WBGene00000250 | determination of adult life span |
| WBGene00001263 | determination of adult life span |
| WBGene00001740 | determination of adult life span |
| WBGene00001740 | determination of adult life span |
| WBGene00002130 | determination of adult life span |
| WBGene00003026 | determination of adult life span |
| WBGene00003789 | determination of adult life span |
| WBGene00006349 | determination of adult life span |
| WBGene00006474 | determination of adult life span |
| WBGene00007000 | determination of adult life span |
| WBGene00008143 | determination of adult life span |
| WBGene00011831 | determination of adult life span |
| WBGene00017319 | determination of adult life span |
| WBGene00044916 | determination of adult life span |
| WBGene00000959 | diacylglycerol kinase activity |
| WBGene00001740 | dimethylallyltranstransferase activity |
| WBGene00001204 | DNA binding |
| WBGene00001310 | DNA binding |
| WBGene00001831 | DNA binding |
| WBGene00001974 | DNA binding |
| WBGene00009124 | DNA binding |
| WBGene00010839 | DNA binding |
| WBGene00020964 | DNA binding |
| WBGene00000871 | DNA endoreduplication |
| WBGene00003882 | DNA replication |
| WBGene00004391 | DNA replication |
| WBGene00020964 | DNA replication |
| WBGene00020964 | DNA-directed DNA polymerase activity |
| WBGene00020316 | double-strand break repair |
| WBGene00020316 | double-strand break repair |
| WBGene00020316 | double-strand break repair via homologous recombination |
| WBGene00020316 | double-strand break repair via single-strand annealing |
| WBGene00009163 | double-stranded RNA binding |
| WBGene00018857 | double-stranded RNA binding |
| WBGene00011696 | early endosome |
| WBGene00000998 | electron carrier activity |
| WBGene00001263 | embryonic development |
| WBGene00001328 | embryonic development |
| WBGene00007972 | embryonic development |
| WBGene00000250 | embryonic development ending in birth or egg hatching |
| WBGene00000377 | embryonic development ending in birth or egg hatching |
| WBGene00000378 | embryonic development ending in birth or egg hatching |
| WBGene00000465 | embryonic development ending in birth or egg hatching |
| WBGene00000871 | embryonic development ending in birth or egg hatching |
| WBGene00001263 | embryonic development ending in birth or egg hatching |
| WBGene00001310 | embryonic development ending in birth or egg hatching |
| WBGene00001595 | embryonic development ending in birth or egg hatching |
| WBGene00001831 | embryonic development ending in birth or egg hatching |
| WBGene00001874 | embryonic development ending in birth or egg hatching |
| WBGene00001974 | embryonic development ending in birth or egg hatching |
| WBGene00002025 | embryonic development ending in birth or egg hatching |
| WBGene00002047 | embryonic development ending in birth or egg hatching |
| WBGene00002055 | embryonic development ending in birth or egg hatching |
| WBGene00002130 | embryonic development ending in birth or egg hatching |
| WBGene00002280 | embryonic development ending in birth or egg hatching |
| WBGene00002368 | embryonic development ending in birth or egg hatching |
| WBGene00002497 | embryonic development ending in birth or egg hatching |
| WBGene00003134 | embryonic development ending in birth or egg hatching |
| WBGene00003230 | embryonic development ending in birth or egg hatching |
| WBGene00003421 | embryonic development ending in birth or egg hatching |
| WBGene00003499 | embryonic development ending in birth or egg hatching |
| WBGene00003789 | embryonic development ending in birth or egg hatching |
| WBGene00003798 | embryonic development ending in birth or egg hatching |
| WBGene00003882 | embryonic development ending in birth or egg hatching |
| WBGene00004391 | embryonic development ending in birth or egg hatching |
| WBGene00004723 | embryonic development ending in birth or egg hatching |
| WBGene00006307 | embryonic development ending in birth or egg hatching |
| WBGene00006349 | embryonic development ending in birth or egg hatching |
| WBGene00006436 | embryonic development ending in birth or egg hatching |
| WBGene00006481 | embryonic development ending in birth or egg hatching |
| WBGene00006571 | embryonic development ending in birth or egg hatching |
| WBGene00006935 | embryonic development ending in birth or egg hatching |
| WBGene00007000 | embryonic development ending in birth or egg hatching |
| WBGene00007013 | embryonic development ending in birth or egg hatching |
| WBGene00007564 | embryonic development ending in birth or egg hatching |
| WBGene00007972 | embryonic development ending in birth or egg hatching |
| WBGene00008143 | embryonic development ending in birth or egg hatching |
| WBGene00008921 | embryonic development ending in birth or egg hatching |
| WBGene00009163 | embryonic development ending in birth or egg hatching |
| WBGene00009668 | embryonic development ending in birth or egg hatching |
| WBGene00010013 | embryonic development ending in birth or egg hatching |
| WBGene00010015 | embryonic development ending in birth or egg hatching |
| WBGene00010139 | embryonic development ending in birth or egg hatching |
| WBGene00010562 | embryonic development ending in birth or egg hatching |
| WBGene00010621 | embryonic development ending in birth or egg hatching |
| WBGene00010839 | embryonic development ending in birth or egg hatching |
| WBGene00011350 | embryonic development ending in birth or egg hatching |
| WBGene00011696 | embryonic development ending in birth or egg hatching |
| WBGene00011908 | embryonic development ending in birth or egg hatching |
| WBGene00012460 | embryonic development ending in birth or egg hatching |
| WBGene00013978 | embryonic development ending in birth or egg hatching |
| WBGene00016142 | embryonic development ending in birth or egg hatching |
| WBGene00016444 | embryonic development ending in birth or egg hatching |
| WBGene00016816 | embryonic development ending in birth or egg hatching |
| WBGene00016966 | embryonic development ending in birth or egg hatching |
| WBGene00017319 | embryonic development ending in birth or egg hatching |
| WBGene00017627 | embryonic development ending in birth or egg hatching |
| WBGene00018064 | embryonic development ending in birth or egg hatching |
| WBGene00018857 | embryonic development ending in birth or egg hatching |
| WBGene00019353 | embryonic development ending in birth or egg hatching |
| WBGene00020068 | embryonic development ending in birth or egg hatching |
| WBGene00020316 | embryonic development ending in birth or egg hatching |
| WBGene00022257 | embryonic development ending in birth or egg hatching |
| WBGene00011696 | endocytic vesicle |
| WBGene00011696 | endocytosis |
| WBGene00015189 | endodeoxyribonuclease activity, producing 5'-phosphomonoesters |
| WBGene00001310 | endodermal cell fate determination |
| WBGene00002497 | endoplasmic reticulum |
| WBGene00003789 | establishment of mitotic spindle orientation |
| WBGene00001328 | extracellular matrix |
| WBGene00001263 | extracellular matrix organization |
| WBGene00001263 | extracellular matrix structural constituent |
| WBGene00001263 | extracellular matrix structural constituent |
| WBGene00002280 | extracellular matrix structural constituent |
| WBGene00003738 | extracellular matrix structural constituent |
| WBGene00000465 | extracellular region |
| WBGene00008081 | extracellular region |
| WBGene00009891 | extracellular region |
| WBGene00021780 | extracellular region |
| WBGene00010055 | FAD binding |
| WBGene00001595 | feminization of hermaphroditic germ-line |
| WBGene00018138 | folic acid binding |
| WBGene00021275 | G-protein coupled receptor protein signaling pathway |
| WBGene00000070 | G-protein signaling, coupled to cAMP nucleotide second messenger |
| WBGene00000871 | G1/S transition of mitotic cell cycle |
| WBGene00000250 | gamete generation |
| WBGene00000871 | gamete generation |
| WBGene00001974 | gamete generation |
| WBGene00003026 | gamete generation |
| WBGene00002130 | gap junction |
| WBGene00007972 | gastrulation |
| WBGene00007972 | gastrulation with mouth forming first |
| WBGene00000871 | germ cell development |
| WBGene00001996 | germ cell development |
| WBGene00001996 | germ cell development |
| WBGene00001481 | germ-line sex determination |
| WBGene00001481 | germline cell cycle switching, mitotic to meiotic cell cycle |
| WBGene00001595 | germline cell cycle switching, mitotic to meiotic cell cycle |
| WBGene00000871 | gonad development |
| WBGene00000998 | gonad development |
| WBGene00001996 | gonad development |
| WBGene00001996 | gonad development |
| WBGene00000377 | growth |
| WBGene00000378 | growth |
| WBGene00000871 | growth |
| WBGene00001263 | growth |
| WBGene00001310 | growth |
| WBGene00001328 | growth |
| WBGene00001740 | growth |
| WBGene00001974 | growth |
| WBGene00002055 | growth |
| WBGene00002280 | growth |
| WBGene00002368 | growth |
| WBGene00003230 | growth |
| WBGene00003789 | growth |
| WBGene00003798 | growth |
| WBGene00004391 | growth |
| WBGene00004859 | growth |
| WBGene00006474 | growth |
| WBGene00006481 | growth |
| WBGene00006935 | growth |
| WBGene00007000 | growth |
| WBGene00007013 | growth |
| WBGene00007564 | growth |
| WBGene00007972 | growth |
| WBGene00008143 | growth |
| WBGene00009668 | growth |
| WBGene00010015 | growth |
| WBGene00010562 | growth |
| WBGene00011831 | growth |
| WBGene00011908 | growth |
| WBGene00016142 | growth |
| WBGene00017319 | growth |
| WBGene00017627 | growth |
| WBGene00019353 | growth |
| WBGene00022881 | growth |
| WBGene00007000 | GTP binding |
| WBGene00013124 | GTP binding |
| WBGene00021027 | GTP binding |
| WBGene00007000 | GTPase activity |
| WBGene00019832 | guanyl-nucleotide exchange factor activity |
| WBGene00001263 | hatching |
| WBGene00002280 | hatching |
| WBGene00008119 | helicase activity |
| WBGene00020964 | helicase activity |
| WBGene00016700 | heme binding |
| WBGene00000377 | hermaphrodite genitalia development |
| WBGene00000378 | hermaphrodite genitalia development |
| WBGene00000871 | hermaphrodite genitalia development |
| WBGene00000998 | hermaphrodite genitalia development |
| WBGene00001310 | hermaphrodite genitalia development |
| WBGene00001651 | hermaphrodite genitalia development |
| WBGene00001831 | hermaphrodite genitalia development |
| WBGene00001974 | hermaphrodite genitalia development |
| WBGene00003134 | hermaphrodite genitalia development |
| WBGene00003882 | hermaphrodite genitalia development |
| WBGene00004391 | hermaphrodite genitalia development |
| WBGene00004723 | hermaphrodite genitalia development |
| WBGene00007972 | hermaphrodite genitalia development |
| WBGene00009668 | hermaphrodite genitalia development |
| WBGene00011908 | hermaphrodite genitalia development |
| WBGene00016700 | hermaphrodite genitalia development |
| WBGene00017319 | hermaphrodite genitalia development |
| WBGene00019353 | hermaphrodite genitalia development |
| WBGene00022881 | hermaphrodite genitalia development |
| WBGene00011908 | hermaphrodite genitalia morphogenesis |
| WBGene00006474 | histone H3-K4 methylation |
| WBGene00010988 | homocysteine S-methyltransferase activity |
| WBGene00003421 | homologous chromosome segregation |
| WBGene00000397 | homophilic cell adhesion |
| WBGene00011923 | hyalurononglucosaminidase activity |
| WBGene00008497 | hydrolase activity |
| WBGene00009124 | hydrolase activity, acting on acid anhydrides, in phosphorus-containing anhydrides |
| WBGene00010839 | hydrolase activity, acting on acid anhydrides, in phosphorus-containing anhydrides |
| WBGene00000377 | inductive cell migration |
| WBGene00000998 | inductive cell migration |
| WBGene00001263 | inductive cell migration |
| WBGene00002280 | inductive cell migration |
| WBGene00000998 | inorganic diphosphatase activity |
| WBGene00007620 | inositol or phosphatidylinositol phosphatase activity |
| WBGene00000070 | integral to membrane |
| WBGene00000397 | integral to membrane |
| WBGene00000634 | integral to membrane |
| WBGene00000687 | integral to membrane |
| WBGene00000722 | integral to membrane |
| WBGene00000753 | integral to membrane |
| WBGene00000959 | integral to membrane |
| WBGene00001532 | integral to membrane |
| WBGene00001651 | integral to membrane |
| WBGene00002130 | integral to membrane |
| WBGene00003738 | integral to membrane |
| WBGene00003798 | integral to membrane |
| WBGene00004062 | integral to membrane |
| WBGene00004157 | integral to membrane |
| WBGene00005080 | integral to membrane |
| WBGene00005143 | integral to membrane |
| WBGene00005681 | integral to membrane |
| WBGene00006206 | integral to membrane |
| WBGene00007347 | integral to membrane |
| WBGene00007422 | integral to membrane |
| WBGene00007513 | integral to membrane |
| WBGene00008171 | integral to membrane |
| WBGene00009114 | integral to membrane |
| WBGene00009779 | integral to membrane |
| WBGene00010139 | integral to membrane |
| WBGene00010273 | integral to membrane |
| WBGene00010334 | integral to membrane |
| WBGene00010467 | integral to membrane |
| WBGene00010621 | integral to membrane |
| WBGene00011350 | integral to membrane |
| WBGene00011893 | integral to membrane |
| WBGene00012555 | integral to membrane |
| WBGene00012606 | integral to membrane |
| WBGene00013720 | integral to membrane |
| WBGene00016402 | integral to membrane |
| WBGene00016441 | integral to membrane |
| WBGene00016499 | integral to membrane |
| WBGene00016659 | integral to membrane |
| WBGene00017236 | integral to membrane |
| WBGene00017271 | integral to membrane |
| WBGene00017316 | integral to membrane |
| WBGene00017804 | integral to membrane |
| WBGene00017871 | integral to membrane |
| WBGene00017876 | integral to membrane |
| WBGene00017978 | integral to membrane |
| WBGene00018138 | integral to membrane |
| WBGene00018295 | integral to membrane |
| WBGene00018497 | integral to membrane |
| WBGene00018500 | integral to membrane |
| WBGene00018507 | integral to membrane |
| WBGene00019345 | integral to membrane |
| WBGene00019437 | integral to membrane |
| WBGene00019518 | integral to membrane |
| WBGene00019811 | integral to membrane |
| WBGene00020160 | integral to membrane |
| WBGene00020207 | integral to membrane |
| WBGene00020256 | integral to membrane |
| WBGene00020415 | integral to membrane |
| WBGene00020556 | integral to membrane |
| WBGene00021275 | integral to membrane |
| WBGene00021967 | integral to membrane |
| WBGene00194818 | integral to membrane |
| WBGene00000070 | **intracellular** |
| WBGene00000250 | **intracellular** |
| WBGene00000959 | **intracellular** |
| WBGene00001481 | **intracellular** |
| WBGene00001532 | **intracellular** |
| WBGene00001740 | **intracellular** |
| WBGene00002368 | **intracellular** |
| WBGene00003026 | **intracellular** |
| WBGene00003480 | **intracellular** |
| WBGene00006307 | **intracellular** |
| WBGene00006437 | **intracellular** |
| WBGene00006445 | **intracellular** |
| WBGene00007000 | **intracellular** |
| WBGene00007620 | **intracellular** |
| WBGene00007772 | **intracellular** |
| WBGene00009163 | **intracellular** |
| WBGene00010776 | **intracellular** |
| WBGene00010988 | **intracellular** |
| WBGene00011696 | **intracellular** |
| WBGene00012352 | **intracellular** |
| WBGene00013124 | **intracellular** |
| WBGene00014220 | **intracellular** |
| WBGene00014256 | **intracellular** |
| WBGene00016142 | **intracellular** |
| WBGene00017319 | **intracellular** |
| WBGene00018857 | **intracellular** |
| WBGene00019832 | **intracellular** |
| WBGene00019832 | **intracellular** |
| WBGene00021027 | **intracellular** |
| WBGene00001170 | intracellular membrane-bounded organelle |
| WBGene00001651 | ion channel activity |
| WBGene00001651 | ion transport |
| WBGene00002497 | iron ion binding |
| WBGene00011893 | kinase activity |
| WBGene00002047 | kinetochore |
| WBGene00001831 | kinetochore assembly |
| WBGene00002497 | L-ascorbic acid binding |
| WBGene00007025 | lateral inhibition |
| WBGene00000196 | ligase activity, forming aminoacyl-tRNA and related compounds |
| WBGene00007422 | lipid glycosylation |
| WBGene00019520 | lipid metabolic process |
| WBGene00000070 | lipid storage |
| WBGene00000511 | lipid storage |
| WBGene00002130 | lipid storage |
| WBGene00002184 | lipid storage |
| WBGene00010176 | lipid storage |
| WBGene00016966 | lipid storage |
| WBGene00018840 | lipid storage |
| WBGene00019811 | lipid storage |
| WBGene00020160 | lipid storage |
| WBGene00006814 | lipopolysaccharide biosynthetic process |
| WBGene00000871 | locomotion |
| WBGene00001263 | locomotion |
| WBGene00001310 | locomotion |
| WBGene00001328 | locomotion |
| WBGene00001974 | locomotion |
| WBGene00002280 | locomotion |
| WBGene00003026 | locomotion |
| WBGene00003480 | locomotion |
| WBGene00003789 | locomotion |
| WBGene00003882 | locomotion |
| WBGene00004391 | locomotion |
| WBGene00004723 | locomotion |
| WBGene00006206 | locomotion |
| WBGene00006349 | locomotion |
| WBGene00006437 | locomotion |
| WBGene00006481 | locomotion |
| WBGene00006779 | locomotion |
| WBGene00007972 | locomotion |
| WBGene00008143 | locomotion |
| WBGene00009366 | locomotion |
| WBGene00009395 | locomotion |
| WBGene00009668 | locomotion |
| WBGene00011831 | locomotion |
| WBGene00011908 | locomotion |
| WBGene00012352 | locomotion |
| WBGene00020085 | locomotion |
| WBGene00000998 | magnesium ion binding |
| WBGene00001481 | masculinization of hermaphroditic germ-line |
| WBGene00001483 | masculinization of hermaphroditic germ-line |
| WBGene00001595 | masculinization of hermaphroditic germ-line |
| WBGene00016960 | masculinization of hermaphroditic germ-line |
| WBGene00001874 | meiosis |
| WBGene00003134 | meiosis |
| WBGene00018857 | meiosis |
| WBGene00001874 | meiotic chromosome segregation |
| WBGene00003499 | meiotic chromosome segregation |
| WBGene00003804 | meiotic chromosome segregation |
| WBGene00008061 | meiotic chromosome segregation |
| WBGene00008921 | meiotic chromosome segregation |
| WBGene00009395 | meiotic chromosome segregation |
| WBGene00020316 | meiotic DNA recombinase assembly |
| WBGene00006571 | meiotic sister chromatid cohesion |
| WBGene00000397 | membrane |
| WBGene00001651 | membrane |
| WBGene00003738 | membrane |
| WBGene00004062 | membrane |
| WBGene00005681 | membrane |
| WBGene00006814 | membrane |
| WBGene00007513 | membrane |
| WBGene00008779 | membrane |
| WBGene00010334 | membrane |
| WBGene00018138 | membrane |
| WBGene00020207 | membrane |
| WBGene00020556 | membrane |
| WBGene00012352 | membrane fraction |
| WBGene00007422 | metabolic process |
| WBGene00008497 | metabolic process |
| WBGene00011350 | metabolic process |
| WBGene00011923 | metabolic process |
| WBGene00017316 | metabolic process |
| WBGene00017771 | metabolic process |
| WBGene00019819 | metabolic process |
| WBGene00020256 | metabolic process |
| WBGene00010988 | metal ion binding |
| WBGene00012608 | metal ion binding |
| WBGene00009645 | metalloendopeptidase activity |
| WBGene00010988 | methionine biosynthetic process |
| WBGene00010988 | methionine synthase activity |
| WBGene00003421 | mismatch repair |
| WBGene00003421 | mismatched DNA binding |
| WBGene00002025 | mitochondrial unfolded protein response |
| WBGene00001170 | mitochondrion |
| WBGene00002025 | mitochondrion |
| WBGene00013893 | mitochondrion |
| WBGene00002025 | mitochondrion organization |
| WBGene00006779 | mitosis |
| WBGene00010562 | mitosis |
| WBGene00001651 | mitotic cell cycle |
| WBGene00006571 | mitotic cohesin complex |
| WBGene00010562 | mitotic metaphase/anaphase transition |
| WBGene00006571 | mitotic sister chromatid cohesion |
| WBGene00006571 | mitotic sister chromatid cohesion |
| WBGene00001831 | mitotic sister chromatid segregation |
| WBGene00001831 | mitotic spindle organization |
| WBGene00009163 | mitotic spindle organization |
| WBGene00010562 | mitotic spindle organization |
| WBGene00003026 | molting cycle, collagen and cuticulin-based cuticle |
| WBGene00006481 | molting cycle, collagen and cuticulin-based cuticle |
| WBGene00010015 | molting cycle, collagen and cuticulin-based cuticle |
| WBGene00011831 | molting cycle, collagen and cuticulin-based cuticle |
| WBGene00020556 | monooxygenase activity |
| WBGene00001831 | morphogenesis of an epithelium |
| WBGene00002497 | morphogenesis of an epithelium |
| WBGene00003026 | morphogenesis of an epithelium |
| WBGene00003134 | morphogenesis of an epithelium |
| WBGene00003480 | morphogenesis of an epithelium |
| WBGene00003789 | morphogenesis of an epithelium |
| WBGene00006206 | morphogenesis of an epithelium |
| WBGene00007013 | morphogenesis of an epithelium |
| WBGene00009366 | morphogenesis of an epithelium |
| WBGene00011831 | morphogenesis of an epithelium |
| WBGene00011908 | morphogenesis of an epithelium |
| WBGene00002035 | motor activity |
| WBGene00001481 | mRNA 3'-UTR binding |
| WBGene00001595 | mRNA 3'-UTR binding |
| WBGene00003230 | mRNA 3'-UTR binding |
| WBGene00006307 | mRNA processing |
| WBGene00001263 | muscle organ development |
| WBGene00002035 | myosin complex |
| WBGene00004053 | NAD+ ADP-ribosyltransferase activity |
| WBGene00001170 | negative regulation of anti-apoptosis |
| WBGene00001170 | negative regulation of anti-apoptosis |
| WBGene00001170 | negative regulation of protein binding |
| WBGene00010562 | negative regulation of protein kinase activity |
| WBGene00001996 | negative regulation of Ras protein signal transduction |
| WBGene00001170 | negative regulation of survival gene product expression |
| WBGene00003026 | negative regulation of translation |
| WBGene00003499 | negative regulation of transposition, DNA-mediated |
| WBGene00001328 | negative regulation of vulval development |
| WBGene00001595 | negative regulation of vulval development |
| WBGene00001996 | negative regulation of vulval development |
| WBGene00001996 | negative regulation of vulval development |
| WBGene00003026 | negative regulation of vulval development |
| WBGene00003882 | negative regulation of vulval development |
| WBGene00006349 | negative regulation of vulval development |
| WBGene00006474 | negative regulation of vulval development |
| WBGene00006489 | negative regulation of vulval development |
| WBGene00011908 | negative regulation of vulval development |
| WBGene00014114 | negative regulation of vulval development |
| WBGene00000377 | nematode larval development |
| WBGene00000378 | nematode larval development |
| WBGene00000871 | nematode larval development |
| WBGene00001204 | nematode larval development |
| WBGene00001263 | nematode larval development |
| WBGene00001310 | nematode larval development |
| WBGene00001328 | nematode larval development |
| WBGene00001740 | nematode larval development |
| WBGene00001974 | nematode larval development |
| WBGene00001996 | nematode larval development |
| WBGene00001996 | nematode larval development |
| WBGene00002025 | nematode larval development |
| WBGene00002055 | nematode larval development |
| WBGene00002130 | nematode larval development |
| WBGene00002280 | nematode larval development |
| WBGene00002368 | nematode larval development |
| WBGene00002497 | nematode larval development |
| WBGene00003026 | nematode larval development |
| WBGene00003134 | nematode larval development |
| WBGene00003230 | nematode larval development |
| WBGene00003480 | nematode larval development |
| WBGene00003789 | nematode larval development |
| WBGene00003798 | nematode larval development |
| WBGene00004391 | nematode larval development |
| WBGene00006481 | nematode larval development |
| WBGene00006935 | nematode larval development |
| WBGene00007000 | nematode larval development |
| WBGene00007013 | nematode larval development |
| WBGene00007564 | nematode larval development |
| WBGene00007972 | nematode larval development |
| WBGene00008143 | nematode larval development |
| WBGene00009668 | nematode larval development |
| WBGene00010015 | nematode larval development |
| WBGene00010562 | nematode larval development |
| WBGene00011831 | nematode larval development |
| WBGene00011908 | nematode larval development |
| WBGene00016142 | nematode larval development |
| WBGene00017319 | nematode larval development |
| WBGene00017627 | nematode larval development |
| WBGene00019353 | nematode larval development |
| WBGene00021027 | nematode larval development |
| WBGene00022881 | nematode larval development |
| WBGene00000998 | nervous system development |
| WBGene00001328 | neuron migration |
| WBGene00003738 | neuron migration |
| WBGene00001874 | nuclear chromatin |
| WBGene00003882 | nuclear chromosome |
| WBGene00003798 | nuclear pore |
| WBGene00001481 | nucleic acid binding |
| WBGene00003230 | nucleic acid binding |
| WBGene00003480 | nucleic acid binding |
| WBGene00004723 | nucleic acid binding |
| WBGene00007772 | nucleic acid binding |
| WBGene00008119 | nucleic acid binding |
| WBGene00009124 | nucleic acid binding |
| WBGene00010839 | nucleic acid binding |
| WBGene00014220 | nucleic acid binding |
| WBGene00014256 | nucleic acid binding |
| WBGene00020964 | nucleic acid binding |
| WBGene00021924 | nucleic acid binding |
| WBGene00009124 | nucleobase, nucleoside, nucleotide and nucleic acid metabolic process |
| WBGene00010839 | nucleobase, nucleoside, nucleotide and nucleic acid metabolic process |
| WBGene00014220 | nucleobase, nucleoside, nucleotide and nucleic acid metabolic process |
| WBGene00007513 | nucleoside-triphosphatase activity |
| WBGene00010015 | nucleoside-triphosphatase activity |
| WBGene00010562 | nucleoside-triphosphatase activity |
| WBGene00001831 | nucleosome |
| WBGene00001831 | nucleosome assembly |
| WBGene00000196 | nucleotide binding |
| WBGene00001481 | nucleotide binding |
| WBGene00004723 | nucleotide binding |
| WBGene00006935 | nucleotide binding |
| WBGene00007513 | nucleotide binding |
| WBGene00010015 | nucleotide binding |
| WBGene00010562 | nucleotide binding |
| WBGene00003499 | nucleotidyltransferase activity |
| WBGene00000871 | nucleus |
| WBGene00001204 | nucleus |
| WBGene00001831 | nucleus |
| WBGene00001974 | nucleus |
| WBGene00001996 | nucleus |
| WBGene00001996 | nucleus |
| WBGene00003882 | nucleus |
| WBGene00004053 | nucleus |
| WBGene00006307 | nucleus |
| WBGene00006474 | nucleus |
| WBGene00006571 | nucleus |
| WBGene00007025 | nucleus |
| WBGene00008143 | nucleus |
| WBGene00020316 | nucleus |
| WBGene00043056 | nucleus |
| WBGene00003789 | nucleus organization |
| WBGene00001595 | oogenesis |
| WBGene00001996 | oogenesis |
| WBGene00020068 | oogenesis |
| WBGene00003882 | origin recognition complex |
| WBGene00001170 | oviposition |
| WBGene00001204 | oviposition |
| WBGene00003026 | oviposition |
| WBGene00006349 | oviposition |
| WBGene00009366 | oviposition |
| WBGene00015973 | oviposition |
| WBGene00020316 | oviposition |
| WBGene00002497 | oxidation reduction |
| WBGene00004391 | oxidation reduction |
| WBGene00012608 | oxidation reduction |
| WBGene00016700 | oxidation reduction |
| WBGene00018393 | oxidation reduction |
| WBGene00019819 | oxidation reduction |
| WBGene00020556 | oxidation reduction |
| WBGene00010055 | oxidoreductase activity |
| WBGene00012608 | oxidoreductase activity |
| WBGene00019819 | oxidoreductase activity |
| WBGene00002497 | oxidoreductase activity, acting on paired donors, with incorporation or reduction of molecular oxygen |
| WBGene00018393 | oxidoreductase activity, acting on sulfur group of donors, disulfide as acceptor |
| WBGene00001595 | P granule |
| WBGene00003230 | P granule |
| WBGene00020556 | peptide metabolic process |
| WBGene00012460 | peptidyl-lysine modification to hypusine |
| WBGene00021201 | peptidyl-prolyl cis-trans isomerase activity |
| WBGene00020556 | peptidylglycine monooxygenase activity |
| WBGene00016700 | peroxidase activity |
| WBGene00007302 | phosphatase activity |
| WBGene00000998 | phosphate metabolic process |
| WBGene00011696 | phosphatidylinositol 3-phosphate binding |
| WBGene00011696 | phosphatidylinositol 3-phosphate binding |
| WBGene00000070 | phosphorus-oxygen lyase activity |
| WBGene00001532 | phosphorus-oxygen lyase activity |
| WBGene00011893 | phosphorylation |
| WBGene00006814 | phosphotransferase activity, alcohol group as acceptor |
| WBGene00011147 | phosphotransferase activity, alcohol group as acceptor |
| WBGene00000070 | plasma membrane |
| WBGene00000397 | plasma membrane |
| WBGene00001651 | plasma membrane |
| WBGene00012352 | plasma membrane |
| WBGene00019811 | plasma membrane |
| WBGene00003230 | poly-pyrimidine tract binding |
| WBGene00004052 | poly(ADP-ribose) glycohydrolase activity |
| WBGene00000871 | positive regulation of cell proliferation |
| WBGene00000871 | positive regulation of cyclin-dependent protein kinase activity |
| WBGene00000070 | positive regulation of growth rate |
| WBGene00000196 | positive regulation of growth rate |
| WBGene00000377 | positive regulation of growth rate |
| WBGene00000511 | positive regulation of growth rate |
| WBGene00000871 | positive regulation of growth rate |
| WBGene00001263 | positive regulation of growth rate |
| WBGene00001328 | positive regulation of growth rate |
| WBGene00001483 | positive regulation of growth rate |
| WBGene00001595 | positive regulation of growth rate |
| WBGene00001740 | positive regulation of growth rate |
| WBGene00001974 | positive regulation of growth rate |
| WBGene00001996 | positive regulation of growth rate |
| WBGene00001996 | positive regulation of growth rate |
| WBGene00003230 | positive regulation of growth rate |
| WBGene00003738 | positive regulation of growth rate |
| WBGene00003882 | positive regulation of growth rate |
| WBGene00006206 | positive regulation of growth rate |
| WBGene00006349 | positive regulation of growth rate |
| WBGene00006437 | positive regulation of growth rate |
| WBGene00006935 | positive regulation of growth rate |
| WBGene00007013 | positive regulation of growth rate |
| WBGene00007972 | positive regulation of growth rate |
| WBGene00008119 | positive regulation of growth rate |
| WBGene00008934 | positive regulation of growth rate |
| WBGene00009668 | positive regulation of growth rate |
| WBGene00010015 | positive regulation of growth rate |
| WBGene00010562 | positive regulation of growth rate |
| WBGene00011696 | positive regulation of growth rate |
| WBGene00011831 | positive regulation of growth rate |
| WBGene00013720 | positive regulation of growth rate |
| WBGene00014114 | positive regulation of growth rate |
| WBGene00016142 | positive regulation of growth rate |
| WBGene00016444 | positive regulation of growth rate |
| WBGene00017319 | positive regulation of growth rate |
| WBGene00017804 | positive regulation of growth rate |
| WBGene00019353 | positive regulation of growth rate |
| WBGene00020128 | positive regulation of growth rate |
| WBGene00001204 | positive regulation of locomotion |
| WBGene00003882 | positive regulation of locomotion |
| WBGene00004391 | positive regulation of locomotion |
| WBGene00006779 | positive regulation of locomotion |
| WBGene00001831 | positive regulation of multicellular organism growth |
| WBGene00001974 | positive regulation of multicellular organism growth |
| WBGene00001996 | positive regulation of multicellular organism growth |
| WBGene00002280 | positive regulation of multicellular organism growth |
| WBGene00003026 | positive regulation of multicellular organism growth |
| WBGene00003882 | positive regulation of multicellular organism growth |
| WBGene00004859 | positive regulation of multicellular organism growth |
| WBGene00006779 | positive regulation of multicellular organism growth |
| WBGene00007013 | positive regulation of multicellular organism growth |
| WBGene00007025 | positive regulation of Notch signaling pathway |
| WBGene00001170 | positive regulation of programmed cell death |
| WBGene00001170 | positive regulation of programmed cell death |
| WBGene00001170 | positive regulation of programmed cell death |
| WBGene00001263 | positive regulation of protein secretion |
| WBGene00000871 | positive regulation of vulval development |
| WBGene00001996 | positive regulation of vulval development |
| WBGene00000871 | post-embryonic development |
| WBGene00011908 | primary microRNA processing |
| WBGene00002497 | procollagen-lysine 5-dioxygenase activity |
| WBGene00002368 | pronuclear migration |
| WBGene00010015 | pronuclear migration |
| WBGene00003738 | protein |
| WBGene00004053 | protein amino acid ADP-ribosylation |
| WBGene00007302 | protein amino acid dephosphorylation |
| WBGene00013810 | protein amino acid dephosphorylation |
| WBGene00014074 | protein amino acid dephosphorylation |
| WBGene00017627 | protein amino acid dephosphorylation |
| WBGene00006474 | protein amino acid methylation |
| WBGene00001532 | protein amino acid phosphorylation |
| WBGene00004859 | protein amino acid phosphorylation |
| WBGene00006436 | protein amino acid phosphorylation |
| WBGene00006437 | protein amino acid phosphorylation |
| WBGene00006779 | protein amino acid phosphorylation |
| WBGene00006814 | protein amino acid phosphorylation |
| WBGene00012352 | protein amino acid phosphorylation |
| WBGene00012785 | protein amino acid phosphorylation |
| WBGene00013978 | protein amino acid phosphorylation |
| WBGene00017803 | protein amino acid phosphorylation |
| WBGene00022635 | protein amino acid phosphorylation |
| WBGene00000377 | protein binding |
| WBGene00000378 | protein binding |
| WBGene00001170 | protein binding |
| WBGene00001831 | protein binding |
| WBGene00002025 | protein binding |
| WBGene00002184 | protein binding |
| WBGene00003026 | protein binding |
| WBGene00003789 | protein binding |
| WBGene00006445 | protein binding |
| WBGene00007025 | protein binding |
| WBGene00008081 | protein binding |
| WBGene00010139 | protein binding |
| WBGene00010621 | protein binding |
| WBGene00011908 | protein binding |
| WBGene00012606 | protein binding |
| WBGene00012785 | protein binding |
| WBGene00017144 | protein binding |
| WBGene00020316 | protein binding |
| WBGene00020629 | protein binding |
| WBGene00020859 | protein binding |
| WBGene00021924 | protein binding |
| WBGene00003230 | protein domain specific binding |
| WBGene00000377 | protein folding |
| WBGene00000378 | protein folding |
| WBGene00002025 | protein folding |
| WBGene00021201 | protein folding |
| WBGene00001532 | protein kinase activity |
| WBGene00004859 | protein kinase activity |
| WBGene00006436 | protein kinase activity |
| WBGene00006437 | protein kinase activity |
| WBGene00006779 | protein kinase activity |
| WBGene00006814 | protein kinase activity |
| WBGene00012352 | protein kinase activity |
| WBGene00012785 | protein kinase activity |
| WBGene00013978 | protein kinase activity |
| WBGene00022635 | protein kinase activity |
| WBGene00003230 | protein kinase binding |
| WBGene00010562 | protein kinase binding |
| WBGene00012352 | protein kinase C activity |
| WBGene00018393 | protein metabolic process |
| WBGene00000377 | protein refolding |
| WBGene00000378 | protein refolding |
| WBGene00002025 | protein refolding |
| WBGene00001532 | protein serine/threonine kinase activity |
| WBGene00004859 | protein serine/threonine kinase activity |
| WBGene00006436 | protein serine/threonine kinase activity |
| WBGene00006437 | protein serine/threonine kinase activity |
| WBGene00006779 | protein serine/threonine kinase activity |
| WBGene00006814 | protein serine/threonine kinase activity |
| WBGene00012352 | protein serine/threonine kinase activity |
| WBGene00013978 | protein serine/threonine kinase activity |
| WBGene00017803 | protein serine/threonine kinase activity |
| WBGene00022635 | protein serine/threonine kinase activity |
| WBGene00013124 | protein transport |
| WBGene00021027 | protein transport |
| WBGene00007302 | protein tyrosine phosphatase activity |
| WBGene00013810 | protein tyrosine phosphatase activity |
| WBGene00017627 | protein tyrosine phosphatase activity |
| WBGene00007302 | protein tyrosine/serine/threonine phosphatase activity |
| WBGene00014074 | protein tyrosine/serine/threonine phosphatase activity |
| WBGene00017627 | protein tyrosine/serine/threonine phosphatase activity |
| WBGene00001328 | proteinaceous extracellular matrix |
| WBGene00002035 | proteolysis |
| WBGene00008497 | proteolysis |
| WBGene00009645 | proteolysis |
| WBGene00018271 | proteolysis |
| WBGene00010988 | pteridine and derivative metabolic process |
| WBGene00006562 | pyridoxal phosphate binding |
| WBGene00001328 | receptor activity |
| WBGene00000196 | receptor-mediated endocytosis |
| WBGene00000377 | receptor-mediated endocytosis |
| WBGene00000378 | receptor-mediated endocytosis |
| WBGene00001263 | receptor-mediated endocytosis |
| WBGene00001328 | receptor-mediated endocytosis |
| WBGene00001483 | receptor-mediated endocytosis |
| WBGene00001595 | receptor-mediated endocytosis |
| WBGene00002047 | receptor-mediated endocytosis |
| WBGene00002130 | receptor-mediated endocytosis |
| WBGene00002497 | receptor-mediated endocytosis |
| WBGene00003026 | receptor-mediated endocytosis |
| WBGene00003134 | receptor-mediated endocytosis |
| WBGene00006206 | receptor-mediated endocytosis |
| WBGene00006935 | receptor-mediated endocytosis |
| WBGene00007564 | receptor-mediated endocytosis |
| WBGene00010015 | receptor-mediated endocytosis |
| WBGene00010562 | receptor-mediated endocytosis |
| WBGene00014114 | receptor-mediated endocytosis |
| WBGene00016142 | receptor-mediated endocytosis |
| WBGene00017319 | receptor-mediated endocytosis |
| WBGene00019353 | receptor-mediated endocytosis |
| WBGene00001874 | reciprocal meiotic recombination |
| WBGene00003421 | reciprocal meiotic recombination |
| WBGene00018138 | reduced folate carrier activity |
| WBGene00001996 | regulation of cell differentiation |
| WBGene00007025 | regulation of cell fate specification |
| WBGene00001328 | regulation of cell proliferation |
| WBGene00001481 | regulation of cell proliferation |
| WBGene00001483 | regulation of cell proliferation |
| WBGene00001595 | regulation of cell proliferation |
| WBGene00003026 | regulation of development, heterochronic |
| WBGene00011908 | regulation of development, heterochronic |
| WBGene00003026 | regulation of epidermal cell differentiation |
| WBGene00011908 | regulation of epidermal cell differentiation |
| WBGene00001481 | regulation of meiosis |
| WBGene00001483 | regulation of meiosis |
| WBGene00001595 | regulation of meiosis |
| WBGene00003134 | regulation of mitotic metaphase/anaphase transition |
| WBGene00001740 | regulation of multicellular organism growth |
| WBGene00006349 | regulation of protein catabolic process |
| WBGene00003230 | regulation of protein localization |
| WBGene00003230 | regulation of protein localization |
| WBGene00020316 | regulation of protein localization |
| WBGene00010776 | regulation of Rho protein signal transduction |
| WBGene00019832 | regulation of Rho protein signal transduction |
| WBGene00006571 | regulation of sister chromatid cohesion |
| WBGene00006571 | regulation of sister chromatid cohesion |
| WBGene00001204 | regulation of transcription |
| WBGene00007013 | regulation of transcription from RNA polymerase II promoter |
| WBGene00001310 | regulation of transcription, DNA-dependent |
| WBGene00006445 | regulation of transcription, DNA-dependent |
| WBGene00013124 | regulation of transcription, DNA-dependent |
| WBGene00043056 | regulation of transcription, DNA-dependent |
| WBGene00001481 | regulation of translation |
| WBGene00000196 | reproduction |
| WBGene00000377 | reproduction |
| WBGene00000378 | reproduction |
| WBGene00000871 | reproduction |
| WBGene00001263 | reproduction |
| WBGene00001310 | reproduction |
| WBGene00001328 | reproduction |
| WBGene00001483 | reproduction |
| WBGene00001595 | reproduction |
| WBGene00001651 | reproduction |
| WBGene00001740 | reproduction |
| WBGene00001831 | reproduction |
| WBGene00001974 | reproduction |
| WBGene00001996 | reproduction |
| WBGene00001996 | reproduction |
| WBGene00002025 | reproduction |
| WBGene00002047 | reproduction |
| WBGene00002130 | reproduction |
| WBGene00002280 | reproduction |
| WBGene00002368 | reproduction |
| WBGene00003026 | reproduction |
| WBGene00003134 | reproduction |
| WBGene00003499 | reproduction |
| WBGene00003789 | reproduction |
| WBGene00003882 | reproduction |
| WBGene00004391 | reproduction |
| WBGene00004723 | reproduction |
| WBGene00006206 | reproduction |
| WBGene00006307 | reproduction |
| WBGene00006474 | reproduction |
| WBGene00006481 | reproduction |
| WBGene00006935 | reproduction |
| WBGene00007000 | reproduction |
| WBGene00007013 | reproduction |
| WBGene00007972 | reproduction |
| WBGene00008081 | reproduction |
| WBGene00008119 | reproduction |
| WBGene00009668 | reproduction |
| WBGene00010015 | reproduction |
| WBGene00010562 | reproduction |
| WBGene00011350 | reproduction |
| WBGene00011696 | reproduction |
| WBGene00016142 | reproduction |
| WBGene00016816 | reproduction |
| WBGene00017319 | reproduction |
| WBGene00019792 | reproduction |
| WBGene00020068 | reproduction |
| WBGene00020316 | reproduction |
| WBGene00022257 | reproduction |
| WBGene00022881 | reproduction |
| WBGene00016700 | response to oxidative stress |
| WBGene00002025 | response to reactive oxygen species |
| WBGene00002263 | response to stress |
| WBGene00010776 | Rho guanyl-nucleotide exchange factor activity |
| WBGene00019832 | Rho guanyl-nucleotide exchange factor activity |
| WBGene00009163 | ribonuclease III activity |
| WBGene00004391 | ribonucleoside-diphosphate reductase activity |
| WBGene00004391 | ribonucleoside-diphosphate reductase complex |
| WBGene00016142 | ribosome |
| WBGene00017319 | ribosome |
| WBGene00001595 | RNA binding |
| WBGene00008061 | RNA binding |
| WBGene00009163 | RNA binding |
| WBGene00018857 | RNA binding |
| WBGene00022257 | RNA binding |
| WBGene00003499 | RNA interference |
| WBGene00006474 | RNA interference |
| WBGene00009668 | RNA interference |
| WBGene00015150 | RNA polymerase II transcription factor activity |
| WBGene00007013 | RNA polymerase II transcription mediator activity |
| WBGene00006307 | RNA processing |
| WBGene00009163 | RNA processing |
| WBGene00007972 | RNA splicing |
| WBGene00002497 | secretion by cell |
| WBGene00010988 | secretion by cell |
| WBGene00012538 | selenium binding |
| WBGene00005681 | sensory perception of chemical stimulus |
| WBGene00001204 | sequence-specific DNA binding |
| WBGene00001310 | sequence-specific DNA binding |
| WBGene00018271 | serine-type carboxypeptidase activity |
| WBGene00008497 | serine-type peptidase activity |
| WBGene00007620 | signal transduction |
| WBGene00019811 | single fertilization |
| WBGene00020316 | single-stranded DNA binding |
| WBGene00001595 | single-stranded RNA binding |
| WBGene00013124 | small GTPase mediated signal transduction |
| WBGene00021027 | small GTPase mediated signal transduction |
| WBGene00006437 | small GTPase regulator activity |
| WBGene00022881 | social behavior |
| WBGene00010334 | sodium channel activity |
| WBGene00010334 | sodium ion transport |
| WBGene00012352 | soluble fraction |
| WBGene00001483 | spermatid development |
| WBGene00001481 | spermatogenesis |
| WBGene00016960 | spermatogenesis |
| WBGene00007013 | Srb-mediator complex |
| WBGene00020316 | strand invasion |
| WBGene00000959 | striated |
| WBGene00006349 | striated |
| WBGene00000634 | structural constituent of cuticle |
| WBGene00000687 | structural constituent of cuticle |
| WBGene00000722 | structural constituent of cuticle |
| WBGene00000753 | structural constituent of cuticle |
| WBGene00016142 | structural constituent of ribosome |
| WBGene00017319 | structural constituent of ribosome |
| WBGene00017876 | structural constituent of vitelline membrane |
| WBGene00001874 | structural molecule activity |
| WBGene00002055 | structural molecule activity |
| WBGene00002280 | structural molecule activity |
| WBGene00017042 | structural molecule activity |
| WBGene00018840 | structural molecule activity |
| WBGene00003738 | synapse organization |
| WBGene00003738 | synaptic transmission, cholinergic |
| WBGene00006779 | synaptic transmission, GABAergic |
| WBGene00001310 | transcription activator activity |
| WBGene00003480 | transcription activator activity |
| WBGene00001204 | transcription factor activity |
| WBGene00001310 | transcription factor activity |
| WBGene00003480 | transcription factor activity |
| WBGene00006445 | transcription factor activity |
| WBGene00043056 | transcription factor activity |
| WBGene00002025 | transcription factor binding |
| WBGene00013124 | transcription factor binding |
| WBGene00006445 | transcription factor complex |
| WBGene00015150 | transcription factor TFIIA complex |
| WBGene00015150 | transcription initiation from RNA polymerase II promoter |
| WBGene00018295 | transferase activity, transferring acyl groups other than amino-acyl groups |
| WBGene00007422 | transferase activity, transferring hexosyl groups |
| WBGene00011893 | transferase activity, transferring phosphorus-containing groups |
| WBGene00006445 | transforming growth factor beta receptor signaling pathway |
| WBGene00000196 | translation |
| WBGene00006935 | translation |
| WBGene00016142 | translation |
| WBGene00017319 | translation |
| WBGene00007000 | translation elongation factor activity |
| WBGene00007000 | translational elongation |
| WBGene00005681 | transmembrane receptor activity |
| WBGene00001651 | transmembrane transport |
| WBGene00007347 | transmembrane transport |
| WBGene00020207 | transmembrane transport |
| WBGene00004062 | transport |
| WBGene00018138 | transport |
| WBGene00020207 | transport |
| WBGene00019520 | triacylglycerol lipase activity |
| WBGene00000196 | tRNA aminoacylation |
| WBGene00006935 | tRNA aminoacylation for protein translation |
| WBGene00001740 | tRNA isopentenyltransferase activity |
| WBGene00001740 | tRNA isopentenyltransferase activity |
| WBGene00001740 | tRNA modification |
| WBGene00001740 | tRNA processing |
| WBGene00000377 | unfolded protein binding |
| WBGene00000378 | unfolded protein binding |
| WBGene00002025 | unfolded protein binding |
| WBGene00006935 | valine-tRNA ligase activity |
| WBGene00006935 | valyl-tRNA aminoacylation |
| WBGene00016960 | vesicle docking during exocytosis |
| WBGene00001874 | vesicle membrane |
| WBGene00016960 | vesicle-mediated transport |
| WBGene00017876 | vitelline membrane formation |
| WBGene00001204 | vulval development |
| WBGene00001996 | vulval development |
| WBGene00003480 | vulval development |
| WBGene00011908 | vulval development |
| WBGene00001310 | zinc ion binding |
| WBGene00001481 | zinc ion binding |
| WBGene00001740 | zinc ion binding |
| WBGene00003026 | zinc ion binding |
| WBGene00003230 | zinc ion binding |
| WBGene00003480 | zinc ion binding |
| WBGene00007772 | zinc ion binding |
| WBGene00008081 | zinc ion binding |
| WBGene00010988 | zinc ion binding |
| WBGene00011696 | zinc ion binding |
| WBGene00012606 | zinc ion binding |
| WBGene00017144 | zinc ion binding |
| WBGene00020629 | zinc ion binding |
| WBGene00021924 | zinc ion binding |
| **^a^**Over-represented categories shown in **bold.** | |
