# Supplementary material for: SPE-44 Implements Sperm Cell Fate
Source: PLoS Genet. 2012 Apr 26;8(4):e1002678. doi: 10.1371/journal.pgen.1002678 (PMC3343087; doi:10.1371/journal.pgen.1002678)
Supplement: Table S5 — Oligonucleotide sequences. Listed are primers used for single-worm PCR, quantitative RT-PCR, and plasmid constructions. (DOC) [file pgen.1002678.s009.doc]

| **Table S5. Oligonucleotides for PCR assays** | |
| --- | --- |
| **Name** | **Sequence (5’->3’)** |
| HES-343 | CCAGTATTACGTTGTGACGAG |
| HES-364 | TCACGGTTTTATTCGGATTG |
| HES-502 | GATGGAGCATTCACATAATTTC |
| HES-531 | AAAAACTAGTATGTTCGGTGGAGACGTG |
| HES-532 | AAAAGGATCCTTATTGAATATGACTAGGTCCTGG |
| HES-539 | ACGATCTTCTTTCTCCGAAG |
| HES-583 | AAAAGAATTCGATATCCTCTAGCGCTCAATC |
| HES-584 | AAAACTAGTTTTAGACAGGTAATAAAATTAATATTAGCA |
| HES-612 | AAAGAATTCGGAGTCCAGTGACGATTCTTTTGAAGAATTTTG |
| HES-613 | AAACTCGAGTGTTCGATTGGGCGACGG |
| AKA-70 | GGTGGTTCCTCCGGAAAGAA |
| AKA-71 | GCTATGTTCCAGCCATCCTTCT |
